# The global burden of mental and substance use disorders among adolescents and young adults

**DOI:** 10.1038/s41380-026-03503-9

**Published:** 2026-02-28

**Authors:** Xiangyu Zhao, Ligang Liu, Luofei Zhang, Pujing Zhao, Wenshuo Jiang, Milap C. Nahata

**Affiliations:** 1 Department of Applied Psychology, Bouvé College of Health Sciences, Northeastern University, Boston, USA; 2 Department of Pharmacy Practice and Administration, College of Pharmacy, Western University of Health Sciences, Pomona, CA 91766 USA; 3 Institute of Therapeutic Innovations and Outcomes (ITIO), College of Pharmacy, The Ohio State University, Columbus, OH USA; 4 Department of Pharmacy, Beijing Tiantan Hospital, Capital Medical University, Beijing, China; 5 College of Pharmacy, The Ohio State University, Columbus, OH USA; 6 College of Medicine, The Ohio State University, Columbus, OH USA

**Keywords:** ADHD, Autism spectrum disorders, Depression

## Abstract

Mental disorders and substance use disorders (SUDs) are significant public health challenges among adolescents and young adults (AYAs). This study quantified global and regional prevalence, disability-adjusted life-years (DALYs), and years lived with disability (YLDs), and temporal trends of mental disorders and SUDs among AYAs from 1990–2021. Estimates were generated using DisMod-MR 2.1, with standardized case definitions, severity weights, and pandemic-period adjustments. Temporal trends were assessed with Joinpoint regression. In 2021, the global point prevalence of mental disorders was 15.2% in adolescents, and 16.1% among young adults, with anxiety disorders being the most common condition. Mental disorders were the leading cause of YLDs and DALYs in 2021. High-income regions exhibited the highest rates of mental disorders. Significant sex differences were observed, with higher prevalence of attention-deficit hyperactivity disorder and autism spectrum disorders in males, and anorexia nervosa was more prevalent in females. SUDs were the 15th cause of YLDs and 22nd cause of DALYs among adolescents and ranked 8th for YLDs and 11th for DALYs for young adults. During the pandemic, a significant increase in the prevalence of mental disorders and a decline in SUDs was observed. DALYs for mental disorders increased significantly during the pandemic, especially for depressive and anxiety disorders, with higher DALYs in females than males. Mental disorders remain a leading cause of disability in AYAs worldwide, with a marked surge during 2019–2021. Although SUDs declined, the rising burden of depression and anxiety demonstrated urgent needs for age-specific, sex-responsive, and regionally tailored mental health strategies.

## Introduction

Mental disorders and substance use disorders (SUDs) represent critical and escalating public health issues, particularly among adolescents and young adults [1, 2]. These age groups undergo profound neurobiological, hormonal, and psychosocial transitions that increase vulnerability to both mental health issues and high-risk substance use [3]. According to the World Health Organization (WHO), mental disorders affected approximately 14% of adolescents worldwide, while SUDs were estimated to impact 2.2% of this population in 2019, underscoring the substantial global burden carried by young people [4, 5].

Mental disorders, including depression, anxiety, attention-deficit hyperactivity disorder (ADHD), idiopathic developmental intellectual disability (IDID), and others, typically emerge during adolescence and often persist into adulthood, impairing social, academic, and functional outcomes [6, 7]. Concurrently, SUDs, like alcohol use disorders (AUDs) and drug use disorders (DUDs), frequently coexist with mental disorders [8]. The interaction between mental disorders and SUDs contributes to social marginalization, school dropout, self-harm, and premature mortality [9].

Evidence from the Global Burden of Disease (GBD) 2019 study indicated that the prevalence and disability burden attributable to mental disorders among individuals aged 10–24 years increased steadily over the past three decades [10]. The proportion of disability-adjusted life years (DALYs) attributable to mental disorders rose from 3.1% in 1990 to 4.9% in 2019 [10]. Similarly, the prevalence of SUDs has also shown a notable increase, particularly among young males [11]. These trends reflected shifting global patterns in social stressors, conflict, urbanization, substance availability, and socioeconomic instability. Yet data specific to adolescents and young adults remained sparse, particularly for recent years.

The COVID-19 pandemic further intensified mental health needs among youth by disrupting schooling, social relationships, family stability, and access to mental health services [12]. Global reports suggested increases in depressive symptoms, anxiety, and problematic substance use during the pandemic years [13], but the extent to which these changes contributed to the global burden of mental disorders and SUDs remained unclear. Many national studies documented worsening mental health among youth after 2020, but consistent, comparable global estimates were lacking. Despite the urgent policy attention to youth mental health, a critical knowledge gap persisted regarding the global and regional epidemiology of mental disorders and SUDs among adolescents (10–19 years) and young adults (20–24 years) during the past decade, particularly in post-pandemic period. Updated, age-stratified, and sex-stratified global estimates are necessary to guide early intervention, allocate resources efficiently, and support national mental health strategies that increasingly prioritize children and young adults.

This study aimed to use the 2021 GBD dataset to 1) estimate the global and regional prevalence of mental disorders and SUDs among adolescents and young adults from 1990 to 2021; 2) quantify the disability burden attributable to these conditions and identify age and sex differences as the leading contributors; 3) examine temporal trends from 1990 to 2021, with particular focus on changes between 2019 and 2021 to capture potential pandemic-related shifts.

## Methods

### Data sources

The 2021 GBD led by the Institute for Health Metrics and Evaluation (IHME), quantifies health trends worldwide across 204 countries and territories (1990–2021), covering 288 causes of mortality, 371 diseases and injuries, and their disability outcomes. Data were compiled from vital registration systems, household surveys, disease registries, hospital admissions, and published literature, accessible through the Global Health Data Exchange platform. In 2021 GBD, diseases and injuries and causes of death, were aggregated in 4 level 1 causes (communicable, maternal, neonatal, and nutritional diseases; non-communicable diseases; injuries; and other COVID-19 pandemic-related outcomes), 22 level 2 causes, 175 level 3 causes, and 302 level 4 causes [14].

### Diseases definitions and classification

Mental disorders and SUDs were defined according to the GBD 2021 protocol, which was based on Diagnostic and Statistical Manual of Mental Disorders (DSM) and the International Classification of Diseases (ICD) diagnostic criteria, with all methodological details aligned to its standardized frameworks for non-fatal outcome estimation, statistical modeling, and severity assessment [15]. We analyzed 10 mental disorders and 2 SUDs (GBD Level 3, non-communicable diseases). The anxiety disorders category specifically included panic disorder, agoraphobia, specific phobia, social phobia, obsessive-compulsive disorder, post-traumatic stress disorder (PTSD), generalized anxiety disorder, separation anxiety disorder, and anxiety disorder not otherwise specified. Depressive disorders consisted of major depressive disorder (MDD) and dysthymia. Other disorders analyzed were ADHD, autism spectrum disorders (ASD), bipolar disorder, conduct disorder, eating disorders, IDID, schizophrenia, other mental disorders, AUDs and DUDs. The specific ICD-10 codes and diagnostic features for each disorder are provided in Table S1.

### Case ascertainment and data processing

This study adopted the data collection, processing, and overall analytical methodologies established by the GBD 2021 Study [14–17]. Case identification integrated multiple data sources, including population-based surveys using validated diagnostic instruments including the Composite International Diagnostic Interview for adult mental disorders and the Development and Well-Being Assessment for youth aged 10–19 years, clinical inpatient records from 49 countries and insurance claims data, all standardized to ICD-10 codes. All prevalence estimates in this study refer to point prevalence (hereafter referred to as prevalence), defined as the proportion of individuals meeting diagnostic criteria at a specific time point. Data collected using alternative recall periods (e.g., past-year prevalence) were adjusted to point prevalence equivalents through crosswalking using meta-regression Bayesian regularized trimmed (MR-BRT) models. Age-sex splitting was applied to disaggregate broadly grouped data using modeled age patterns and sex ratios derived from MR-BRT analyses. Age of onset patterns were established based on epidemiological evidence and clinical understanding of each disorder’s natural history. For data-sparse regions, DisMod-MR 2.1 used higher-geography priors (e.g., super-region estimates) to standardize age-of-onset stratification.

### Burden of disease estimation metrics

Consistent with GBD 2021 methodologies, the overall disease burden was expressed in DALYs, constructed by combining Years of Life Lost (YLLs) and Years Lived with Disability (YLDs) [17]. YLLs were calculated from cause-specific deaths and standard life expectancy [17]. YLDs were estimated by multiplying disorder prevalence by corresponding disability weight (DW) [17]. DWs valued from 0 (perfect health) to 1 (death), were derived from multinational paired-comparison surveys that compared the severity of different health states. Comorbidity adjustments were made using microsimulation to account for concurrent conditions [15].

### Methodological approach to the COVID-19 pandemic period in GBD 2021

Although the GBD 2021 methods appendix does not contain a single, formal sentence declaring the “pandemic period” as January 2020 to December 2021, the entire methodological architecture, data processing pipeline, and model application are unequivocally built upon the foundation of using 2020 and 2021 as the assessment period for the impact of the COVID-19 pandemic [15]. This approach substantively covered the key phase of the global spread of SARS-CoV-2 and aligned with the overall data cycle of GBD 2021 (1990–2021), thereby focusing on the short-term shock of the pandemic on health burden. To capture global variation in pandemic impact, a location-specific modeling strategy was employed instead of applying a uniform timeline. For non-fatal outcomes like anxiety and depressive disorders, two time-varying indicators were used: reductions in human mobility derived from anonymized cell phone data (a proxy for social restrictions) and daily COVID-19 mortality rates from IHME estimates, which captured the direct viral impact. An MR-BRT model predicted daily prevalence changes based on these indicators, which was subsequently used to adjust the pre-pandemic baseline estimates for each day of 2020 and 2021, for every age-sex-location group. To ensure comparability with the historical GBD time series (1990–2019), 2019 served as the pre-pandemic baseline for contextualizing 2020–2021 estimates. High-frequency pandemic data were weighted and averaged to generate standard annual point estimates, maintaining structural consistency with the full time series for valid trend analysis. Therefore, reported burden changes reflect quantified, location-specific pandemic impact that capture global heterogeneity while preserving methodological consistency.

### Evolution of GBD estimation methods

The analytical framework for mental and SUD in GBD studies has evolved, while maintaining consistency in core methodological principles. A key transition from ICD-9 (1990–1999) to the ICD-10 (2000–2021) was accompanied by a standardized diagnostic crosswalk mapping ICD-9 to ICD-10 codes, preserving consistency in diagnostic thresholds for mental disorders and SUDs. Modeling tools advanced from DisMod-MR 1.0 (GBD 2010) to 2.1 (GBD 2013 onward), with MR-BRT introduced in GBD 2019 for sophisticated bias adjustment of alternative case definitions and study methods. Recent innovations have included modeling COVID-19 impacts using mobility and excess mortality. Throughout these developments, fundamental elements have remained consistent, including DSM/ICD diagnostic criteria, DW from paired-comparison surveys, the DALY framework for burden quantification, and point prevalence as the reference metric, ensuring the comparability of estimates across the 1990–2021 study period.

### Statistical modeling approach

The study employed DisMod-MR 2.1, a Bayesian meta-regression tool, to generate estimates through a multi-level, geographical cascade from global to subnational levels [16]. We examined prevalence, DALYs, and YLDs for adolescents (10–19 years) and young adults (20–24 years) by sex and region. Descriptive analyses characterized burden metrics of mental disorders and SUDs by sex, age and year, with 95% uncertainty intervals (UI) for all estimates. Temporal trends were assessed using Joinpoint regression to calculate annual percentage change (APC) and average annual percentage change (AAPC), along with corresponding 95% confidence interval (CI) [18]. Additionally, projections of DALYs due to mental disorders and SUDs were made through to the year 2050. Statistical significance was determined at p < 0.05 using Wald Chi-square tests.

## Results

### Disease burden of mental and substance use disorders in 2021

#### Prevalence of mental and substance use disorders

Globally, mental disorders affected an estimated 184.9 million adolescents (95% UI 161.3–210.2) and 94.1 million young adults (95% UI 81.5–107.7) in 2021 (Table S2). Anxiety disorders were the most prevalent conditions in both adolescents (4.94%, 95% UI 3.70–6.46) and young adults (5.80%, 95% UI 4.27–7.65), followed by depressive disorders (adolescents: 2.43%, UI 1.77–3.16; young adults: 4.79%, 95% UI 3.59–6.62) (Tables 1 and 2). A notable sex-based pattern reversal was observed: while males had a higher overall prevalence in adolescence (15.74% vs. 14.65%), females exhibited a higher prevalence in young adulthood (17.21% vs. 15.05%). Among the specific mental disorders, sex-specific patterns were largely consistent across both age groups. Schizophrenia, bipolar disorder, and IDID showed comparable prevalence between sexes, whereas depressive disorders, anxiety disorders, and eating disorders were consistently more prevalent in females. In contrast, ASD, ADHD, conduct disorder, and other mental disorders were more common in males. Additionally, 10.1 million adolescents and 19.6 million young adults had SUDs, with males having higher prevalence rates for both AUDs and DUDs in two age groups (Tables S2, 1 and 2).

**Table 1:** Global prevalence, proportion of total DALYs, proportion of total YLDs for mental disorders and substance use disorders, age 10–19 in 2021 with temporal changes (1990–2019 and 2019–2021).

Conditions	Prevalence % (95% UI)	Percentage change in Prevalence rates		Proportion of total DALYs % (95% UI)	Percentage change in DALYs rates		Proportion of total YLDs % (95% UI)	Percentage change in YLDs rates	
	2021	1990–2019	2019–2021	2021	1990–2019	2019–2021	2021	1990–2019	2019–2021
**Mental disorders**									
Both	15.20 (13.25, 17.28)	−2.92%	8.79%	15.19 (12.00, 18.87)	1.01%	12.76%	27.14 (22.26, 33.44)	1.01%	12.77%
Male	15.74 (13.74, 17.85)	−4.04%	6.33%	14.31 (11.44, 17.56)	0.90%	10.00%	28.74 (23.71, 34.72)	0.90%	10.00%
Female	14.65 (12.72, 16.74)	−1.69%	11.60%	16.11 (12.77, 20.21)	1.18%	15.48%	25.79 (20.89, 32.33)	1.18%	15.48%
**Schizophrenia**									
Both	0.04 (0.03, 0.06)	−4.48%	0.13%	0.23 (0.14, 0.36)	−4.24%	−0.19%	0.41 (0.23, 0.66)	−4.24%	−0.19%
Male	0.04 (0.03, 0.07)	−4.12%	−0.05%	0.25 (0.15, 0.38)	−3.80%	−0.25%	0.50 (0.28, 0.79)	−3.80%	−0.25%
Female	0.04 (0.02, 0.06)	−5.03%	0.33%	0.21 (0.12, 0.34)	−4.96%	−0.04%	0.34 (0.19, 0.56)	−4.96%	−0.04%
**Depressive disorders**									
Both	2.43 (1.77, 3.16)	4.46%	23.14%	3.78 (2.53, 5.46)	4.47%	26.23%	6.76 (4.62, 9.73)	4.47%	26.23%
Male	1.88 (1.36, 2.46)	6.66%	22.72%	2.87 (1.89, 4.17)	7.17%	26.00%	5.75 (3.96, 8.27)	7.17%	26.00%
Female	2.98 (2.18, 3.86)	3.51%	23.39%	4.75 (3.20, 6.74)	3.24%	26.35%	7.60 (5.19, 10.88)	3.24%	26.35%
**Major depressive disorder**									
Both	1.96 (1.34, 2.60)	3.99%	30.47%	3.39 (2.15, 4.99)	4.21%	30.15%	6.07 (4.01, 8.89)	4.21%	30.15%
Male	1.50 (1.01, 1.99)	7.08%	30.43%	2.55 (1.57, 3.80)	7.39%	30.16%	5.12 (3.36, 7.58)	7.39%	30.16%
Female	2.44 (1.68, 3.23)	2.59%	30.48%	4.28 (2.75, 6.20)	2.73%	30.13%	6.86 (4.52, 10.08)	2.73%	30.13%
**Dysthymia**									
Both	0.48 (0.33, 0.68)	6.04%	0.05%	0.39 (0.25, 0.59)	6.28%	−0.09%	0.69 (0.45, 1.02)	6.28%	−0.09%
Male	0.39 (0.27, 0.56)	5.49%	0.12%	0.31 (0.20, 0.49)	5.79%	0.06%	0.63 (0.41, 0.92)	5.79%	0.06%
Female	0.56 (0.39, 0.80)	6.76%	−0.01%	0.47 (0.30, 0.69)	6.94%	−0.19%	0.74 (0.49, 1.12)	6.94%	−0.19%
**Bipolar disorder**									
Both	0.31 (0.22, 0.43)	2.15%	−0.34%	0.57 (0.36, 0.89)	2.45%	−0.58%	1.02 (0.63, 1.61)	2.45%	−0.58%
Male	0.30 (0.22, 0.42)	2.28%	−0.32%	0.55 (0.35, 0.85)	2.65%	−0.54%	1.11 (0.68, 1.73)	2.65%	−0.54%
Female	0.32 (0.22, 0.44)	2.10%	−0.37%	0.59 (0.37, 0.93)	2.33%	−0.63%	0.94 (0.58, 1.51)	2.33%	−0.63%
**Anxiety disorders**									
Both	4.94 (3.70, 6.46)	−3.25%	23.55%	5.00 (3.41, 6.87)	−3.10%	23.35%	8.92 (6.26, 12.19)	−3.10%	23.35%
Male	3.88 (2.87, 5.13)	−3.10%	22.93%	3.87 (2.55, 5.43)	−2.88%	22.78%	7.77 (5.39, 10.65)	−2.88%	22.78%
Female	6.01 (4.50, 7.85)	−3.00%	23.93%	6.18 (4.25, 8.44)	−2.90%	23.71%	9.89 (6.91, 13.54)	−2.90%	23.71%
**Eating disorders**									
Both	0.27 (0.18, 0.41)	15.27%	−0.32%	0.48 (0.30, 0.76)	15.36%	−0.46%	0.86 (0.54, 1.35)	15.44%	−0.46%
Male	0.21 (0.13, 0.32)	18.70%	0.43%	0.36 (0.21, 0.58)	18.89%	0.39%	0.72 (0.43, 1.15)	18.89%	0.39%
Female	0.34 (0.23, 0.52)	13.80%	−0.79%	0.62 (0.39, 0.95)	13.87%	−1.00%	0.98 (0.62, 1.54)	13.92%	−0.99%
**Anorexia nervosa**									
Both	0.10 (0.06, 0.15)	8.75%	−0.32%	0.18 (0.11, 0.28)	8.83%	−0.43%	0.32 (0.19, 0.50)	8.99%	−0.44%
Male	0.06 (0.04, 0.10)	10.62%	0.00%	0.11 (0.06, 0.17)	10.90%	−0.08%	0.22 (0.13, 0.35)	11.01%	−0.08%
Female	0.14 (0.09, 0.21)	8.54%	−0.48%	0.25 (0.15, 0.40)	8.57%	−0.61%	0.40 (0.24, 0.63)	8.68%	−0.58%
**Bulimia nervosa**									
Both	0.17 (0.09, 0.31)	19.44%	−0.33%	0.31 (0.16, 0.57)	19.57%	−0.48%	0.54 (0.27, 1.02)	19.57%	−0.48%
Male	0.14 (0.08, 0.26)	22.62%	0.63%	0.25 (0.13, 0.46)	22.76%	0.60%	0.50 (0.25, 0.96)	22.76%	0.60%
Female	0.20 (0.11, 0.36)	17.70%	−1.01%	0.36 (0.19, 0.68)	17.84%	−1.27%	0.58 (0.30, 1.09)	17.84%	−1.27%
**Autism spectrum disorders**									
Both	0.89 (0.75, 1.04)	2.78%	0.14%	1.40 (0.95, 2.00)	3.04%	0.04%	2.53 (1.58, 3.77)	3.04%	0.04%
Male	1.20 (1.02, 1.40)	1.58%	0.12%	1.87 (1.27, 2.65)	1.86%	0.03%	3.78 (2.40, 5.57)	1.86%	0.03%
Female	0.57 (0.48, 0.68)	4.36%	0.23%	0.92 (0.61, 1.33)	4.57%	0.09%	1.48 (0.91, 2.23)	4.57%	0.09%
**ADHD**									
Both	2.59 (1.78, 3.68)	−11.27%	0.19%	0.26 (0.14, 0.41)	−11.15%	0.13%	0.46 (0.27, 0.72)	−11.15%	0.13%
Male	3.75 (2.59, 5.28)	−11.75%	0.40%	0.37 (0.20, 0.58)	−11.62%	0.36%	0.74 (0.43, 1.15)	−11.62%	0.36%
Female	1.42 (0.97, 2.05)	−11.28%	−0.31%	0.14 (0.08, 0.23)	−11.20%	−0.42%	0.23 (0.14, 0.37)	−11.20%	−0.42%
**Conduct disorder**									
Both	2.72 (1.90, 3.58)	3.54%	0.02%	2.71 (1.63, 4.04)	3.84%	−0.08%	4.82 (3.03, 7.08)	3.84%	−0.08%
Male	3.47 (2.49, 4.46)	2.05%	0.02%	3.40 (2.05, 5.03)	2.40%	−0.07%	6.81 (4.31, 9.88)	2.40%	−0.07%
Female	1.95 (1.32, 2.65)	5.57%	0.05%	1.97 (1.15, 3.04)	5.78%	−0.07%	3.16 (1.89, 4.77)	5.78%	−0.07%
**IDID**									
Both	1.72 (0.97, 2.44)	−11.93%	−1.29%	0.60 (0.31, 0.95)	−6.37%	−0.89%	1.07 (0.57, 1.59)	−6.37%	−0.89%
Male	1.72 (0.92, 2.51)	−14.98%	−1.36%	0.59 (0.28, 0.97)	−10.15%	−0.94%	1.19 (0.60, 1.81)	−10.15%	−0.94%
Female	1.72 (1.03, 2.38)	−8.64%	−1.22%	0.61 (0.33, 0.93)	−2.19%	−0.83%	0.97 (0.54, 1.41)	−2.19%	−0.83%
**Other mental disorders**									
Both	0.24 (0.15, 0.35)	−0.45%	−0.13%	0.15 (0.09, 0.24)	−0.22%	−0.23%	0.28 (0.17, 0.42)	−0.22%	−0.23%
Male	0.30 (0.19, 0.44)	−0.70%	−0.11%	0.19 (0.12, 0.29)	−0.46%	−0.23%	0.38 (0.24, 0.57)	−0.46%	−0.23%
Female	0.18 (0.11, 0.26)	−0.60%	−0.14%	0.12 (0.07, 0.18)	−0.44%	−0.22%	0.19 (0.11, 0.29)	−0.44%	−0.22%
**Substance use disorders**									
Both	0.83 (0.62, 1.09)	−14.27%	−3.76%	0.78 (0.61, 0.95)	−23.32%	−1.59%	1.09 (0.79, 1.40)	−13.52%	−2.30%
Male	1.08 (0.82, 1.41)	−14.08%	−4.78%	0.98 (0.78, 1.21)	−21.69%	−2.10%	1.53 (1.11, 1.95)	−14.18%	−2.83%
Female	0.57 (0.43, 0.75)	−15.32%	−1.73%	0.56 (0.44, 0.69)	−26.68%	−0.59%	0.72 (0.52, 0.95)	−12.92%	−1.34%
**Alcohol use disorders**									
Both	0.30 (0.20, 0.43)	−20.72%	−3.12%	0.28 (0.18, 0.41)	−27.30%	−3.53%	0.45 (0.27, 0.66)	−21.02%	−3.36%
Male	0.42 (0.28, 0.59)	−23.12%	−2.95%	0.39 (0.26, 0.57)	−28.29%	−3.36%	0.68 (0.43, 1.01)	−23.31%	−3.20%
Female	0.18 (0.12, 0.27)	−15.79%	−3.45%	0.17 (0.10, 0.25)	−25.70%	−3.91%	0.25 (0.15, 0.38)	−16.22%	−3.82%
**Drug use disorders**									
Both	0.53 (0.36, 0.75)	−10.34%	−4.14%	0.49 (0.40, 0.61)	−20.75%	−0.42%	0.64 (0.47, 0.87)	−7.27%	−1.53%
Male	0.67 (0.45, 0.96)	−7.82%	−5.88%	0.59 (0.47, 0.72)	−16.40%	−1.24%	0.85 (0.62, 1.12)	−4.96%	−2.53%
Female	0.39 (0.27, 0.55)	−15.11%	−0.92%	0.39 (0.31, 0.49)	−27.11%	0.90%	0.47 (0.34, 0.63)	−10.98%	0.03%

95% UI, 95% uncertainty interval.

*YLDs* years lived with disability, *DALYs* disability-adjusted life years, *ADHD* attention-deficit/hyperactivity disorder, *IDID* idiopathic developmental intellectual disability.

All prevalence estimates in this study refer to point prevalence.

**Table 2:** Global prevalence, proportion of total DALYs, proportion of total YLDs for mental disorders and substance use disorders, age 20–24 in 2021 with temporal changes (1990–2019 and 2019–2021).

Conditions	Prevalence % (95% UI)	Percentage change in Prevalence rates		Proportion of total DALYs % (95% UI)	Percentage change in DALYs rates		Proportion of total YLDs % (95% UI)	Percentage change in YLDs rates	
	2021	1990–2019	2019–2021	2021	1990–2019	2019–2021	2021	1990–2019	2019–2021
**Mental disorders**									
Both	16.13 (13.96, 18.45)	−2.83%	12.25%	13.48 (10.71, 16.60)	−0.65%	15.01%	26.62 (21.95, 32.87)	−0.65%	15.01%
Male	15.05 (13.11, 17.03)	−3.13%	9.81%	11.59 (9.36, 14.21)	1.08%	12.95%	27.92 (23.40, 34.01)	1.09%	12.95%
Female	17.21 (14.75, 19.80)	−2.48%	14.49%	15.53 (12.48, 19.40)	−1.88%	16.74%	25.66 (20.86, 32.04)	−1.88%	16.75%
**Schizophrenia**									
Both	0.26 (0.17, 0.38)	−3.45%	−0.45%	0.98 (0.60, 1.46)	−3.15%	−0.89%	1.94 (1.14, 2.94)	−3.15%	−0.89%
Male	0.28 (0.18, 0.40)	−2.98%	−0.52%	1.03 (0.64, 1.53)	−2.76%	−0.81%	2.49 (1.47, 3.72)	−2.76%	−0.81%
Female	0.24 (0.15, 0.35)	−4.09%	−0.39%	0.92 (0.56, 1.38)	−3.71%	−1.02%	1.53 (0.89, 2.36)	−3.71%	−1.02%
**Depressive disorders**									
Both	4.79 (3.59, 6.62)	−3.26%	20.84%	4.99 (3.42, 7.18)	−4.54%	24.31%	9.83 (6.88, 13.81)	−4.54%	24.31%
Male	3.85 (2.88, 5.26)	1.33%	20.51%	3.90 (2.66, 5.63)	0.89%	24.16%	9.37 (6.51, 13.10)	0.89%	24.16%
Female	5.73 (4.33, 7.90)	−5.86%	21.11%	6.16 (4.24, 8.82)	−7.64%	24.46%	10.17 (7.04, 14.32)	−7.64%	24.46%
**Major depressive disorder**									
Both	3.62 (2.51, 5.38)	−6.67%	29.60%	4.30 (2.79, 6.43)	−6.43%	29.16%	8.48 (5.73, 12.51)	−6.43%	29.16%
Male	2.89 (2.03, 4.27)	−0.44%	29.33%	3.35 (2.16, 5.01)	−0.11%	29.06%	8.05 (5.41, 11.87)	−0.11%	29.06%
Female	4.36 (3.00, 6.51)	−10.12%	29.83%	5.33 (3.45, 7.99)	−10.01%	29.27%	8.79 (5.88, 12.99)	−10.01%	29.27%
**Dysthymia**									
Both	1.22 (0.85, 1.68)	5.74%	0.80%	0.68 (0.43, 1.00)	5.98%	0.58%	1.35 (0.89, 1.94)	5.98%	0.58%
Male	0.99 (0.69, 1.37)	5.65%	0.77%	0.55 (0.34, 0.81)	5.92%	0.71%	1.31 (0.87, 1.88)	5.92%	0.71%
Female	1.44 (1.01, 1.98)	6.05%	0.86%	0.83 (0.53, 1.21)	6.27%	0.55%	1.38 (0.89, 1.98)	6.27%	0.55%
**Bipolar disorder**									
Both	0.69 (0.51, 0.95)	6.38%	0.36%	0.87 (0.56, 1.33)	6.83%	−0.06%	1.73 (1.09, 2.65)	6.83%	−0.06%
Male	0.68 (0.50, 0.93)	7.37%	0.41%	0.83 (0.53, 1.25)	7.88%	0.03%	2.01 (1.28, 3.11)	7.88%	0.03%
Female	0.71 (0.52, 0.97)	5.52%	0.32%	0.92 (0.60, 1.41)	5.88%	−0.14%	1.52 (0.95, 2.36)	5.88%	−0.14%
**Anxiety disorders**									
Both	5.80 (4.27, 7.65)	−1.33%	24.82%	4.02 (2.73, 5.71)	−1.11%	24.46%	7.92 (5.40, 10.86)	−1.11%	24.46%
Male	4.41 (3.22, 5.85)	−0.62%	23.94%	2.98 (1.97, 4.26)	−0.34%	23.75%	7.18 (4.94, 9.85)	−0.34%	23.75%
Female	7.18 (5.31, 9.47)	−1.47%	25.42%	5.13 (3.54, 7.20)	−1.31%	24.97%	8.47 (5.80, 11.61)	−1.31%	24.97%
**Eating disorders**									
Both	0.58 (0.36, 0.88)	19.68%	0.21%	0.71 (0.40, 1.11)	19.89%	−0.07%	1.39 (0.83, 2.20)	19.95%	−0.07%
Male	0.40 (0.23, 0.67)	21.99%	1.00%	0.47 (0.24, 0.80)	22.27%	0.85%	1.14 (0.61, 1.89)	22.30%	0.85%
Female	0.76 (0.49, 1.10)	18.97%	−0.14%	0.96 (0.57, 1.45)	19.11%	−0.50%	1.57 (0.97, 2.38)	19.19%	−0.50%
**Anorexia nervosa**									
Both	0.15 (0.09, 0.22)	8.51%	−0.49%	0.18 (0.10, 0.28)	8.51%	−0.77%	0.35 (0.21, 0.54)	8.64%	−0.78%
Male	0.08 (0.05, 0.12)	11.71%	−0.09%	0.10 (0.05, 0.16)	11.62%	−0.12%	0.23 (0.13, 0.37)	11.80%	−0.12%
Female	0.21 (0.13, 0.32)	7.85%	−0.57%	0.27 (0.16, 0.41)	7.90%	−0.93%	0.44 (0.26, 0.69)	7.99%	−0.92%
**Bulimia nervosa**									
Both	0.44 (0.22, 0.75)	24.05%	0.45%	0.53 (0.25, 0.90)	24.38%	0.18%	1.04 (0.50, 1.82)	24.38%	0.18%
Male	0.32 (0.14, 0.58)	24.95%	1.28%	0.38 (0.16, 0.69)	25.34%	1.10%	0.91 (0.40, 1.65)	25.34%	1.10%
Female	0.55 (0.29, 0.91)	23.95%	0.02%	0.69 (0.35, 1.15)	24.25%	−0.33%	1.13 (0.58, 1.92)	24.25%	−0.33%
**Autism spectrum disorders**									
Both	0.84 (0.71, 0.98)	2.95%	0.30%	0.91 (0.63, 1.31)	3.26%	0.05%	1.82 (1.16, 2.73)	3.26%	0.05%
Male	1.14 (0.97, 1.33)	1.39%	0.08%	1.20 (0.82, 1.68)	1.72%	−0.11%	2.91 (1.86, 4.29)	1.72%	−0.11%
Female	0.54 (0.46, 0.65)	5.51%	0.63%	0.61 (0.41, 0.88)	5.77%	0.29%	1.01 (0.65, 1.54)	5.77%	0.29%
**ADHD**									
Both	1.62 (1.12, 2.26)	−16.10%	−0.79%	0.11 (0.06, 0.17)	−16.03%	−0.92%	0.22 (0.13, 0.34)	−16.03%	−0.92%
Male	2.32 (1.62, 3.23)	−16.54%	−0.62%	0.16 (0.09, 0.24)	−16.46%	−0.69%	0.37 (0.22, 0.57)	−16.46%	−0.69%
Female	0.93 (0.62, 1.29)	−15.90%	−1.38%	0.07 (0.04, 0.10)	−15.82%	−1.68%	0.11 (0.06, 0.17)	−15.82%	−1.68%
**Conduct disorder**									
Both	0.11 (0.06, 0.18)	2.70%	0.27%	0.08 (0.04, 0.13)	2.98%	0.08%	0.15 (0.08, 0.26)	2.98%	0.08%
Male	0.18 (0.11, 0.27)	1.81%	0.17%	0.12 (0.06, 0.19)	2.05%	0.05%	0.28 (0.16, 0.46)	2.05%	0.05%
Female	0.05 (0.02, 0.09)	4.04%	0.35%	0.03 (0.01, 0.06)	4.25%	0.18%	0.06 (0.02, 0.10)	4.25%	0.18%
**IDID**									
Both	1.52 (0.85, 2.18)	−6.69%	0.97%	0.37 (0.18, 0.59)	−0.82%	1.29%	0.72 (0.39, 1.08)	−0.82%	1.29%
Male	1.49 (0.79, 2.20)	−10.46%	0.66%	0.35 (0.16, 0.59)	−5.49%	1.03%	0.84 (0.43, 1.30)	−5.49%	1.03%
Female	1.54 (0.92, 2.15)	−2.71%	1.28%	0.38 (0.20, 0.59)	4.28%	1.56%	0.63 (0.35, 0.92)	4.28%	1.56%
**Other mental disorders**									
Both	1.04 (0.68, 1.48)	0.42%	0.11%	0.46 (0.28, 0.68)	0.66%	−0.22%	0.90 (0.56, 1.30)	0.66%	−0.22%
Male	1.29 (0.85, 1.83)	0.11%	0.08%	0.55 (0.34, 0.83)	0.37%	−0.19%	1.32 (0.85, 1.90)	0.37%	−0.19%
Female	0.79 (0.51, 1.12)	0.50%	0.09%	0.36 (0.21, 0.54)	0.69%	−0.35%	0.59 (0.35, 0.87)	0.69%	−0.35%
**Substance use disorders**									
Both	3.36 (2.74, 4.10)	−19.70%	−2.00%	3.16 (2.61, 3.83)	−14.37%	−0.10%	4.89 (3.76, 6.26)	−14.55%	−0.89%
Male	4.48 (3.65, 5.51)	−19.75%	−2.93%	4.05 (3.33, 4.90)	−14.19%	−0.40%	7.30 (5.70, 9.34)	−15.80%	−1.31%
Female	2.24 (1.85, 2.75)	−20.17%	−0.21%	2.21 (1.72, 2.70)	−15.29%	0.40%	3.11 (2.29, 4.03)	−12.77%	−0.23%
**Alcohol use disorders**									
Both	1.51 (1.02, 2.15)	−25.29%	−2.71%	1.00 (0.70, 1.48)	−26.65%	−2.93%	1.73 (1.13, 2.61)	−25.24%	−2.90%
Male	2.21 (1.52, 3.10)	−27.46%	−2.88%	1.46 (1.03, 2.12)	−28.19%	−3.05%	3.00 (1.99, 4.45)	−27.35%	−2.99%
Female	0.80 (0.52, 1.20)	−19.74%	−2.44%	0.51 (0.33, 0.79)	−22.73%	−2.80%	0.79 (0.47, 1.22)	−19.78%	−2.83%
**Drug use disorders**									
Both	1.90 (1.56, 2.38)	−14.82%	−1.40%	2.16 (1.71, 2.59)	−6.75%	1.29%	3.16 (2.32, 4.13)	−6.98%	0.25%
Male	2.34 (1.92, 2.94)	−11.29%	−2.94%	2.59 (2.08, 3.05)	−2.98%	1.17%	4.30 (3.22, 5.48)	−4.82%	−0.10%
Female	1.46 (1.20, 1.85)	−20.37%	1.09%	1.70 (1.29, 2.10)	−12.63%	1.42%	2.32 (1.64, 3.10)	−9.97%	0.69%

95% UI, 95% uncertainty interval.

*YLDs* years lived with disability, *DALYs* disability-adjusted life years, *ADHD* attention-deficit/hyperactivity disorder, *IDID* idiopathic developmental intellectual disability.

All prevalence estimates in this study refer to point prevalence.

As detailed in Table 3, the geographic distribution of mental disorders in 2021 revealed distinct global patterns, with the burden concentrated in high-income regions such as Australasia, North America, and Western Europe. Depressive disorders were most prominent in high-income North America, with prevalence rates of 6114.8 (95% UI 4845.4–7671.5) and 8842.2 (95% UI 6764.4–11606.8) per 100,000 in adolescents and young adults, respectively. Anxiety disorders prevalence peaked in Western Europe among adolescents (9429.5 per 100,000) and in Tropical Latin America among young adults (11194.3 per 100,000). ADHD and bipolar disorder were most prevalent in Latin America and the Caribbean, while eating disorders and ASD were most common in high-income regions. In contrast, conduct disorder and schizophrenia showed minimal geographic variation.

**Table 3:** Prevalence per 100,000 people by mental health disorder and region in 2021.

Group	Region	Mental disorders	Autism spectrum disorders	IDID	Schizophrenia	ADHD	Depressive disorders	Bipolar disorder	Anxiety disorders	Eating disorders	Conduct disorder	Other mental disorders
**Age group 10–19**	**Global**	**14,324.6** (**12,499.9–16,290.1)**	**836.1** (**705.2–981.5)**	**1621.8** (**917.7–2300.4)**	**38.7** (**24.5–58.5)**	**2444.5** (**1677.4–3474.1)**	**2287.5** (**1664.3–2970.6)**	**292.1** (**209.2–407.3)**	**4658.3** (**3502.5–6122.2)**	**259.0** (**169.6–391.1)**	**2561.5** (**1797.9–3361.9)**	**228.8** (**142.0–330.3)**
	**Central Europe, Eastern Europe, and Central Asia**	**13,032.8** (**11,326.8–14,825.0)**	**987.0** (**828.2–1164.2)**	**550.9** (**147.4–934.1)**	**27.2** (**15.6–44.3)**	**2315.0** (**1597.7–3320.2)**	**1943.4** (**1401.8–2574.3)**	**250.7** (**169.4–364.6)**	**4368.9** (**3251.8–5796.8)**	**255.6** (**171.0–387.9)**	**2834.1** (**2005.4–3705.1)**	**219.8** (**135.7–316.7)**
	Central Asia	11,769.3 (10,226.7–13,427.1)	950.2 (798.3–1116.9)	745.5 (279.2–1187.0)	27.4 (14.6–50.3)	2270.5 (1539.7–3261.6)	2025.2 (1405.2–2727.5)	251.0 (153.2–403.3)	2911.7 (1935.8–4174.9)	215.5 (142.7–326.5)	2729.6 (1902.4–3605.3)	246.5 (154.3–345.2)
	Central Europe	13,092.1 (11,193.4–14,942.0)	1018.2 (854.6–1201.9)	394.5 (60.2–726.7)	27.4 (15.2–46.6)	2291.6 (1575.1–3257.4)	1700.8 (1231.7–2258.6)	282.4 (188.1–418.1)	4803.2 (3435.8–6546.3)	282.8 (186.3–429.8)	2768.2 (1948.0–3624.1)	236.7 (146.8–338.8)
	Eastern Europe	13,831.0 (12,132.9–15,734.1)	994.7 (835.0–1182.1)	505.2 (123.0–884.7)	27.0 (16.9–39.6)	2356.5 (1586.0–3427.6)	2016.9 (1484.3–2677.7)	233.8 (167.0–326.6)	5097.7 (3937.0–6619.2)	267.7 (177.8–408.6)	267.7 (177.8–408.6)	193.3 (116.7–280.4)
	**High Income**	**19,750.2** (**17,342.0–22,519.6)**	**1144.5** (**964.1–133.09.0)**	**417.9** (**91.4–769.1)**	**37.2** (**24.2–56.2)**	**3933.6** (**2702.1–5528.2)**	**4442.4** (**3387.3–5584.2)**	**592.5** (**475.3–740.5)**	**7115.5** (**5308.5–9263.3)**	**645.8** (**434.7–961.6)**	**2790.1** (**1991.2–3631.9)**	**348.0** (**236.8–471.1)**
	Australasia	22,672.9 (19,915.9–25,815.8)	1247.6 (1041.3–1490.7)	303.9 (52.5–609.0)	60.8 (44.8–86.0)	7256.7 (5283.2–9589.3)	4159.2 (2933.9–5810.3)	998.8 (746.1–1292.0)	6682.1 (4615.7–9575.8)	1078.5 (743.5–1559.3)	2815.5 (2026.7–3627.8)	404.1 (304.9–517.6)
	High-Income Asia Pacific	14,168.9 (12,448.5–16,126.4)	1635.0 (1378.5–1921.5)	85.1 (3.2–278.0)	31.1 (19.1–47.5)	3056.6 (2107.4–4416.5)	2022.5 (1488.5–2583.8)	312.2 (224.0–426.7)	4355.0 (3199.9–5817.2)	587.3 (397.4–881.1)	2691.0 (1927.7–3488.3)	275.4 (178.0–381.5)
	High-Income North America	20,640.7 (18,235.5–23,201.3)	1162.5 (980.2–1370.9)	501.5 (88.5–912.7)	41.5 (28.1–58.8)	4819.4 (3216.6–6844.6)	6114.8 (4845.4–7671.4)	644.9 (577.6–712.1)	5648.7 (4398.2–7018.8)	631.5 (414.6–966.5)	2573.3 (1815.9–3423.0)	417.3 (287.4–565.0)
	Southern Latin America	18,442.7 (15,628.4–21,740.0)	1129.8 (947.6–1329.8)	474.7 (80.5–865.1)	35.4 (18.8–63.7)	2968.7 (2016.3–4249.2)	3433.9 (2440.3–4573.4)	536.5 (344.7–817.9)	7767.8 (5006.4–11,189.6)	489.7 (321.9–748.2)	2723.6 (1930.5–3553.6)	328.5 (213.7–452.1)
	Western Europe	20,853.7 (17,952.2–24,196.9)	950.0,93.0 (800.0–1115.5)	447.6 (127.6–786.9)	33.7 (20.6–54.5)	3300.0 (2239.9–4679.7)	3859.9 (2709.7–5252.9)	617.8 (443.7–849.1)	9429.5 (6785.0–12,717.0)	679.3 (460.0–1048.2)	3051.9 (2216.6–3899.8)	304.0 (198.2–418.2)
	**Latin America and Caribbean**	**16,900.2** (**14,624.0–19,411.4)**	**746.4** (**627.7–882.6)**	**405.9** (**90.3–707.0)**	**38.2** (**22.9–58.9)**	**4198.0** (**2892.9–5969.9)**	**2022.0,24.0** (**1465.6–2641.8)**	**699.6** (**502.8–952.0,75.0)**	**6748.6 (5079.2–8899.4)**	**320.7** (**208.3–490.4)**	**2692.0** (**1887.8–3528.5)**	**226.1** (**139.5–327.0)**
	Andean Latin America	17,994.5 (15,113.2–21,345.6)	734.8 (614.5–870.4)	441.4 (115.3–745.4)	37.7 (20.4–65.8)	4801.6 (3251.2–6923.7)	191.04.3 (1311.0–2685.5)	596.4 (374.8–925.9)	7563.8 (5040.8–10,805.4)	342.7 (218.3–534.0)	2628.2 (1839.8–3408.1)	259.1 (162.2–362.8)
	Central Latin America	14,874.4 (12,842.2–17,052.8)	815.0 (686.3–962.2)	363.4 (63.2–658.2)	38.2 (22.6–60.2)	3216.1 (2203.8–4585.8)	1932.8 (1408.5–2527.5)	606.2 (436.7–832.8)	5596.1 (4108.6–7560.3)	312.5 (200.8–483.6)	2694.4 (1893.9–3522.5)	229.4 (141.7–330.9)
	Tropical Latin America	18,925.4 (16,314.0–21,729.2)	663.4 (556.2–787.9)	415.3 (104.2–708.3)	38.2 (24.6–54.8)	4726.2 (3253.8–6788.3)	2136.5 (1569.8–2754.6)	882.0 (660.2–1137.7)	8262.0 (6348.5–10,600.0)	334.8 (221.0–507.2)	2735.4 (1931.5–3598.7)	201.9 (121.1–293.5)
	Caribbean	18,202.8 (15,405.3–21,397.7)	725.8 (609.9–862.2)	558.1 (160.1–939.0)	38.4 (20.9–65.4)	6683.1 (4597.7–9327.9)	2208.9 (1538.3–3045.6)	606.7 (377.7–943.5)	5632.9 (3693.7–8056.5)	274.2 (176.0–421.3)	2586.2 (1800.7–3405.4)	261.6 (163.8–366.3)
	North Africa and Middle East	19,026.0 (16,192.6–22,199.3)	810.9 (681.6–954.9)	1905.8 (1073.5–2688.7)	35.8 (19.8–61.7)	2612.3 (1798.5–3702.8)	3665.0 (2537.5–4965.6)	503.0 (329.4–763.2)	8072.5 (5684.1–11,228.4)	303.8 (197.1–465.0)	2436.7 (1707.5–3183.3)	246.7 (153.6–347.1)
	**South Asia**	**13,101.9** (**11,335.0–14,865.2)**	**739.7** (**623.1–864.1)**	**3,989.3** (**2,576.4–5,375.2)**	**39.3** (**24.9–57.0)**	**1,271.3** (**840.4–1,827.7)**	**1,958.8** (**1,451.8–2,559.1)**	**161.6** (**113.9–229.4)**	**2,780.6** (**2,136.3–3,584.6)**	**213.2** (**136.9–327.2)**	**2,483.5** (**1,689.0–3,288.0)**	**205.6** (**124.5–298.1)**
	**Southeast Asia, East Asia, Oceania**	**13,092.0 (11,272.2–15,014.6)**	**710.2** (**595.2–836.6)**	**577.2** (**233.2–924.9)**	**49.7** (**33.7–70.5)**	**3,810.4** (**2,630.7–5,347.2)**	**1,289.0** (**956.5–1,685.5)**	**124.5** (**87.8–174.1)**	**4,468.9** (**3,352.6–5,839.8)**	**189.2** (**122.5–286.6)**	**2,357.1** (**1,668.4–3,083.3)**	**205.8** (**126.1–297.6)**
	East Asia	13,571.1 (11,548.7–15,707.5)	698.4 (583.8–830.1)	389.2 (117.7–665.3)	50.5 (35.1–69.8)	5,037.0 (3,520.2–7,072.8)	913.0 (706.9–1,170.1)	102.0 (72.9–137.7)	4,523.5 (3,383.0–5,870.5)	192.0 (123.3–294.9)	2,194.9 (1,525.7–2,890.5)	193.3 (115.9–281.0)
	Southeast Asia	12,397.2 (10,812.2–14,070.0)	726.9 (610.2–859.1)	844.0 (383.4–1,289.4)	48.7 (30.4–74.3)	2,062.0 (1,402.4–2,925.1)	1,824.7 (1,314.3–2,427.2)	157.1 (105.5–229.9)	4,376.8 (3,290.0–5,769.6)	186.2 (121.9–280.2)	2590.2 (1834.2–3410.5)	222.8 (137.4–322.2)
	Oceania	13,070.2 (10,845.1–15,514.8)	724.3 (609.4–867.9)	827.0 (332.1–1310.8)	43.5 (23.5–76.4)	2506.1 (1723.1–3662.4)	1698.6 (1168.1–2356.8)	128.5 (77.6–210.7)	4998.4 (3239.0–7348.4)	145.7 (95.9–221.8)	2450.2 (1721.6–3212.2)	252.2 (157.9–353.2)
	**Sub-saharan Africa**	**12,084.4** (**10,412.2–13,794.0)**	**966.8** (**814.1–1134.5)**	**677.1** (**227.1–1130.6)**	**30.4** (**17.6–49.9)**	**1190.0** (**798.2–1741.3)**	**2357.0** (**1683.3–3141.8)**	**276.6** (**184.8–412.4)**	**4081.0** (**2959.4–5537.6)**	**173.2** (**113.0–261.0)**	**2728.5** (**1925.7–3575.8)**	**223.6** (**138.3–321.1)**
	Central Sub-saharan Africa	13,034.0 (10,992.1–15,198.2)	964.1 (807.2–1140.5)	945.6 (386.4–1464.3)	29.1 (15.6–53.1)	1158.7 (776.5–1681.4)	3104.8 (2026.1–4446.5)	274.6 (169.2–438.8)	4212.8 (2812.8–6168.4)	153.8 (101.7–229.3)	2673.5 (1854.0–3528.1)	245.0 (153.4–343.1)
	Eastern Sub-saharan Africa	12,499.5 (10,784.5–14,230.2)	973.2 (819.9–1140.5)	798.9 (298.4–1300.4)	29.9 (17.3–49.3)	1165.5 (782.6–1703.8)	2624.8 (1871.3–3464.1)	314.4 (212.6–465.9)	4126.6 (2991.1–5620.6)	157.6 (103.1–237.3)	2749.8 (1930.9–3563.4)	222.07.2 (140.6–325.9)
	Southern Sub-saharan Africa	13,103.2 (11,349.4–14,832.4)	980.4 (826.4–1160.3)	396.7 (86.4–718.7)	30.5 (18.1–46.2)	1171.3 (788.6–1713.3)	2521.3 (1851.8–3213.4)	268.0 (184.2–381.8)	5191.4 (3866.4–6750.2)	232.7 (150.4–359.1)	2802.9 (1972.7–3670.1)	212.9 (131.2–307.3)
	Western Sub-saharan Africa	11,319.0 (9709.7–12,893.1)	960.0 (809.1–1129.1)	530.0 (136.8–940.5)	31.1 (18.4–50.3)	1223.0 (816.7–1801.3)	1889.2 (1353.6–2487.2)	244.7 (165.7–359.8)	3860.6 (2789.3–5282.9)	185.0 (120.5–279.9)	2715.3 (1892.4–3552.0)	215.9 (133.3–310.9)
**Age group 20-24**	**Global**	**15,758.3** (**13,651.3–18,034.9)**	**820.5** (**691.9–962.2)**	**1481.2** (**829.4–2125.7)**	**250.5** (**162.2 –367.1)**	**1587.6 (1097.1–2212.3)**	**4683.1** (**3523.6–6459.5)**	**677.3** (**495.1–924.5)**	**5664.5** (**4179.3–7486.1)**	**569.1 (353.5–859.2)**	**109.9** (**61.5–173.8)**	**1019.8** (**667.6–1444.6)**
	**Central Europe, Eastern Europe, and Central Asia**	**14,229.7** (**12,259.1–16,332.9)**	**976.2** (**818.7–1150.6)**	**502.8** (**127.0–868.9)**	**184.3 (115.0–281.7)**	**1568.6** (**1076.5–2196.2)**	**4424.0** (**3272.3–6012.9)**	**648.8 (455.4–913.8)**	**5109.6** (**3662.7–6927.2)**	**531.0** (**334.2–815.8)**	**120.2** (**65.4–186.4)**	**1011.0** (**656.1–1437.2)**
	Central Asia	12,673.1 (10,854.9–14,778.6)	936.2 (785.7–1102.6)	680.0 (238.1–1100.0)	187.3 (107.9–306.7)	1512.1 (1040.0–2144.1)	4387.5 (3148.2–6167.6)	638.0 (401.1–962.6)	3316.4 (2254.1–4929.7)	448.5 (280.7–689.9)	119.2 (64.7–187.6)	1134.0 (735.1–1559.9)
	Central Europe	14,001.5 (11,901.2–16,496.1)	1009.8 (847.4–1190.1)	370.8 (52.7–690.4)	189.7 (114.6–298.2)	1554.3 (1069.3–2168.9)	3672.5 (2702.6–5023.0)	690.4 (484.4–981.0)	5590.0 (3939.4–7834.8)	567.2 (355.7–865.0)	120.3 (66.1–186.5)	1050.6 (684.7–1486.2)
	Eastern Europe	15,464.7 (13,399.4–17,712.7)	984.0 (825.9–1170.3)	458.5 (100.3–819.6)	178.8 (115.4–261.4)	1617.2 (1105.5–2243.8)	4907.4 (3593.5–6740.8)	631.2 (462.8–839.0)	6079.5 (4505.1–8047.4)	567.0 (354.8–867.9)	121.0 (64.3–193.2)	900.3 (575.8–1279.2)
	**High Income**	**21,594.5** (**18,802.5–24,477.7)**	**1143.1** (**962.4–1337.5)**	**377.4** (**76.0–707.2)**	**266.7** (**182.6–378.9)**	**2305.2 (1562.0–3225.2)**	**7038.4** (**5287.1–9535.2)**	**1108.2** (**894.1–1389.6)**	**8475.7** (**6297.0–11,104.1)**	**1348.1** (**904.4–1903.3)**	**142.7** (**81.3–222.3)**	**1472.7** (**1024.5–1978.1)**
	Australasia	27,261.9 (23,777.1–31,589.3)	1241.2 (1037.0–1483.1)	280.3 (45.1–570.6)	486.4 (408.3–562.9)	4659.0 (3372.6–6282.5)	8002.4 (5826.3–11,210.1)	1945.2 (1500.3–2469.8)	9357.6 (6195.6–13,293.4)	2899.6 (2093.3–3925.6)	152.0 (87.9–241.0)	1757.0 (1350.7–2223.5)
	High-Income Asia Pacific	14,971.0 (13,101.9–16,876.0)	1621.0 (1364.7–1901.2)	70.4 (2.4–248.2)	227.9 (143.4–341.6)	1895.9 (1267.7–2714.6)	4350.7 (3352.7–5674.1)	684.4 (501.7–913.0)	4518.4 (3272.1–6079.5)	1257.5 (832.9–1792.7)	131.1 (74.0–205.1)	1191.2 (797.9–1633.2)
	High-Income North America	24,500.2 (21,511.8–27,779.2)	1152.8 (970.2–1357.4)	465.1 (74.7–855.8)	329.5 (229.1–448.6)	2941.2 (1979.3–4148.1)	8842.2 (6764.4–11,606.8)	1077.9 (976.8–1182.0)	9148.8 (6625.2–11,959.9)	1317.4 (860.7–1893.1)	125.2 (69.9–199.1)	1742.2 (1221.0–2336.3)
	Southern Latin America	20,231.8 (16,821.2–23,762.0)	1117.5 (936.8–1314.5)	438.1 (69.2–807.8)	239.7 (139.1–393.3)	1750.6 (1196.0–2466.6)	6095.6 (4434.4–8190.9)	1090.8 (717.6–1613.5)	8639.6 (5621.3–12,420.6)	1065.9 (675.6–1556.0)	140.5 (79.8–224.6)	1402.8 (937.3–1922.4)
	Western Europe	21,162.7 (18,107.7–24,451.0)	945.0 (794.0–1108.7)	405.6 (106.9–726.4)	208.5 (136.8–315.5)	1771.3 (1198.1–2495.3)	6440.8 (4727.3–9122.7)	1239.8 (911.6–1677.7)	9251.0 (6769.9–12,265.3)	1349.7 (920.6–1900.9)	164.2 (94.4–252.5)	1310.1 (873.5–1791.9)
	**Latin America and Caribbean**	**19,601.6** (**16,769.9–22,682.8)**	**730.9** (**613.7–865.3)**	**364.7 (74.6–648.1)**	**215.6** (**136.0–318.5)**	**2884.9** (**2016.7–4028.5)**	**4941.8** (**3680.7–6842.6)**	**1516.7** (**1123.7–2007.7)**	**8692.6** (**6383.4–11,651.8)**	**794.0 (485.6–1186.8)**	**116.4** (**65.7–184.5)**	**981.8** (**637.5–1399.4)**
	Andean Latin America	20,407.1 (16,964.0–24,745.0)	723.5 (604.8–858.4)	401.9 (94.8–690.5)	215.0 (124.8–335.5)	3288.0 (2241.7–4605.6)	4210.5 (2997.6–5960.6)	1326.3 (866.9–1969.9)	9741.9 (6399.7–14,496.3)	964.5 (587.0–1433.4)	117.8 (62.2–189.5)	1134.3 (735.3–1560.5)
	Central Latin America	16,800.2 (14,472.8–19,512.6)	800.9 (673.0–946.0)	327.4 (52.2–606.4)	216.2 (136.1–320.6)	2103.1 (1459.5–2948.4)	4666.9 (3447.2–6398.5)	1324.5 (969.8–1764.5)	6667.7 (4787.5–9184.6)	775.2 (470.9–1173.3)	115.7 (66.0–184.2)	997.0 (647.3–1420.2)
	Tropical Latin America	22,747.4 (19,478.4–26,118.1)	652.6 (546.0–775.8)	372.3 (83.5–646.6)	217.6 (141.9–315.9)	3312.9 (2260.7–4636.6)	5517.4 (4182.3–7501.9)	1847.6 (1419.3–2317.6)	11,194.3 (8300.8–14,478.3)	783.4 (480.2–1162.9)	116.4 (64.9–185.2)	882.5 (562.9–1250.7)
	Caribbean	19,279.0 (16,232.6–22,837.7)	717.7 (602.5–853.2)	482.2 (125.1–827.9)	203.9 (121.3–320.4)	4646.5 (3241.2–6547.8)	4897.9 (3462.4–7152.4)	1329.3 (859.7–1969.3)	6663.6 (4419.4–9742.0)	686.9 (429.3–1037.0)	117.8 (62.8–189.6)	1130.2 (732.7–1554.6)
	North Africa and Middle East	20,384.5 (17,223.0–23,968.7)	802.5 (675.8–947.5)	1673.0 (916.4–2389.5)	231.7 (141.4–367.2)	1763.7 (1244.1–2436.7)	6378.5 (4519.2–9255.2)	1129.7 (765.8–1655.5)	8367.4 (5888.1–11,405.0)	616.4 (377.8–939.5)	111.1 (62.6–177.1)	1107.9 (717.9–1536.5)
	**South Asia**	**14,928.8 (12,828.8–17,313.2)**	**726.2** (**611.8–848.5)**	**3590.7** (**2306.0–4883.7)**	**255.1** (**166.8–372.2)**	**832.2** (**555.4–1177.7)**	**4465.5 (3368.5–6069.8)**	**417.5** (**303.9–564.7)**	**4139.0** (**3015.7–5462.9)**	**458.1** (**276.2–715.9)**	**101.8** (**56.4–163.2)**	**901.8 (577.1–1281.8)**
	**Southeast Asia, East Asia, Oceania**	**12,212.2** (**10,584.4–13,973.6)**	**701.8** (**587.4–827.5)**	**524.8** (**201.7–853.5)**	**308.5 (205.3–431.2)**	**2320.2** (**1627.6–3249.0)**	**2623.7** (**1960.3–3515.8)**	**306.8 (218.9–416.8)**	**4625.1** (**3457.5–6130.3)**	**385.3** (**230.9–613.9)**	**95.7 (53.2–153.4)**	**931.8** (**598.6–1323.4)**
	East Asia	11,668.1 (10,050.5–13,310.2)	692.0 (577.0–824.2)	351.0 (99.7–608.8)	314.2 (213.9–423.5)	3050.4 (2119.6–4311.1)	2060.7 (1575.0–2686.8)	238.1 (178.7–311.9)	4143.3 (3131.4–5462.9)	392.2 (232.0–630.3)	88.7 (46.0–144.8)	894.4 (571.6–1268.1)
	Southeast Asia	12,920.1 (11,089.6–14,951.7)	715.1 (599.8–846.1)	757.6 (331.4–1175.9)	301.5 (194.9–442.3)	1343.8 (902.2–1891.7)	3357.1 (2453.8–4643.2)	399.6 (276.8–559.5)	5267.6 (3828.6–7030.7)	378.1 (228.8–597.0)	105.1 (60.2–169.9)	978.0 (634.9–1393.9)
	Oceania	13,650.4 (11,412.5–16,425.4)	710.4 (597.9–851.3)	733.3 (275.1–1178.8)	266.8 (154.5–426.9)	1584.6 (1064.0–2259.5)	4071.2 (2848.8–5843.1)	334.9 (207.9–504.9)	5185.2 (3340.0–7845.4)	290.0 (174.7–454.7)	101.1 (55.8–166.3)	1137.9 (737.5–1565.4)
	**Sub-saharan Africa**	**14,329.4 (12,206.3–16,635.2)**	**942.0** (**792.8–1103.4)**	**585.6** (**176.9–1006.3)**	**198.4** (**123.3–308.1)**	**749.5 (491.4–1043.6)**	**5329.6** (**3920.5–7270.4)**	**705.0** (**488.4–999.8)**	**5146.0** (**3699.2–6957.9)**	**379.7** (**231.2–601.3)**	**114.9** (**65.2–181.7)**	**1019.9** (**661.7–1442.7)**
	Central Sub-saharan Africa	16,935.3 (13,995.3–20,575.4)	944.7 (790.0–1118.1)	832.2 (314.2–1309.3)	187.4 (108.3–307.7)	726.6 (478.1–1037.8)	7816.4 (5455.8–11,181.8)	690.3 (435.4–1030.1)	5286.8 (3476.8–7902.6)	330.9 (200.0–522.2)	116.6 (65.2–197.2)	1128.9 (731.9–1552.8)
	Eastern Sub-saharan Africa	15,415.8 (13,008.0–17,996.2)	946.0 (797.1–1107.3)	691.8 (228.7–1157.2)	191.1 (117.0–298.1)	737.3 (488.3–1027.3)	5708.9 (4215.8–7850.1)	785.4 (545.5–1109.5)	5834.2 (4151.3–7973.3)	336.9 (204.3–543.1)	118.8 (65.6–190.1)	1022.9 (663.7–1446.1)
	Southern Sub-saharan Africa	15,716.1 (13,376.4–18,206.1)	963.5 (811.6–1141.1)	337.8 (64.4–637.8)	195.1 (124.7–292.8)	760.4 (502.9–1057.0)	5917.2 (4418.7–7951.9)	684.4 (489.9–936.4)	6238.8 (4619.8–8305.3)	528.1 (321.0–825.8)	117.7 (66.0–192.8)	955.1 (618.9–1358.1)
	Western Sub-saharan Africa	12,382.7 (10,594.4–14,420.2)	934.2 (785.8–1098.5)	456.0 (101.1–841.2)	208.7 (130.9–319.8)	765.4 (502.9–1090.2)	4193.8 (3062.5–5746.7)	637.6 (444.4–899.7)	4301.8 (3121.6–5824.9)	410.4 (250.0–648.6)	110.5 (60.5–177.6)	996.5 (646.8–1415.2)

95% uncertainty intervals are shown in parentheses. Bolding indicates global estimates or GBD super-regions.

*GBD* global burden of diseases, injuries, and risk factors study, *ADHD* attention-deficit/hyperactivity disorder, *IDID* idiopathic developmental intellectual disability.

All prevalence estimates in this study refer to point prevalence.

### YLDs of mental and substance use disorders

**Fig. 1:**
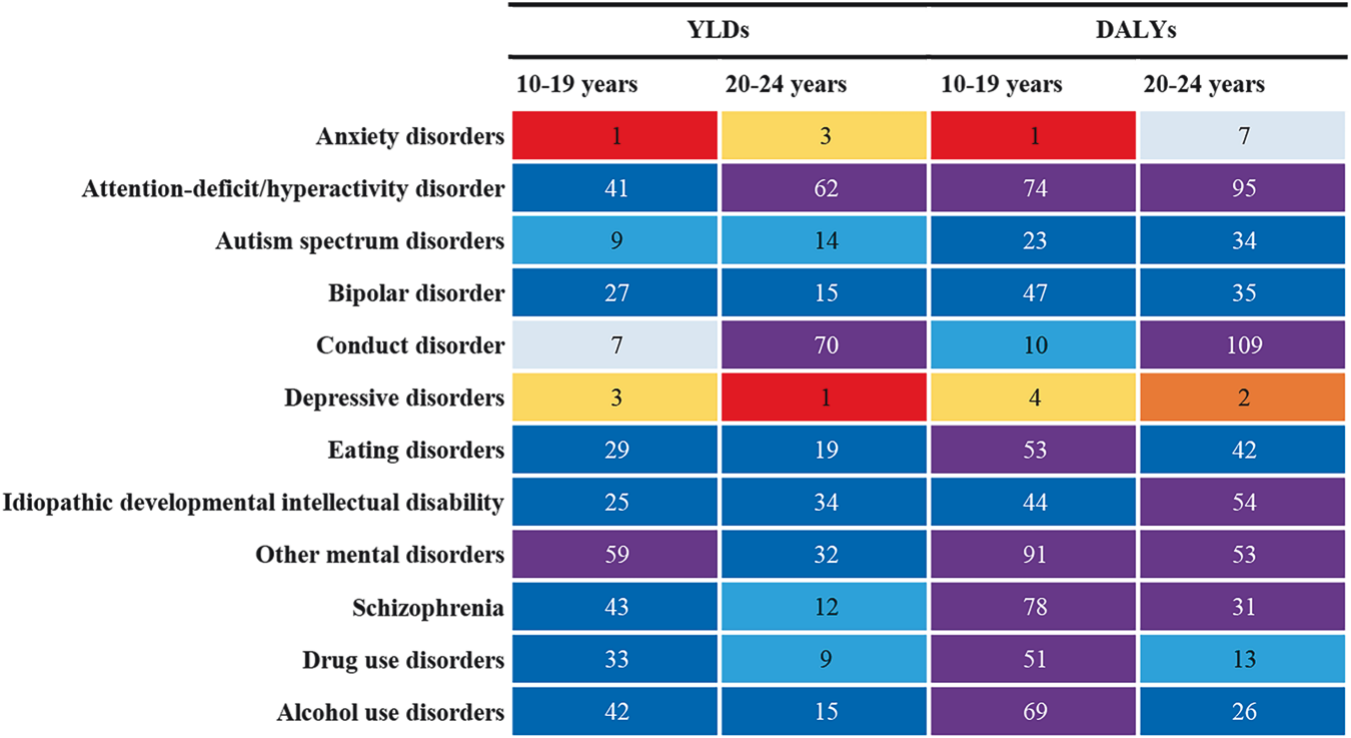
Rankings of DALYs and YLDs rates for mental disorders and substance use disorders in 10–19 age and 20–24 age groups for both sexes combined, in 2021. DALYs, disability adjusted life years; YLDs, years lived with disability.

Mental disorders were a leading cause of YLDs in 2021, accounting for 27.14% (95% UI 22.26–33.44) of total YLDs among10–19-year-olds and 26.62% (95% UI 21.95–32.87) among 20–24-year-olds (Tables 1 and 2). Depressive and anxiety disorders contributed the most to YLDs. As shown in Fig. 1, anxiety disorders ranked first for adolescents (8.92%), followed by depressive disorders (6.76%), conduct disorders (4.82%), and ASD (2.53%). For 20–24 year-olds, depressive disorders took the lead (9.83%), followed by anxiety disorders (7.92%), and schizophrenia (1.94%). Marked age-dependent shifts were observed for specific disorders. For schizophrenia, there was a 4.7-fold higher proportion of YLDs in the 20–24-year-olds (1.94%) than in 10–19-year-olds (0.41%). YLDs from conduct disorders decreased 32-fold (from 4.82% to 0.15%) when comparing the 20–24 years group with the 10–19 years age group. Additionally, DUDs contributed to higher YLDs versus AUDs in both age groups (10–19: 0.64% vs. 0.45%; 20–24: 3.16% vs. 1.73%) (Tables 1 and 2).

### DALYs of mental and substance use disorders

**Fig. 2:**
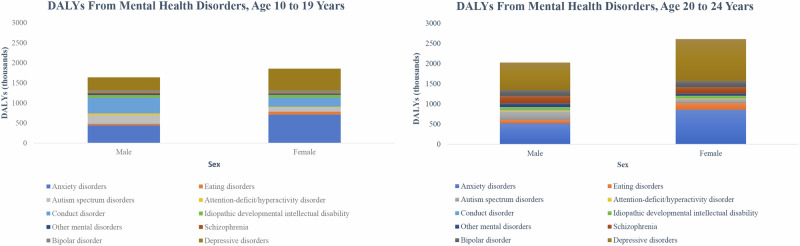
Global DALYs by mental disorders, sex, age group 10–19 years and 20–24 years, in 2021. DALYs, disability adjusted life years.

In 2021, mental disorders were the leading cause of DALYs globally, affecting 15.2% (95% UI 12.0–18.9) of total DALYs in 10–19-year-olds and 13.5% (95% UI 10.7–16.6) in 20–24-year-olds. Figure 2 displays the global distribution of DALYs attributable to specific mental disorders in 2021, stratified by sex and age group, with overall higher DALYs reported in women than men. Among specific mental disorders, anxiety, depressive, and conduct disorders collectively accounted for over half of all DALYs in the 10–19-year age group. In contrast, the DALY burden in the 20–24-year age group was predominantly driven by anxiety and depressive disorders. In addition, anxiety disorders were the leading contributor to DALYs in 10–19-year-olds (5.00%), while depressive disorders ranked first for 20–24-year-olds (4.99%) (Fig. 1 and Tables 1 and 2).

**Fig. 3:**
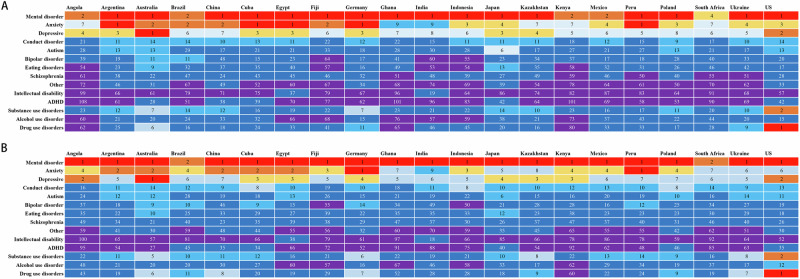
Country rankings of DALY rates for mental and substance use disorders among adolescents and young adults (age 10–24 years) in 2021 and 2050. Rankings of DALY rates for mental and substance use disorders among adolescents and young adults (age 10–24 years) by country in (**A**) 2021 and (**B**) 2050. DALY, disability-adjusted life year; US, United States; ADHD, attention-deficit/hyperactivity disorder; Other, other mental disorders.

SUDs accounted for a larger share of DALYs in young adults (3.16%, 95% UI 2.61–3.83) than in adolescents (0.78%, 95% UI 0.61–0.95). Within SUDs, DUDs drove more DALYs in 20–24-year-olds (2.16%) than AUDs (1.00%), whereas the gap was narrower in 10–19-year-olds (drug use: 0.49%, alcohol use: 0.28%) (Tables 1 and 2). By country, mental disorders topped the list except in Mexico, Brazil, Angola, Kenya, and South Africa. Anxiety or depression was the highest-ranking mental disorder in 2021, and mental disorders will remain the leading cause of DALYs for most countries by 2050, except Brazil and South Africa (Fig. 3).

### Temporal changes of mental and substance use disorders (1990–2021)

#### Trends in prevalence

**Fig. 4:**
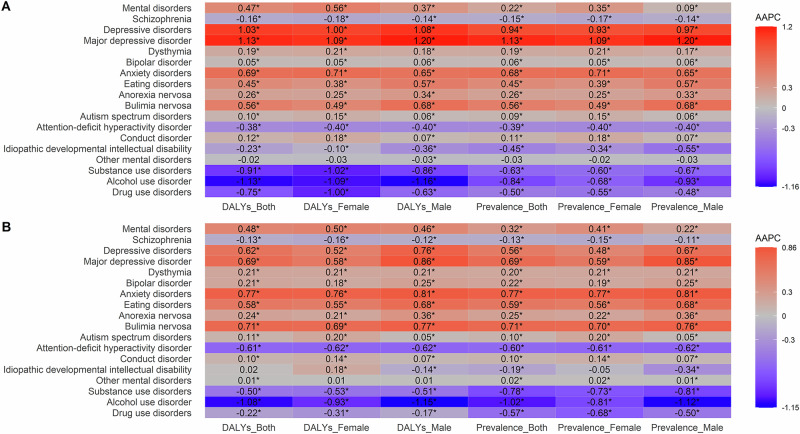
Trends in DALYs and prevalence for mental and substance use disorders among adolescents and young adults (age 10–24 years), 1990–2021. The AAPC in global DALYs and prevalence rates for mental and substance use disorders from 1990 to 2021 in (**A**) adolescents (age 10–19 years) and **B**) young adults (age 20–24 years), stratified by sex. AAPC, average annual percentage change; DALY, disability-adjusted life year. “*” indicates AAPC > 0.

**Fig. 5:**
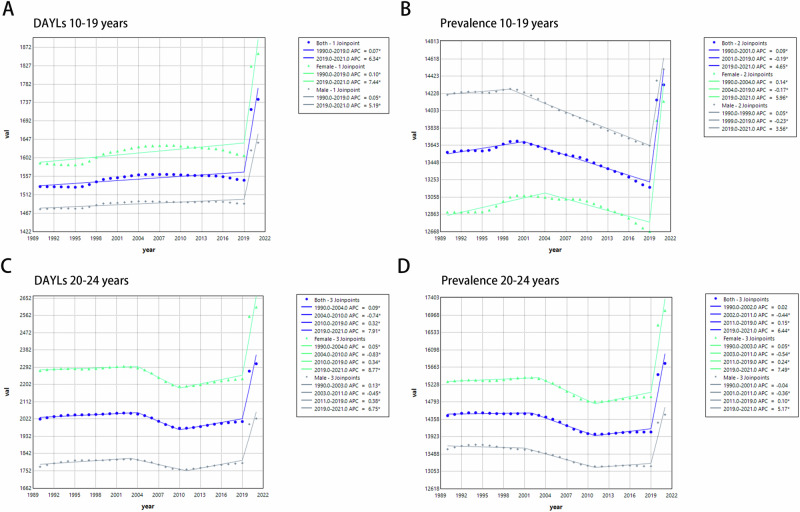
Joinpoint regression analysis results of mental disorders: DALYs and prevalence by two age groups and sex. **A** DALYs in 10–19 years age group; (**B**) prevalence in the 10–19 years age group; (**C**) DALYs in 20–24 years age group; (**D**) prevalence in 20–24 years age group. APC, annual percentage change; DALYs, disability-adjusted life-years. All prevalence estimates in this study refer to point prevalence; * means significance.

Temporal patterns of mental disorders and SUDs prevalence differed by age, sex, and period, as quantified by APC/AAPC from joinpoint regression and period-specific percentage changes (1990–2019 vs. 2019–2021; Tables 1 and 2). From 1990 to 2021, the global prevalence of mental disorders exhibited a significant overall increase (AAPC > 0, P < 0.05), a trend that was more pronounced in females across both age groups (Figs. 4 and 5). Among adolescents of both sexes, mental disorder prevalence declined by 2.92% from 1990–2019 but increased by 8.79% during 2019–2021(Table 1). Similarly, 20–24-year-olds saw a 2.83% prevalence decrease (1990–2019) followed by a sharp 12.25% rise (2019–2021) (Table 2). This substantial increase during 2019–2021 ultimately resulted in a positive AAPC (AAPC > 0, P < 0.05) for the entire 1990–2021 period (Fig. 5**)**. This recent spike largely stemmed from substantial increases in depressive and anxiety disorders. Both conditions remained relatively stable among both age groups from 1990 to 2019, then rose sharply post-2019 (Figures S1 and S2). The change in depressive disorders was mainly driven by a surge in MDD (Figures S3 and S4), while other specific mental disorders showed no such significant shifts during 2019–2021 (Figures S5–S14).

**Fig. 6:**
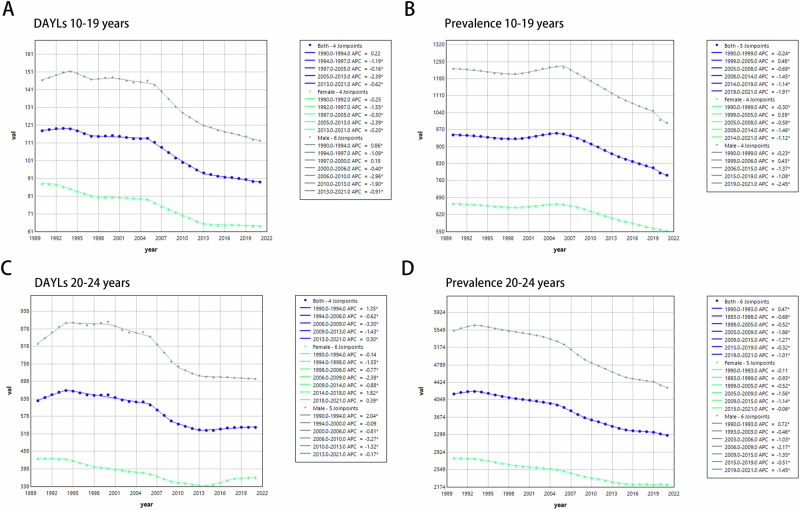
Joinpoint regression analysis results of substance use disorders: DALYs and prevalence by two age groups and sex. **A** DALYs in 10–19 years age group; (**B**) prevalence in the 10–19 years age group; (**C**) DALYs in 20–24 years age group; (**D**) prevalence in 20–24 years age group. APC, annual percentage change; DALYs, disability-adjusted life-years. All prevalence estimates in this study refer to point prevalence; * means significance.

Conversely, SUDs showed a significant overall decrease during the same period, with steeper declines in males across both cohorts (AAPC < 0, P < 0.05) (Figs. 4 and 6). The most dramatic SUD prevalence decline among 10–19-year-olds occurred in 2019–2021: APC was −1.91 (both sexes), −1.12 (females) and −2.45 (males) (P < 0.05). Similarly, 20–24-year-olds had an APC of −1.01 (both sexes) and −1.45 (males) during this period (P < 0.05). This marked reduction stemmed from concurrent declines in both AUDs and DUDs, with DUDs as the primary driver (Figures S15 and S16).

**Fig. 7:**
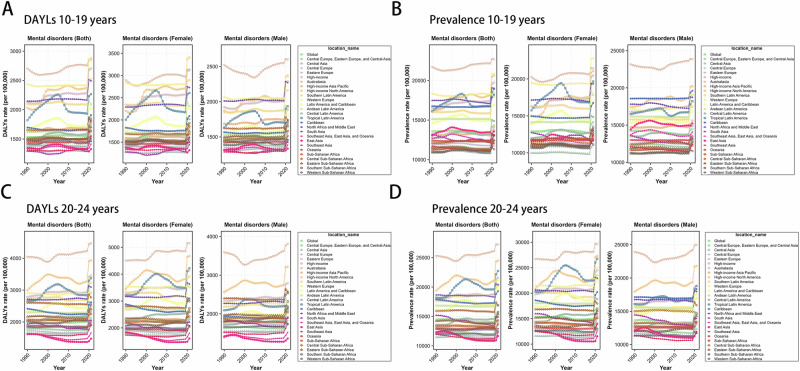
Trends in DALYs and prevalence of mental disorders from 1990–2021, by two age groups, sex, and regions. **A** DALYs in 10–19 years age group; (**B**) prevalence in the 10–19 years age group; (**C**) DALYs in 20–24 years age group; (**D**) prevalence in 20–24 years age group. DALYs, disability-adjusted life-years. All prevalence estimates in this study refer to point prevalence.

**Fig. 8:**
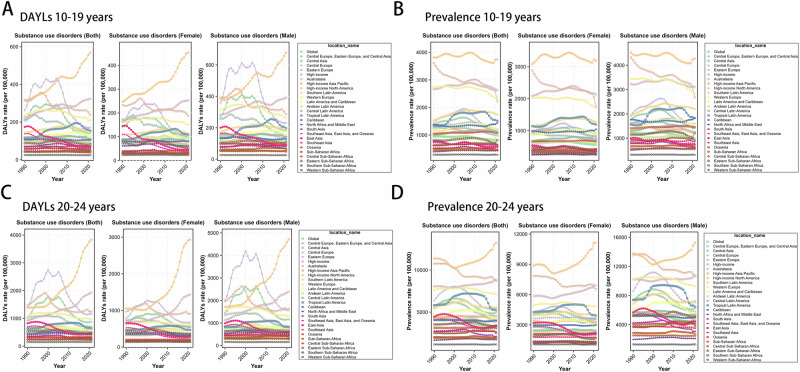
Trends in DALYs and prevalence of substance use disorders from 1990–2021, by two age groups, sex, and regions. **A** DALYs in 10–19 years age group; (**B**) prevalence in the 10–19 years age group; (**C**) DALYs in 20–24 years age group; (**D**) prevalence in 20–24 years age group. DALYs, disability-adjusted life-years. All prevalence estimates in this study refer to point prevalence.

Figures 7 and 8 present temporal trends in the prevalence of mental disorders and SUDs from 1990 to 2021 across global regions, stratified by age and sex. Consistent with the global trend, 2019 marked a critical turning point for mental disorders across all regions and age groups, with prevalence rising sharply between 2019 and 2021, particularly for depressive and anxiety disorders, but no significant changes for other specific mental disorders (Fig. 7**;** Figures S17-S30). Australia consistently had the highest prevalence of mental disorders across both age and sex groups from 1990 to 2021. Among adolescents, rates were higher in males than females, whereas the opposite pattern was observed in young adults (Fig. 7). For depressive disorders, high-income North America and Australia ranked among the most prevalent for adolescents. Among young adults, Central sub-Saharan Africa, high-income North America, and Australia consistently showed higher prevalence rates than other regions, with Central sub-Saharan Africa recording the highest rates in males aged 20–24 throughout 1990–2021 (Figure S17). For anxiety disorders, Western Europe, Tropical Latin America, North Africa and Middle East, and Australia showed the highest prevalence among 10–19-year-olds. Females in Western Europe and males in North Africa and the Middle East showed the highest sex-specific rates (Figure S19). Among 20–24-year-olds, the highest prevalence was observed in Tropical Latin America and Australia (Figure S19). Distinct regional signatures were also observed for other disorders: Australia had markedly higher eating disorder prevalence than any other region across both age groups (Figure S23); high-income Asia Pacific recorded the highest ASD prevalence (Figure S26); Western Europe showed elevated conduct disorder prevalence (Figure S28); and South Asia exhibited the highest IDID prevalence despite an overall declining trend (Figure S29).

High-income North America recorded the highest SUD prevalence for most of the period from 1990 to 2021, with males consistently more affected than females (Fig. 8). Prevalence among adolescents remained relatively stable, whereas young adults experienced a notable upward trend. Australia also exhibited relatively high SUD prevalence, with both age groups declining from 1990 to 2021. DUD trends in these two regions aligned with overall SUDs across both age groups (Figure S31), while AUDs exhibited distinct patterns, with high-income North America exhibiting a long-term decline in both adolescents and young adults between 1990 and 2021 (Figure S32).

### Trends in DALYs

Global DALYs attributable to mental disorders closely mirrored prevalence patterns, whereas SUD-related DALYs showed distinct sex-specific trends. Joinpoint regression identified a statistically significant overall increase in mental disorder-related DALYs globally (1990–2021), with females experiencing steeper rises than males in both age groups (Figs. 4 and 5). This increase was primarily driven by a sharp surge in 2019–2021. From 1990 to 2019, DALY rates changed modestly (1.01% for ages 10–19 years and −0.65% for ages 20–24 years), but surged markedly in 2019–2021 (12.76% for ages 10–19 and 15.01% for ages 20–24 years)(Tables 1 and 2). This pattern aligns with sharp increases in depressive and anxiety disorders during the same period (Figures S1-S3).

Among individuals aged 10–19 and 20–24 years, males consistently carried a higher global SUD burden (measured by DALY rates) than females throughout 1990–2021 (Fig. 6). Although global SUD-related DALYs declined significantly during this period, the magnitude of decline differed by sex. Females experienced more pronounced reductions than males across both age groups (AAPC < 0, P < 0.05; Fig. 4). For specific SUD categories, global DALYs attributable to both AUDs and DUDs decreased significantly from 1990 to 2021 across sexes and age groups. However, AUDs declined more steeply in males, whereas DUDs showed a more pronounced decline in females. Regional analyses revealed that DALY trends for mental disorders and SUDs across 1990–2021 largely tracked corresponding prevalence patterns (Figs. 7 and 8**)**. One exception occurred in high-income North America, where the prevalence of SUDs among adolescents remained relatively stable, while SUD-related DALY increased substantially (Fig. 8).

## Discussion

This study revealed alarming global increases in mental disorders and SUDs among adolescents and young adults. In 2021, over 15% of individuals aged 10–24 years were affected by mental disorders, with depressive and anxiety disorders contributing the largest share of DALYs. Anxiety disorders were highly prevalent across both age groups, whereas conduct disorder and ADHD were more common in adolescents aged 10–19 years, and depressive disorders were the dominant condition among young adults aged 20–24 years. Marked sex differences were observed, with males showing higher rates of ADHD and ASD and females exhibiting higher rates of anorexia nervosa and depressive and anxiety disorders. Between 2019 and 2021, the burden of mental disorders, particularly depression and anxiety, increased significantly, while the prevalence and DALYs associated with SUDs declined.

Mental health disorders were the leading cause of YLDs in both age groups, accounting for over one-quarter of total YLDs. The substantial rise in schizophrenia-related YLDs in young adults aligned with its established peak age of onset (men: late adolescence to early 20 s; women: mid-to-late 20 s) [19], and potential delays in diagnosis and treatment [20]. Conversely, the decline in conduct disorder-related YLDs among 20–24 years-olds may not solely reflect a true reduction in symptom burden. Instead, it could align with established diagnostic conventions. Conduct disorder is typically diagnosed during childhood and adolescence, and when core symptoms persist into adulthood, individuals are often reclassified as having antisocial personality disorder rather than retaining the conduct disorder diagnosis [21]. This diagnostic transition, rather than meaningful clinical improvement, likely accounts for the sharp decline in conduct disorder–specific YLDs observed in young adults. Despite decreased prevalence, SUDs burden intensified in young adults, with its DALY ranking rising from 22nd to 11th as individuals aged, highlighting growing concerns about young adults’ vulnerability to substance-related issues, potentially exacerbated by underlying mental health conditions [22]. This underscores the need for integrated mental health and substance use care to reduce long-term disability and overall disease burden [23].

Beyond age-related patterns, the global distribution of mental health disorders exhibited striking geographic disparities. These disorders consistently ranked as the leading cause of DALYs across most countries, a trend that is projected to continue in 2050. High-income regions report the highest rates, with anxiety disorders most prevalent in Western Europe, depressive disorders in high-income North America, and eating and bipolar disorders in Australasia among adolescents. Similarly, high-income regions continued to exhibit the highest mental health disorder rates in young adults, while Southeast Asia, East Asia, and Oceania have the lowest. These patterns were likely driven by stress, social isolation, and competitive environments [24], highlighting the disproportionate impact on high-income regions [25]. First, academic pressure is substantially higher in many high-income and competitive educational systems, where excessive workload and performance expectations have been strongly linked to depression, anxiety, self-harm, and suicidality [26, 27]. Second, adolescents in these settings face increased exposure to digital environments, including social media-driven comparison, cyberbullying, and online peer competition, which were consistently associated with psychological distress and psychosomatic complaints [28–30]. Third, social isolation, often reinforced by academic pressure, competitive schooling, and digital communication patterns, is a well-documented correlation of adolescent mental-health problems [31]. Finally, higher detection and reporting rates in high-income countries, where mental-health research, screening programs, and clinical services are more accessible, may contribute to higher observed prevalence compared with resource-limited regions, where under-reporting is common [32].

Conversely, the 10–19 age group in Sub-Saharan Africa, particularly Western Sub-Saharan Africa, had the lowest rates. This may reflect underreporting, cultural interpretations of distress, and competing health system priorities [33]. Cultural models of distress in many Sub-Saharan African communities emphasize somatic expressions of emotional distress, spiritual or religious interpretations, and social explanations for mental-health symptoms, which may reduce recognition of mental disorders [34, 35]. Health-system constraints contribute to low reported rates, as many low- and middle-income countries face severe shortages of mental-health professionals [36]. Limited primary-care integration, screening, and diagnostic capacity may also lead to substantial underdetection of adolescent mental-health disorders [37]. These findings underscore the significant influence of socioeconomic factors on mental health. High-income regions face unique challenges, while lower-income areas struggle with underdiagnosis and inadequate resources. Therefore, mental health strategies must be culturally sensitive and age-appropriate [38].

In addition to geographic disparities, the prevalence of certain mental health disorders differed significantly by sex, requiring targeted interventions to address unique needs. Anorexia nervosa was more common in females aged 20–24, potentially due to societal pressures around body image and sexed experiences [39]. Such findings highlight the need for targeted interventions for young women, particularly in addressing body image issues and eating disorders [40]. Conversely, ADHD and ASD were more prevalent among males in both the 10–19 and 20–24 age groups, reflecting both biological factors and potential sex biases in diagnosis [41]. Conduct disorders were also more common among males aged 20–24 compared to females, as reported previously [42]. From 2019 to 2021, DALYs for both sexes increased significantly, primarily due to depression and anxiety disorders. Females consistently reported higher DALYs due to mental disorders in both age groups, with significant increases during the pandemic. Women and girls have been disproportionately exposed to stressors such as caregiving responsibilities, domestic and interpersonal violence, economic insecurity, and social role expectations, all of which intensified during the pandemic [43]. Gendered norms also influence help-seeking behaviors, stigma, and access to care, further shaping observed patterns. Thus, the elevated burden among females likely reflects a combination of biological susceptibilities and gender-specific exposures to stress and adversity rather than inherent vulnerability alone.

For individuals aged 10–19 years, the prevalence of mental disorders increased for both sexes, with a more pronounced resurgence in females compared to males. However, this gender difference should not be interpreted as females being inherently “more prone” to developing anxiety or depressive disorders [44]. Contemporary psychological and developmental research consistently emphasizes that sex differences in mental-health outcomes arise primarily from differential exposure to stressors and sexed social environments, rather than from intrinsic vulnerability alone. Adolescent girls and young women have been disproportionately exposed to several psychosocial risks that intensified during the pandemic, including heightened caregiving burdens in families, increased exposure to domestic and sex-based violence, intensified academic and social pressures, and elevated body-image concerns amplified through digital media use [45, 46]. The pandemic also disrupted support networks, schooling environments, and community structures on which girls rely more heavily for emotional regulation and social buffering [47]. The trends in the prevalence of SUDs also exhibited sex-specific patterns. Among individuals aged 10–19 years, the overall prevalence of SUDs showed a consistent decline throughout the study period, with a more pronounced decrease observed from 2019 to 2021. This decline was significant in both sexes, although the rates were slightly lower in females. In the 20–24-year age group, a similar pattern was observed. This decreased prevalence of SUDs may be attributed to reduced purchasing power and more limited access to drugs during the lockdowns [48].

Our analysis found a significant increase in mental disorders, particularly depression and anxiety, during the pandemic, which aligned with prior evidence of increased mental health issues among adolescents amid COVID-19 [49, 50]. However, the pandemic may have acted as an accelerant rather than the sole driver of the upward trajectory observed from 1990 to 2021. Broader societal and environmental changes over the past decade have also contributed to the escalating burden among adolescents and young adults. Increasing digital media engagement, social comparison, and cyberbullying exposure have been associated with heightened risks of depression, anxiety, and self-harm [51, 52], while declines in sleep quality and physical activity have further impaired emotional regulation [53, 54]. In some regions, elevated exposure to community violence, political instability, and armed conflict has further increased risk of anxiety, depression, and trauma-related disorders [55]. Many adolescents encountered elevated domestic violence risk, family conflict, or bereavement after the loss of a caregiver strongly associated with later mental-health problems [45, 56]. Disruptions in schooling and peer relationships added to this burden [57]. Additionally, climate-related anxiety has emerged as a growing source of psychological distress [58]. These multifaceted drivers collectively explain the rising prevalence and burden of mental disorders and SUDs observed in our analysis, reinforcing the need for targeted interventions tailored to this developmentally vulnerable age group.

Addressing these global disparities requires targeted policy and service delivery reforms. Even in high-income countries like the United States, access to treatment for mental disorders and SUDs remains limited [59, 60]. Barriers include inadequate insurance coverage, provider shortages, and fragmented service delivery systems. To address this, the US Departments of Labor, Health and Human Services, and the Treasury have issued rules expanding equitable access to these services under private health coverage [61]. Evidence from a nationally representative survey of commercial health plans suggests that MHPAEA implementation has led to measurable improvements in behavioral health coverage [62]. However, the long-term implementation and stability of these initiatives have been uncertain, particularly given ongoing political pressures on federal health agencies and fluctuating administrative priorities. Other countries have adopted alternative strategies to expand access, particularly those with universal or socialized health systems that rely on centralized financing and integrated primary-care mental-health models [63, 64]. Canada has implemented collaborative care models and task-shifting strategies to address workforce shortages and improve access in underserved areas [65]. Rather than viewing any single system as a universal template, these varied approaches underscore the need for context-specific strategies. Effective reform must address financial, structural, and administrative barriers while promoting coordinated, community-based mental health care. Cross-national learning should focus on adaptable mechanisms such as task-shifting, outcome-based payment models, and digital mental health platforms that align with local workforce capacity and sociopolitical environments. Our study underscores the urgent need for targeted interventions to address pandemic-related mental health impacts and SUD rebound. Comprehensive, age-tailored public health interventions for adolescents and young adults are crucial. Additionally, sex-specific mental health interventions and policies are essential, given the differential impact of these disorders on males and females, requiring strategies tailored to each sex’s unique needs and challenges.

## Limitation

The study has limitations. It relies on the GBD dataset, which may have quality and completeness issues. While such issues have been more prominent in low-income countries, even in high-income countries the prevalence of certain mental disorders could be underestimated due to underdiagnosis and/or underreporting in primary care settings or persistent stigma surrounding mental health help-seeking. Diagnostic criteria and health reporting systems evolved during 1990–2021, causing inconsistencies in trend analysis. Additionally, socioeconomic and cultural factors (e.g., stigma, healthcare access, cultural perceptions) were not fully considered, potentially skewing results. While our study acknowledges COVID–19’s impact on mental health, it lacks updated data on long-term effects, and findings may not be generalized to all populations. Future studies should explore ongoing potential pandemic impacts.

## Conclusions

This study found a rising global burden of mental disorders and SUDs among adolescents and young adults from 1990–2021, with a surge in depressive and anxiety disorders during 2019–2021. SUDs decreased during the pandemic. Sex and regional differences were noted, with males more affected by ADHD, autism, and conduct disorders, and females by anorexia nervosa. High-income North America, Western Europe, and Australasia had the highest rates. Further research is needed to explore long-term pandemic impacts, especially regional and sex-specific differences, to improve public health policies and outcomes.

## Supplementary information

Supplementary Materials

## Data Availability

All data downloaded from GBD database (https://www.healthdata.org/researchanalysis/gbd).

## References

[1] Blakemore SJ. Adolescence and mental health. Lancet. 2019;393:2030–1. 31106741 10.1016/S0140-6736(19)31013-X

[2] Degenhardt L, Stockings E, Patton G, Hall WD, Lynskey M. The increasing global health priority of substance use in young people. Lancet Psychiatry. 2016;3:251–64. 26905480 10.1016/S2215-0366(15)00508-8

[3] Patel V, Saxena S, Lund C, Thornicroft G, Baingana F, Bolton P, et al. The Lancet Commission on global mental health and sustainable development. Lancet. 2018;392:1553–98. 30314863 10.1016/S0140-6736(18)31612-X

[4] World Health Organization: WHO. (2021, November 17). Mental health of adolescents. https://www.who.int/news-room/fact-sheets/detail/adolescent-mental-health. Accessed August 29, 2024.

[5] Castaldelli-Maia JM, Bhugra D. Analysis of global prevalence of mental and substance use disorders within countries: focus on sociodemographic characteristics and income levels. Int Rev Psychiatry. 2022;34:6–15. 35584016 10.1080/09540261.2022.2040450

[6] World Health O. Depression and other common mental disorders: global health estimates. Geneva: World Health Organization; 2017 2017. Contract No.: WHO/MSD/MER/2017.2.

[7] Chuang SP, Wu JYW, Wang CS. Resilience and quality of life in people with mental illness: a systematic review and meta-analysis. Neuropsychiatr Dis Treat. 2023;19:507–14. 36910331 10.2147/NDT.S392332 PMC9994666

[8] Grant BF, Saha TD, Ruan WJ, Goldstein RB, Chou SP, Jung J, et al. Epidemiology of DSM-5 drug use disorder: results from the national epidemiologic survey on alcohol and related conditions-III. JAMA Psychiatry. 2016;73:39–47. 26580136 10.1001/jamapsychiatry.2015.2132 PMC5062605

[9] Santucci K. Psychiatric disease and drug abuse. Curr Opin Pediatr. 2012;24:233–7. 22327950 10.1097/MOP.0b013e3283504fbf

[10] GBD 2019 Mental Disorders Collaborators. Global, regional, and national burden of 12 mental disorders in 204 countries and territories, 1990-2019: a systematic analysis for the Global Burden of Disease Study 2019. Lancet Psychiatry. 2022;9:137–50. 35026139 10.1016/S2215-0366(21)00395-3 PMC8776563

[11] Qu C, Chen Y, Lai Z, Feng T, Zhang H, Hu H, et al. Burden of drug use disorder among adolescents and young adults aged 10-24 years in 204 countries and territories from 1990 to 2019 and future prediction to 2044. Asian J Psychiatr. 2024;91:103835. 38029603 10.1016/j.ajp.2023.103835

[12] Bell IH, Nicholas J, Broomhall A, Bailey E, Bendall S, Boland A, et al. The impact of COVID-19 on youth mental health: a mixed methods survey. Psychiatry Res. 2023;321:115082. 36738592 10.1016/j.psychres.2023.115082 PMC9883078

[13] Kupcova I, Danisovic L, Klein M, Harsanyi S. Effects of the COVID-19 pandemic on mental health, anxiety, and depression. BMC Psychol. 2023;11:108. 37041568 10.1186/s40359-023-01130-5 PMC10088605

[14] Murray CJL. Findings from the Global Burden of Disease Study 2021. The Lancet. 2024;403:2259–62. 10.1016/S0140-6736(24)00769-4 38762327

[15] GBD 2021 Diseases and Injuries Collaborators. Global incidence, prevalence, years lived with disability (YLDs), disability-adjusted life-years (DALYs), and healthy life expectancy (HALE) for 371 diseases and injuries in 204 countries and territories and 811 subnational locations, 1990-2021: a systematic analysis for the global burden of disease study 2021. Lancet. 2024;403:2133–61. 38642570 10.1016/S0140-6736(24)00757-8 PMC11122111

[16] GBD 2021 Causes of Death Collaborators. Global burden of 288 causes of death and life expectancy decomposition in 204 countries and territories and 811 subnational locations, 1990-2021: a systematic analysis for the Global Burden of Disease Study 2021. Lancet. 2024;403:2100–32. 38582094 10.1016/S0140-6736(24)00367-2 PMC11126520

[17] GBD 2021 Diabetes Collaborators. Global, regional, and national burden of diabetes from 1990 to 2021, with projections of prevalence to 2050: a systematic analysis for the Global Burden of Disease Study 2021. Lancet. 2023;402:203–34. 37356446 10.1016/S0140-6736(23)01301-6 PMC10364581

[18] Wu Y, Wang L, Tao M, Cao H, Yuan H, Ye M, et al. Changing trends in the global burden of mental disorders from 1990 to 2019 and predicted levels in 25 years. Epidemiol Psychiatr Sci. 2023;32:e63. 37933540 10.1017/S2045796023000756 PMC10689059

[19] Rajji TK, Ismail Z, Mulsant BH. Age at onset and cognition in schizophrenia: meta-analysis. Br J Psychiatry. 2009;195:286–93. 19794194 10.1192/bjp.bp.108.060723

[20] Jiang S, Huang H, Zhou J, Li H, Duan M, Yao D, et al. Progressive trajectories of schizophrenia across symptoms, genes, and the brain. BMC Med. 2023;21:237. 37400838 10.1186/s12916-023-02935-2 PMC10318676

[21] Black DW. The natural history of antisocial personality disorder. Can J Psychiatry. 2015;60:309–14. 26175389 10.1177/070674371506000703 PMC4500180

[22] Chadi N, Bagley SM, Hadland SE. Addressing adolescents’ and young adults’ substance use disorders. Med Clin North Am. 2018;102:603–20. 29933818 10.1016/j.mcna.2018.02.015

[23] Jurewicz I. Mental health in young adults and adolescents - supporting general physicians to provide holistic care. Clin Med (Lond). 2015;15:151–4. 25824067 10.7861/clinmedicine.15-2-151 PMC4953734

[24] Jenkins R, Baingana F, Ahmad R, McDaid D, Atun R. Social, economic, human rights and political challenges to global mental health. Ment Health Fam Med. 2011;8:87–96. 22654971 PMC3178190

[25] Ridley M, Rao G, Schilbach F, Patel V. Poverty, depression, and anxiety: causal evidence and mechanisms. Science. 2020;370:eaay0214. 33303583 10.1126/science.aay0214

[26] Steare T, Gutiérrez Muñoz C, Sullivan A, Lewis G. The association between academic pressure and adolescent mental health problems: a systematic review. J Affect Disord. 2023;339:302–17. 37437728 10.1016/j.jad.2023.07.028

[27] Jiang MM, Gao K, Wu ZY, Guo PP. The influence of academic pressure on adolescents’ problem behavior: Chain mediating effects of self-control, parent-child conflict, and subjective well-being. Front Psychol. 2022;13:954330. 36211862 10.3389/fpsyg.2022.954330 PMC9534181

[28] Craig W, Boniel-Nissim M, King N, Walsh SD, Boer M, Donnelly PD, et al. Social media use and cyber-bullying: a cross-national analysis of young people in 42 countries. Journal of Adolescent Health. 2020;66:S100–S8. 10.1016/j.jadohealth.2020.03.006 32446603

[29] Zhu C, Huang S, Evans R, Zhang W. Cyberbullying among adolescents and children: a comprehensive review of the global situation, risk factors, and preventive measures. Front Public Health. 2021;9:634909. 33791270 10.3389/fpubh.2021.634909 PMC8006937

[30] Peprah P, Oduro MS, Atta-Osei G, Addo IY, Morgan AK, Gyasi RM. Problematic social media use mediates the effect of cyberbullying victimisation on psychosomatic complaints in adolescents. Scientific Reports. 2024;14:9773. 38684725 10.1038/s41598-024-59509-2 PMC11058249

[31] Madsen KR, Damsgaard MT, Petersen K, Qualter P, Holstein BE. Bullying at school, cyberbullying, and loneliness: national representative study of adolescents in denmark. Int J Environ Res Public Health. 2024;21:414. 38673326 10.3390/ijerph21040414 PMC11050631

[32] Zhang X, Mori Y, Abio A, Khorasani ZK, Gilbert S, Grimland M, et al. Cross-national research on adolescent mental health: a systematic review comparing research in low, middle and high-income countries. BMJ Global Health. 2025;10:e019267. 40713084 10.1136/bmjgh-2025-019267 PMC12306219

[33] Rathod S, Pinninti N, Irfan M, Gorczynski P, Rathod P, Gega L, et al. Mental health service provision in low- and middle-income countries. Health Serv Insights. 2017;10:1178632917694350. 28469456 10.1177/1178632917694350 PMC5398308

[34] Mayston R, Frissa S, Tekola B, Hanlon C, Prince M, Fekadu A. Explanatory models of depression in sub-Saharan Africa: synthesis of qualitative evidence. Soc Sci Med. 2020;246:112760. 32006814 10.1016/j.socscimed.2019.112760 PMC7014569

[35] Bovey M, Hosny N, Dutray F, Heim E. Trauma-related cultural concepts of distress: a systematic review of qualitative literature from the middle east and North Africa, and Sub-Saharan Africa. SSM - Mental Health. 2025;7:100402.

[36] Atewologun F, Adigun OA, Okesanya OJ, Hassan HK, Olabode ON, Micheal AS, et al. A comprehensive review of mental health services across selected countries in sub-Saharan Africa: assessing progress, challenges, and future direction. Discov Ment Health. 2025;5:49. 40195169 10.1007/s44192-025-00177-7 PMC11977099

[37] Rameez S, Nasir A. Barriers to mental health treatment in primary care practice in low- and middle-income countries in a post-covid era: a systematic review. J Family Med Prim Care. 2023;12:1485–504. 37767443 10.4103/jfmpc.jfmpc_391_22 PMC10521856

[38] Gopalkrishnan N. Cultural diversity and mental health: considerations for policy and practice. Front Public Health. 2018;6:179. 29971226 10.3389/fpubh.2018.00179 PMC6018386

[39] Cheng ZH, Perko VL, Fuller-Marashi L, Gau JM, Stice E. Ethnic differences in eating disorder prevalence, risk factors, and predictive effects of risk factors among young women. Eat Behav. 2019;32:23–30. 30529736 10.1016/j.eatbeh.2018.11.004 PMC6382562

[40] Striegel-Moore RH, Rosselli F, Perrin N, DeBar L, Wilson GT, May A, et al. Gender difference in the prevalence of eating disorder symptoms. Int J Eat Disord. 2009;42:471–4. 19107833 10.1002/eat.20625 PMC2696560

[41] Posserud MB, Skretting Solberg B, Engeland A, Haavik J, Klungsøyr K. Male to female ratios in autism spectrum disorders by age, intellectual disability and attention-deficit/hyperactivity disorder. Acta Psychiatr Scand. 2021;144:635–46. 34494265 10.1111/acps.13368

[42] Brooks Holliday S, Ewing BA, Storholm ED, Parast L, D’Amico EJ. Gender differences in the association between conduct disorder and risky sexual behavior. J Adolesc. 2017;56:75–83. 28182979 10.1016/j.adolescence.2017.01.008 PMC5504918

[43] Almeida M, Shrestha AD, Stojanac D, Miller LJ. The impact of the COVID-19 pandemic on women’s mental health. Arch Womens Ment Health. 2020;23:741–8. 33263142 10.1007/s00737-020-01092-2 PMC7707813

[44] Li SH, Graham BM. Why are women so vulnerable to anxiety, trauma-related and stress-related disorders? The potential role of sex hormones. Lancet Psychiatry. 2017;4:73–82. 27856395 10.1016/S2215-0366(16)30358-3

[45] Peterman A, Potts A, O’Donnell M, Thompson K, Shah N, Oertelt-Prigione S, et al. Pandemics and Violence Against Women and Children. Center for Global Development; 2020.

[46] Sánchez OR, Vale DB, Rodrigues L, Surita FG. Violence against women during the COVID-19 pandemic: an integrative review. Int J Gynaecol Obstet. 2020;151:180–7. 32880941 10.1002/ijgo.13365 PMC9087782

[47] Loades ME, Chatburn E, Higson-Sweeney N, Reynolds S, Shafran R, Brigden A, et al. Rapid systematic review: the impact of social isolation and loneliness on the mental health of children and adolescents in the context of COVID-19. J Am Acad Child Adolesc Psychiatry. 2020;59:1218–39.e3. 32504808 10.1016/j.jaac.2020.05.009 PMC7267797

[48] Hawdon J, Parti K, Dearden T. Changes in online illegal drug buying during COVID-19: assessing effects due to a changing market or changes in strain using a longitudinal sample design. American journal of criminal justice. 2022;47:712–34. 36407841 10.1007/s12103-022-09698-1 PMC9649405

[49] Branje S. The impact of the COVID-19 pandemic on adolescent mental health across the world. Curr Opin Psychol. 2023;53:101665. 37562339 10.1016/j.copsyc.2023.101665

[50] Creswell C, Shum A, Pearcey S, Skripkauskaite S, Patalay P, Waite P. Young people’s mental health during the COVID-19 pandemic. Lancet Child Adolesc Health. 2021;5:535–7. 34174991 10.1016/S2352-4642(21)00177-2 PMC9765398

[51] Odgers CL, Jensen MR. Annual Research Review: Adolescent mental health in the digital age: facts, fears, and future directions. J Child Psychol Psychiatry. 2020;61:336–48. 31951670 10.1111/jcpp.13190 PMC8221420

[52] Nixon CL. Current perspectives: the impact of cyberbullying on adolescent health. Adolesc Health Med Ther. 2014;5:143–58. 25177157 10.2147/AHMT.S36456 PMC4126576

[53] Johri K, Pillai R, Kulkarni A, Balkrishnan R. Effects of sleep deprivation on the mental health of adolescents: a systematic review. Sleep Science and Practice. 2025;9:9.

[54] Guthold R, Stevens GA, Riley LM, Bull FC. Global trends in insufficient physical activity among adolescents: a pooled analysis of 298 population-based surveys with 1·6 million participants. Lancet Child Adolesc Health. 2020;4:23–35. 31761562 10.1016/S2352-4642(19)30323-2 PMC6919336

[55] Charlson F, van Ommeren M, Flaxman A, Cornett J, Whiteford H, Saxena S. New WHO prevalence estimates of mental disorders in conflict settings: a systematic review and meta-analysis. The Lancet. 2019;394:240–8. 10.1016/S0140-6736(19)30934-1 PMC6657025 31200992

[56] Aguirre LVC, Jaramillo AK, Saucedo Victoria TE, Botero Carvajal A. Mental health consequences of parental death and its prevalence in children: a systematic literature review. Heliyon. 2024;10:e24999. 38304821 10.1016/j.heliyon.2024.e24999 PMC10830864

[57] Thompson KN, Oginni O, Wertz J, Danese A, Okundi M, Arseneault L, et al. Social isolation and poor mental health in young people: testing genetic and environmental influences in a longitudinal cohort study. Eur Child Adolesc Psychiatry. 2025;34:1445–55. 39259339 10.1007/s00787-024-02573-w PMC12000192

[58] Hickman C, Marks E, Pihkala P, Clayton S, Lewandowski RE, Mayall EE, et al. Climate anxiety in children and young people and their beliefs about government responses to climate change: a global survey. Lancet Planet Health. 2021;5:e863–e73. 34895496 10.1016/S2542-5196(21)00278-3

[59] Mongelli F, Georgakopoulos P, Pato MT. Challenges and opportunities to meet the mental health needs of underserved and disenfranchised populations in the United States. Focus (Am Psychiatr Publ). 2020;18:16–24. 32047393 10.1176/appi.focus.20190028 PMC7011222

[60] Liu L, Zhang C, Bonny AE, Nahata MC Strategies to improve access to care for patients with opioid use disorder. Ann Pharmacother. 2024:10600280241273258. 10.1177/10600280241273258 39229941

[61] US Department of Health and Human Services (2024, September 9) Departments of Labor, Health and Human Services, and the Treasury issue final rules strengthening access to mental health and substance use disorder benefits https://www.dol.gov/newsroom/releases/ebsa/ebsa20240909.

[62] Hodgkin D, Horgan CM, Stewart MT, Quinn AE, Creedon TB, Reif S, et al. Federal parity and access to behavioral health care in private health plans. Psychiatr Serv. 2018;69:396–402. 29334882 10.1176/appi.ps.201700203 PMC8508592

[63] Calderon J, Rojas G. Integration of mental health into primary care: a chilean perspective on a global challenge. BJPsych Int. 2016;13:20–1. 29093888 10.1192/s2056474000000945 PMC5618894

[64] Patel V, Saxena S. Achieving universal health coverage for mental disorders. Bmj. 2019;366:l4516. 31548204 10.1136/bmj.l4516 PMC6753845

[65] Kates N, Sunderji N, Ng V, Patriquin M, Alloo J, Mirwaldt P, et al. Collaborative mental health care in Canada: challenges, opportunities and new directions. Can J Psychiatry. 2023;68:372–98. 36688252 10.1177/07067437221102201 PMC10192825

