# Supplementary material for: The global burden of mental and substance use disorders among adolescents and young adults
Source: Mol Psychiatry. 2026 Feb 28;31(7):3880–97. doi: 10.1038/s41380-026-03503-9 (PMC13269128; doi:10.1038/s41380-026-03503-9)
Supplement: Supplementary file 1 — Supplementary Materials [file 41380_2026_3503_MOESM1_ESM.docx]

**Supplementary Materials**

**Table S1** Definitions for included causes in the Global Burden of Disease database

| **Causes** | **Definition** |
| --- | --- |
| **Anxiety disorders** | Anxiety disorders are characterised by experiences of intense fear and distress, typically in combination with other physiological symptoms. We aimed to capture all cases of anxiety disorders reaching diagnostic threshold defined by the Diagnostic and Statistical Manual of Mental Disorders (DSM) or the World Health Organization (WHO) International Classification of Diseases (ICD). The specific anxiety disorders included were panic disorder, agoraphobia, specific phobia, social phobia, obsessivecompulsive disorder (OCD), post-traumatic stress disorder (PTSD), generalised anxiety disorder (GAD) including overanxious disorder in childhood, separation anxiety disorder (SAD), and anxiety disorder“not otherwise specified”(NOS). These were identified by the following codes: DSM-IV-TR: 300.0-300.3, 208.3, 309.21, 309.81; ICD-10: F40-42, F43.0, F43.1, F93.0-93.2, F93.8. Excluded were anxiety disorders due to a general medical condition and substance-induced anxiety disorder. Different versions of DSM (DSM-III, DSM-III-R, DSM-IV, DSM-IV-TR, DSM-5, and DSM-5-TR) and ICD (ICD-9, ICD-10, and ICD-11) were accepted. |
| **Attention-deficit/hyperactivity disorder** | Attention-deficit/hyperactivity disorder (ADHD) is an externalising disorder characterised by persistent inattention and/or hyperactivity-impulsivity. As per criteria set by the Diagnostic and Statistical Manual of Mental Disorders Fourth Edition, Text Revision (DSM-IV-TR), diagnosis requires six or more symptoms of inattention or hyperactivity-impulsivity to have persisted for at least six months in two or more settings causing significant impairment to functioning, with at least some impairing symptoms being present prior to 7 years of age (12 years of age in DSM-5). Included in the GBD study were cases meeting diagnostic criteria according to DSM1 or the International Classification of Diseases (ICD) (called “hyperkinetic disorder” in ICD). These were identified by the  following codes: 314.0, 314.01 (DSM-IV-TR) and F90 (ICD-10). Different versions of DSM (DSM-III, DSM-IIIR, DSM-IV, DSM-IV-TR, DSM-5, and DSM-5-TR) and ICD (ICD-9, ICD-10, and ICD-11) were accepted. |
| **Autism spectrum disorders** | Autism spectrum disorders (ASD)–also known as pervasive developmental disorders–are a group of neurodevelopmental disorders with onset occurring in early childhood. ASD is characterised by pervasive impairment in several areas of development, including social interaction and communication skills, along with restricted and repetitive patterns of behaviours and/or interests. ASD was an umbrella for five sub-disorders according to the Diagnostic and Statistical Manual of Mental Disorders fourth edition, text revision (DSM-IV-TR): autistic disorder (299.00), pervasive developmental disorder, pervasive developmental disorder not otherwise specified (299.80), Rett’s disorder (299.8), Asperger’s disorder (299.8), and childhood disintegrative disorder (299.10). ASD is still an umbrella for 152 eight sub-disorders according to the International Statistical Classification of Diseases and Related Health Problems 10th Revision (ICD-10): childhood autism (F84.0), atypical autism (F84.1), Rett’s syndrome (F84.2), other childhood disintegrative disorder (F84.3), overactive disorder associated with mental retardation and stereotyped movements (F84.4), Asperger syndrome (F84.5), other pervasive developmental disorders (F84.8), and pervasive disorder unspecified (F84.9). However, it has been amalgamated into a single disorder in the Diagnostic and Statistical Manual for Mental Disorders 5th edition (DSM-5). Different versions of DSM (DSM-III, DSM-III-R, DSM-IV, DSM-IV-TR, DSM-5, and DSM-5-TR) and ICD (ICD9, ICD-10, and ICD-11) were accepted. |
| **Bipolar disorders** | Bipolar disorder is a serious mood disorder with little or no complete remission. Included in GBD disease modelling were cases meeting diagnostic criteria for bipolar disorder according to the Diagnostic and Statistical Manual of Mental Disorders (DSM), or the equivalent diagnosis in the International Classification of Diseases (ICD). These are identified by the following codes: DSM-IV-TR: 296.0-296.7, 296.89, 301.13; ICD-10: F30.0-F30.9, F31.0-F31.6, F31.8-F31.9, F34.0. Excluded were bipolar disorder due to a general medical condition or substance-induced cases. Different versions of DSM (DSM-III, DSM-III-R, DSM-IV, DSM-IV-TR, DSM-5, and DSM-5-TR) and ICD (ICD-9, ICD-10, and ICD-11) were accepted. |
| **Conduct disorders** | Conduct disorder (CD) is an externalising behaviour disorder characterised by a pattern of antisocial behaviour that violates the basic rights of others or major age-appropriate societal norms. As per criteria set by the Diagnostic and Statistical Manual of Mental Disorders fourth edition, text revision (DSM-IVTR), diagnosis requires three or more of the following symptoms to be present in the past 12 months (with at least one present in the last six months) and cause significant impairment in functioning.  CD is considered a disorder of childhood but can be diagnosed in adults who display such behaviours yet do not meet the criteria for antisocial personality disorder. However, there are almost no studies measuring adult CD as existing studies in this area tend to measure adult antisocial behaviour rather than adult CD. As such, only childhood CD (ie, cases prior to 18 years of age) was modelled in GBD. Included in GBD were cases meeting diagnostic criteria according to DSM or the International Classification of Diseases (ICD). These were identified by the following codes: 312.81-312.89 (DSM-IV-TR) and F91 (ICD-10). Different versions of DSM (DSM-III, DSM-III-R, DSM-IV, DSM-IV-TR, DSM-5, and DSM-5-TR) and ICD (ICD-9, ICD-10, and ICD-11) were accepted. |
| **Depressive disorders** | Depressive disorders consist of Major Depressive Disorder (MDD) and Dysthymia. Major depressive disorder (MDD) is an episodic mood disorder involving the experience of one or more major depressive episode(s). Included in the GBD disease modelling were cases meeting diagnostic criteria for MDD according to the Diagnostic and Statistical Manual of Mental Disorders (DSM) or the equivalent diagnosis of recurrent depression in the International Classification of Diseases (ICD). These were identified by the following codes: DSM-IV-TR: 296.21–24, 296.31–34; ICD-10: F32.0–9, F33.0–9; excluding those cases due to a general medical condition or substance-induced cases. Different versions of DSM (DSM-III, DSM-III-R, DSM-IV, DSM-IV-TR, DSM-5, and DSM-5-TR) and ICD (ICD-9, ICD-10, and ICD-11) were accepted. Dysthymia is a mood disorder consisting of chronic depression, demonstrating less severe but longerlasting symptoms than major depressive disorder. Included in GBD disease modelling were cases meeting diagnostic criteria for dysthymia according to the Diagnostic and Statistical Manual of Mental Disorders (DSM), or the equivalent diagnosis in the International Classification of Diseases (ICD). These were identified by the following codes: DSM-IV-TR: 300.4, ICD-10: F34.1; excluding those cases due to a general medical condition or substance-induced cases. Different versions of DSM (DSM-III, DSM-III-R, DSM-IV, DSM-IV-TR, DSM-5, and DSM-5-TR) and ICD (ICD-9, ICD-10, and ICD-11) were accepted. |
| **Eating disorders** | Eating disorders consist of anorexia nervosa and bulimia nervosa.  According to the Diagnostic and Statistical Manual of Mental Disorders fourth edition, text revision (DSM- IV-TR), anorexia nervosa (AN) is an eating disorder characterised by: a) Refusal to maintain body weight at or above a minimally normal weight for age and height (eg, weight loss leading to maintenance of body weight less than 85% of that expected; or failure to make expected weight gain during period of growth, leading to body weight less than 85% of that expected). b) Intense fear of gaining weight or becoming fat, even though underweight (expanded to include any behaviour that interferes with weight gain in DSM-5). c) Disturbance in the way in which one’s body weight or shape is experienced, undue influence of body weight or shape on self-evaluation, or denial of the seriousness of the current low body weight. d) In postmenarcheal females, amenorrhoea, ie, the absence of at least three consecutive menstrual cycles (this criterion was removed in DSM-5). Included in the GBD study were cases meeting diagnostic criteria according to DSM or the International Classification of Diseases (ICD). These were identified by the following codes: 307.1 (DSM-IV-TR) and F50.0-50.1 (ICD-10). Different versions of DSM (DSM-III, DSM-III-R, DSM-IV, DSM-IV-TR, DSM-5, and DSM5-TR) and ICD (ICD-9, ICD-10 and ICD-11) were accepted.  According to the Diagnostic and Statistical Manual of Mental Disorders fourth edition, text revision (DSMIV-TR), bulimia nervosa (BN) is an eating disorder characterised by: a) Recurrent episodes of binge eating. An episode of binge eating is characterised by both of the following: 1) eating, in a discrete period of time (eg, within any two-hour period), an amount of food that is definitely larger than most people would eat during a similar period of time and under similar circumstances 2) a sense of lack of control over eating during the episode (eg, a feeling that one cannot stop eating or control what or how much one is eating) b) Recurrent inappropriate compensatory behaviour in order to prevent weight gain, such as selfinduced vomiting; misuse of laxatives, diuretics, enemas, or other medications; fasting; or excessive exercise. c) The binge eating and inappropriate compensatory behaviours both occur, on average, at least twice a week for three months (changed to once a week for three months in DSM-5). d) Self-evaluation is unduly influenced by body shape and weight. e) The disturbance does not occur exclusively during episodes of anorexia nervosa. Included in GBD were cases meeting diagnostic criteria according to DSM or the International Classification of Diseases (ICD). These were identified by the following codes: 307.51 (DSM-IV-TR) and F50.2 (ICD-10). Different versions of DSM (DSM-III, DSM-III-R, DSM-IV, DSM-IV-TR, DSM-5, and DSM-5-TR) and ICD (ICD-9, ICD-10, and ICD-11) were accepted. |
| **Idiopathic** **developmental intellectual disability** | Idiopathic developmental intellectual disability (IDID) (ICD-10 F70-F79.9) is a condition characterised by significant limitations in both intellectual functioning and adaptive behavior. Consistent with the American Association on Intellectual and Developmental Disabilities, we define developmental intellectual disability as a condition originating before age 18 (as such, it does not include impairment due to stroke, Alzheimer’s disease, or other conditions that affect older populations). We model the severities shown in Table 1, as measured by score on intelligence quotient (IQ) tests, which are standardised to have a mean of 100. Commonly used IQ tests include: Wechsler Preschool and Primary Scale of Intelligence (WIPPSI), Wechsler Intelligence Scale for Children (WISC), and Wechsler Adult Intelligence Scale (WAIS). |
| **Schizophrenia** | Schizophrenia is a chronic psychotic disorder which involves the experience of positive symptoms (eg, delusions, hallucinations, thought disorder) and negative symptoms (eg, flat affect, loss of interest, and emotional withdrawal). Included in the GBD disease modelling were cases meeting the Diagnostic and Statistical Manual of Mental Disorders (DSM) or the International Classification of Diseases (ICD) diagnostic criteria for schizophrenia (DSM-IV-TR: 295.10-295.30, 295.60, 295.90; ICD 10: F20). Different versions of DSM (DSM-III, DSM-III-R, DSM-IV, DSM-IV-TR, DSM-5, and DSM5-TR) and ICD (ICD-9, ICD-10, and ICD-11) were accepted. |
| **Other mental disorders** | In addition to the individual mental disorders for which we estimate burden, we also estimate the nonfatal burden attributable to a residual cause of “other mental disorders.” This is made up of an aggregate group of personality disorders. Personality disorders are characterised by pervasive, inflexible and maladaptive patterns of behaviour and inner experience which are markedly different from what is considered to be acceptable in the individual’s culture. These disorders tend to be chronic and are associated with significant distress or disability. Included in GBD 2021 were cases meeting diagnostic criteria for personality disorders according to the Diagnostic and Statistical Manual of Mental Disorders (DSM-IV-TR: 300.3, 301.0; 301.2, 301.22, 301.5–301.9), or the equivalent diagnosis in the International Classification of Diseases (ICD-10: F60). |
| **Alcohol use disorders** | Alcohol dependence is a substance-related disorder involving a dysfunctional pattern of alcohol use. According to the Diagnostic and Statistical Manual of Mental Disorders (DSM-IV) criteria for alcohol dependence, at least three out of seven of the following criteria must be manifested during a 12-month period:  ▪ Tolerance  ▪ Withdrawal symptoms or clinically defined alcohol withdrawal syndrome  ▪ Use in larger amounts or for longer periods than intended  ▪ Persistent desire or unsuccessful efforts to cut down on alcohol use  ▪ Time is spent obtaining alcohol or recovering from effects  ▪ Social, occupational, and recreational pursuits are given up or reduced because of alcohol use  ▪ Use is continued despite knowledge of alcohol-related harm (physical or psychological)  The DSM-IV codes for alcohol dependence is 303.90, and the corresponding International Classification of Diseases (ICD-10) codes are F10.1 and F10.2. |
| **Drug use disorders** | In addition to the four drug use disorders for which we specifically estimate non-fatal burden (opioid, cocaine, amphetamine, and cannabis dependence), we also estimate the burden attributable to a residual cause of “other drug use disorders.” This is made up of an aggregate group of other forms of drug dependence. Included in the Global Burden of Disease (GBD) modelling were cases meeting the Diagnostic and Statistical Manual of Mental Disorders (DSM-IV-TR) or the International Classification of  Diseases (ICD-10) diagnostic criteria for:  • Hallucinogen dependence  • Inhalant or solvent dependence  • Sedative dependence  • Tranquiliser dependence  • Other medicines, drugs, substance dependence  According to DSM-IV TR criteria, dependence involves a maladaptive pattern of substance use, leading to clinically significant impairment or distress. At least three of the following symptoms must be experienced within the same 12-month period:  • Tolerance, characterised by either  • a need for increased amounts of the substance to achieve intoxication; or  • markedly diminished effect with continued use of the same amount of the substance;  • Withdrawal, characterised by either  • Withdrawal symptoms characteristic to dependence; or  • the same (or similar) substance is taken to avoid withdrawal symptoms;  • Substance taken in progressively larger amounts or for longer periods;  • Persistent desire or unsuccessful efforts to reduce substance use;  • Disproportionate time dedicated to obtaining the substance;  • Other important activities are given up because of the substance use; and  • Substance use is continued despite knowledge of physical or psychological problems occurring as a result of the substance. |

**Table S2** Global prevalence counts for mental disorders and substance use disorders, age 10-19 and 20-24, in 2021

| **Conditions** | **Prevalence Counts (95% UI)** | |
| --- | --- | --- |
|  | **10-19 year** | **20-24 year** |
| **Mental disorders** |  |  |
| Total | 184,875,218 (161,325,044- 210,243,315) | 94,102,083 (81,520,099- 107,696,631) |
| Male | 96,287,677 (83,990,160- 109,328,178) | 43,915,599 (38,350,673- 49,850,733) |
| Female | 88,587,541 (76,927,065- 101,362,223) | 50,186,484 (43,039,540- 57,796,619) |
| **Schizophrenia** |  |  |
| Total | 499,177 (316,663- 755,121) | 1,496,095 (968,687- 2,192,062) |
| Male | 273,619 (174,034- 411,517) | 807,081 (522,949- 1,174,043) |
| Female | 225,558 (142,296- 342,615) | 689,014 (445,937- 1,016,031) |
| **Depressive disorders** |  |  |
| Total | 29,523,221 (21,480,356- 38,338,971) | 27,965,580 (21,041,568- 38,573,571) |
| Male | 11,487,310 (8,344,385- 14,999,653) | 11,247,415 (8,386,918- 15,390,109) |
| Female | 18,035,911 (13,207,147- 23,353,580) | 16,718,166 (12,607,117- 23,077,956) |
| **Major Depressive disorder** |  |  |
| Total | 23,890,085 (16,335,579- 31,595,729) | 21,146,917 (14,680,294- 31,345,517) |
| Male | 9,146,361 (6,208,395- 12,145,462) | 8,429,184 (5,926,748- 12,393,462) |
| Female | 14,743,724 (10,155,001- 19,507,510) | 12,717,733 (8,745,350- 18,995,240) |
| **Dysthymia** |  |  |
| Total | 5,781,398 (3,966,644- 8,221,115) | 7,091,082 (4,925,853- 9,796,411) |
| Male | 2,384,288 (1,636,176- 3,401,024) | 2,903,000 (2,007,133- 4,013,843) |
| Female | 3,397,110 (2,352,064- 4,823,075) | 4,188,082 (2,931,244- 5,780,196) |
| **Bipolar disorder** |  |  |
| Total | 3,769,393 (2,699,586- 5,256,689) | 4,044,350 (5,520,531- 2,956,261) |
| Male | 1,861,059 (1,340,749- 2,585,055) | 1,979,549 (1,451,963- 2,695,670) |
| Female | 1,908,333 (1,361,598- 2,666,861) | 2,064,800 (1,504,243- 2,824,860) |
| **Anxiety disorders** |  |  |
| Total | 60,121,092 (45,203,514- 79,014,627) | 33,826,064 (24,956,762- 44,703,689) |
| Male | 23,744,069 (17,686,203- 31,492,075) | 12,873,994 (9,385,137- 17,173,485) |
| Female | 36,377,023 (27,309,233-47,506,056) | 20,952,071 (15,481,127- 27,599,627) |
| **Eating disorders** |  |  |
| Total | 3,342,617 (2,188,324- 5,047,225) | 3,398,660 (2,110,852- 5,130,709) |
| Male | 1,264,206 (793,205- 1,994,006) | 1,170,879 (665,573- 1,942,154) |
| Female | 2,078,411 (1,398,086- 3,141,994) | 2,227,781 (1,425,531- 3,214,328) |
| **Anorexia nervosa** |  |  |
| Total | 1,230,018 (780,633- 1,873,779) | 862,644 (525,595- 1,309,421) |
| Male | 382,625 (236,980- 595,095) | 237,364 (144,663- 361,481) |
| Female | 847,393 (545,378- 1,285,635) | 625,279 (380,002- 946,396) |
| **Bulimia nervosa** |  |  |
| Total | 2,116,409 (1,148,629- 3,755,143) | 2,541,839 (1,278,512- 4,360,663) |
| Male | 882,291 (477,006- 1,606,815) | 934,351 (408,809- 1,694,522) |
| Female | 1,234,117 (666,146- 2,189,743) | 1,607,488 (848,862- 2,654,996) |
| **ASD** |  |  |
| Total | 10,791,420 (9,101,247- 12,668,007) | 4,899,809 (4,131,439- 5,745,809) |
| Male | 7,342,829 (6,203,541- 8,580,516) | 3,314,960 (2,796,574- 3,871,945) |
| Female | 3,448,591 (2,892,415- 4,096,787) | 1,584,849 (1,324,886- 1,881,556) |
| **ADHD** |  |  |
| Total | 31,549,659 (21,648,747- 44,836,991) | 9,480,741 (6,551,628- 13,210,785) |
| Male | 22,946,319 (15,789,875- 32,455,813) | 6,780,678 (4,743,127- 9,442,687) |
| Female | 8,603,340 (5,896,903- 12,394,380) | 2,700,063 (1,821,868- 3,760,511) |
| **Conduct disorder** |  |  |
| Total | 33,059,530 (23,204,331-43,388,640) | 656,172 (367,255- 1,037,803) |
| Male | 21,244,261 (15,098,427- 27,368,501) | 514,854 (306,438- 782,004) |
| Female | 11,815,269 (7,903,330- 16,063,344) | 141,318 (58,436- 250,505) |
| **IDID** |  |  |
| Total | 20,930,749 (11,843,597- 29,689,962) | 8,845,357 (4,953,002- 12,693,766) |
| Male | 10,525,145 (5,597,710- 15,276,662) | 4,358,777 (2,284,880- 6,402,689) |
| Female | 10,405,604 (6,243,062- 14,409,851) | 4,486,580 (2,666,892- 6,275,132) |
| **Other mental disorders** |  |  |
| Total | 2,953,394 (1,833,144- 4,262,566) | 6,089,754 (3,986,900- 8,626,324) |
| Male | 1,852,232 (1,157,341- 2,675,402) | 3,778,789 (2,487,075- 5,372,214) |
| Female | 1,101,163 (675,932- 1,589,061) | 2,310,965 (1,492,371- 3,270,671) |
| **Substance Use Disorders** |  |  |
| Total | 10,097,650 (7,534,493- 13,171,016) | 19,615,300 (16,036,536- 23,855,746) |
| Male | 6,626,986 (4,971,917- 8,638,831) | 13,072,821 (10,633,469- 15,993,146) |
| Female | 3,470,664 (2,574,547- 4,529,259) | 6,542,479 (5,394,380- 8,028,419) |
| **Alcohol use disorder** |  |  |
| Total | 3,655,437 (2,435,221- 5,243,344) | 8,794,009 (5,981,839- 12,574,808) |
| Male | 2,539,473 (1,701,821- 3,647,086) | 6,447,417 (4,474,993- 9,046,059) |
| Female | 1,115,965 (725,837- 1,648,259) | 2,346,592 (1,521,174- 3,498,719) |
| **Drug use disorder** |  |  |
| Total | 6,497,584 (4,402,491- 9,141,403) | 11,076,946 (9,087,294- 13,870,854) |
| Male | 4,128,025 (2,774,722- 5,866,026) | 6,820,060 (5,583,786- 8,549,815) |
| Female | 2,369,559 (1,629,002- 3,306,257) | 4,256,886 (3,502,734- 5,382,178) |

95% UI, 95% uncertainty interval; YLDs, Years Lived with Disability; DALYs, Disability-adjusted life years; ADHD, Attention-deficit/hyperactivity disorder; ASD, Autism spectrum disorders; IDID, Idiopathic developmental intellectual disability; All prevalence estimates in this study refer to point prevalence

|  | **DALYs** | **Prevalence** |
| --- | --- | --- |
| **Age**  **10-19** | **A** | **B** |
|  | 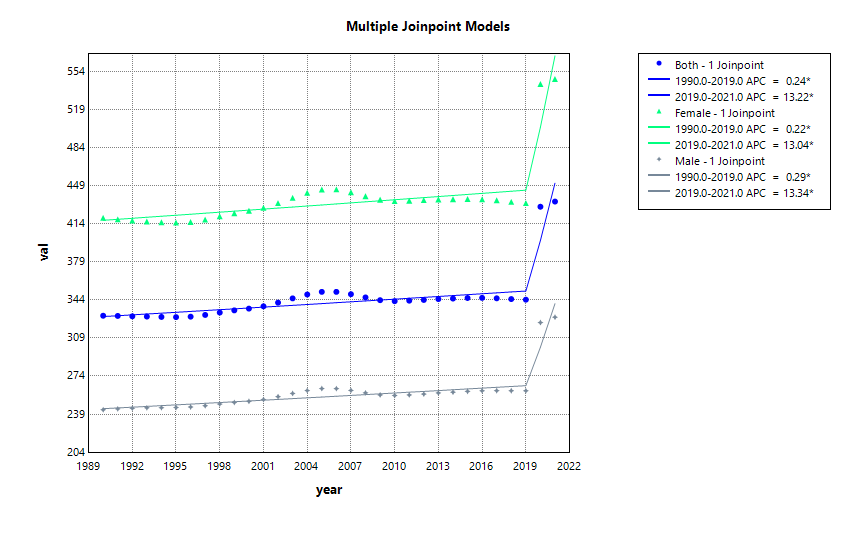 | 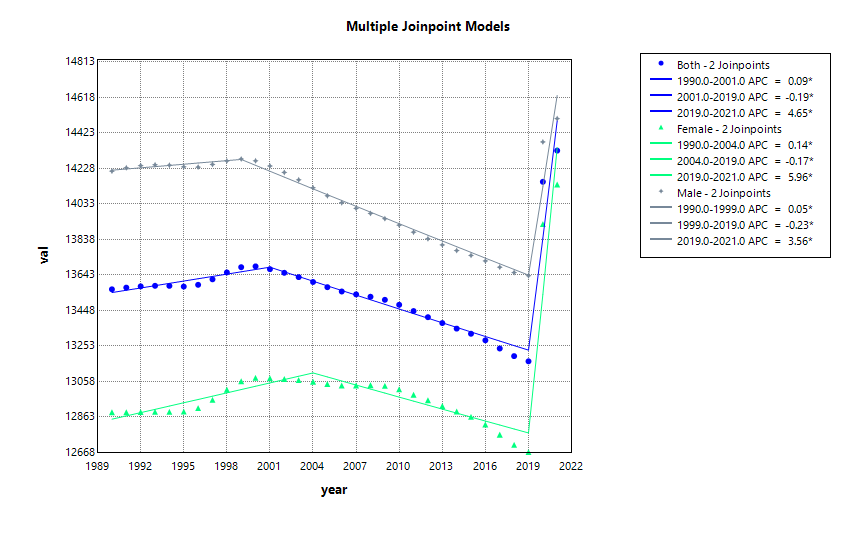 |
| **Age**  **20-24** | **C** | **D** |
|  | 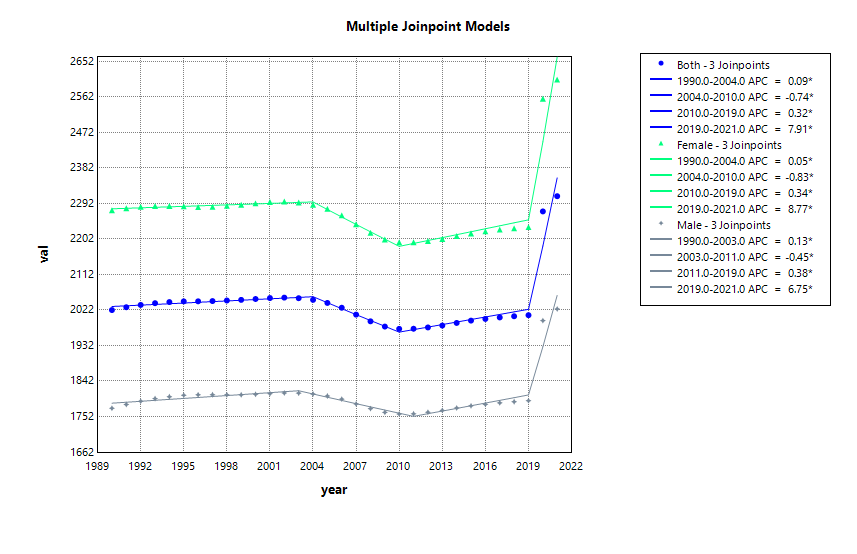 | 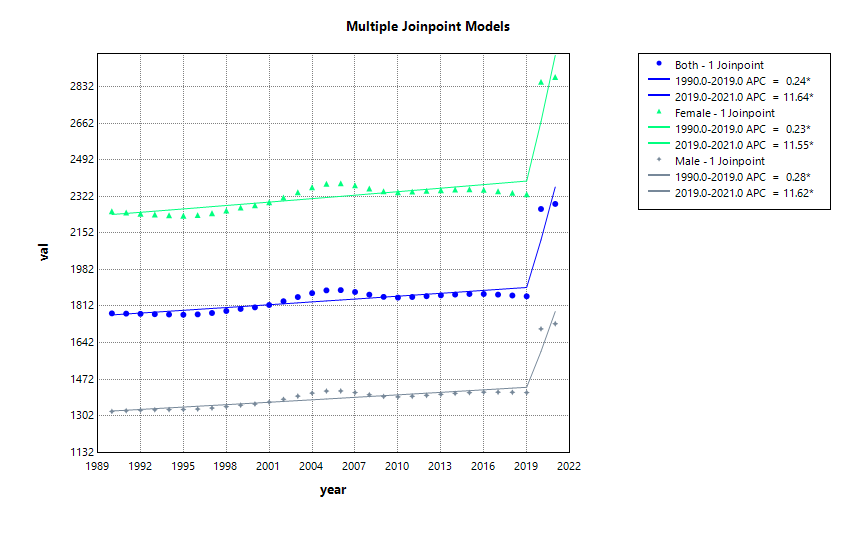 |

**Figure S1** Joinpoint regression analysis results of depressive disorders: Disability-adjusted life years, prevalence, and years lived with disability by two age groups and sex. APC, annual percentage change; DALYs, disability-adjusted life-years; YLDs, years lived with disability; All prevalence estimates in this study refer to point prevalence; * means significance

|  | **DALYs** | **Prevalence** |
| --- | --- | --- |
| **Age**  **10-19** | **A** | **B** |
|  | 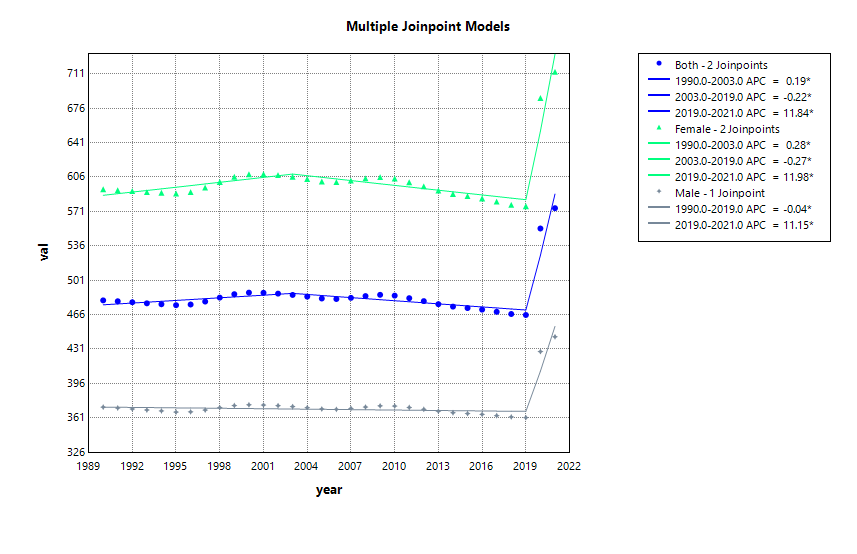 | 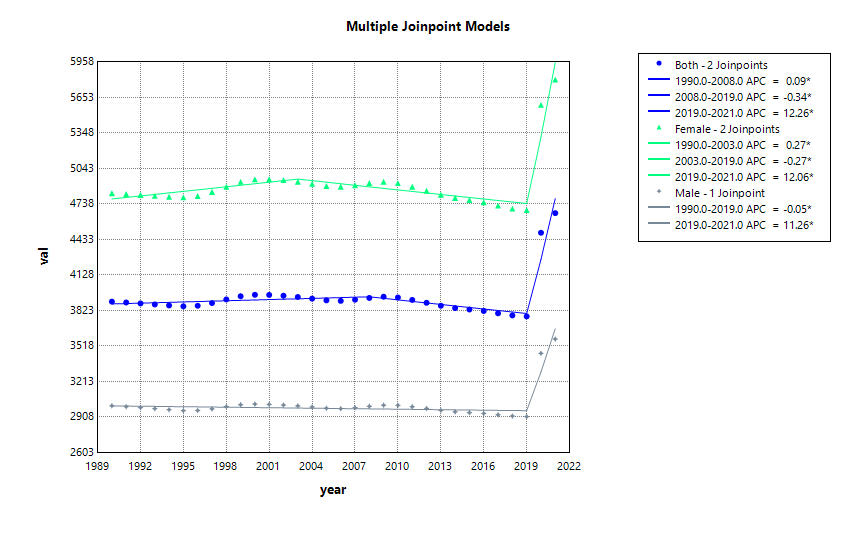 |
| **Age**  **20-24** | **C** | **D** |
|  | 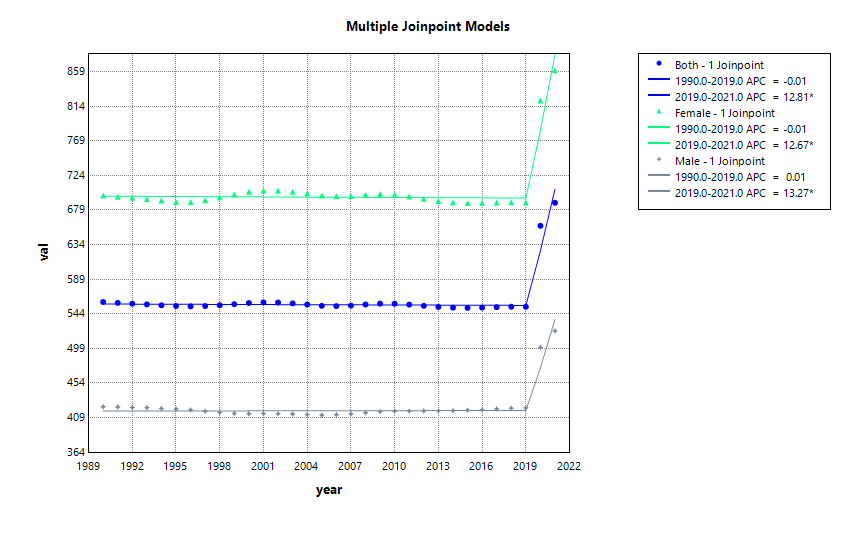 | 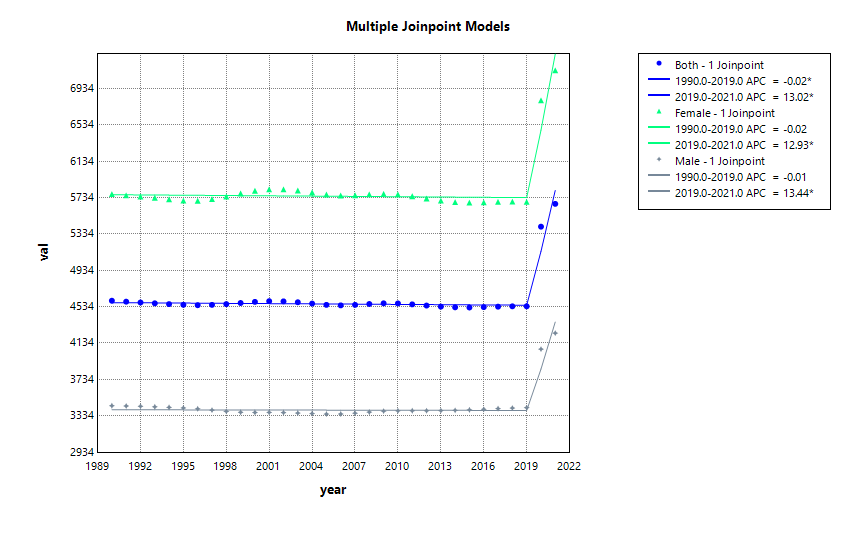 |

**Figure S2** Joinpoint regression analysis results of anxiety disorders: Disability-adjusted life years, prevalence, and years lived with disability by two age groups and sex. APC, annual percentage change; DALYs, disability-adjusted life-years; YLDs, years lived with disability; All prevalence estimates in this study refer to point prevalence; * means significance

|  | **DALYs** | **Prevalence** |
| --- | --- | --- |
| **Age**  **10-19** | **A** | **B** |
|  | 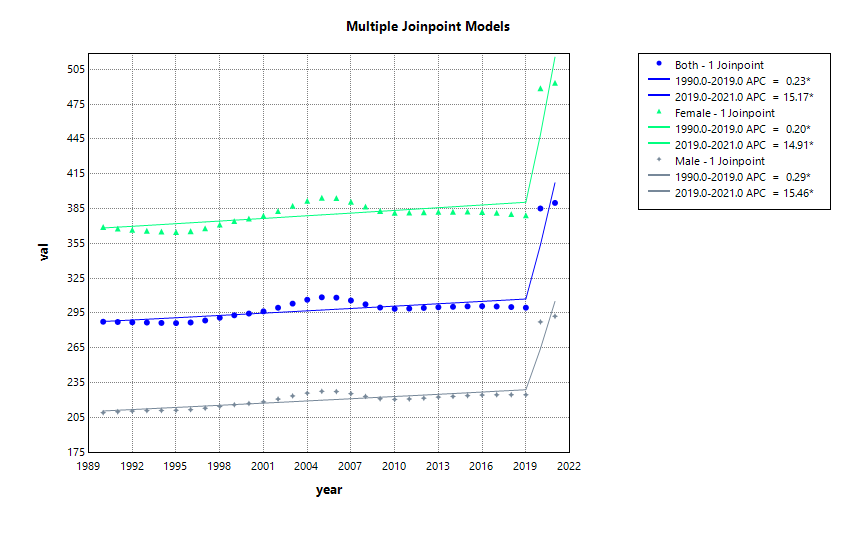 | 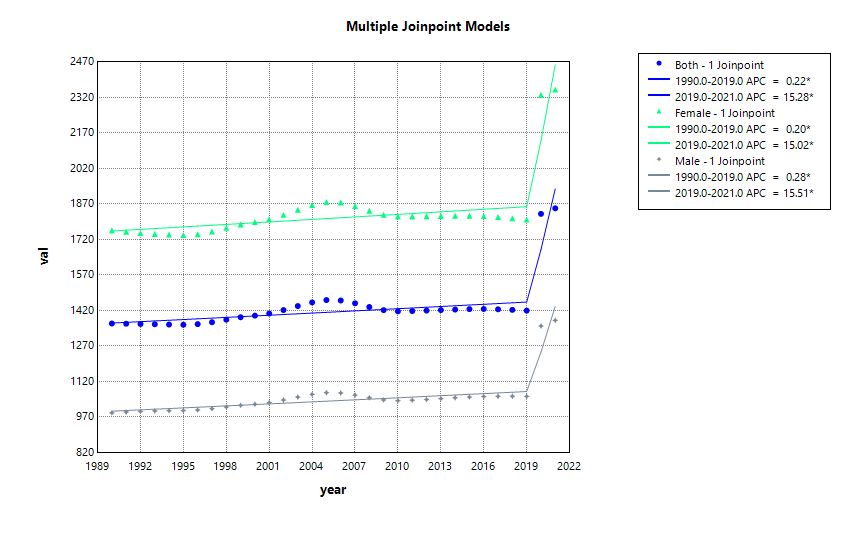 |
| **Age**  **20-24** | **C** | **D** |
|  | 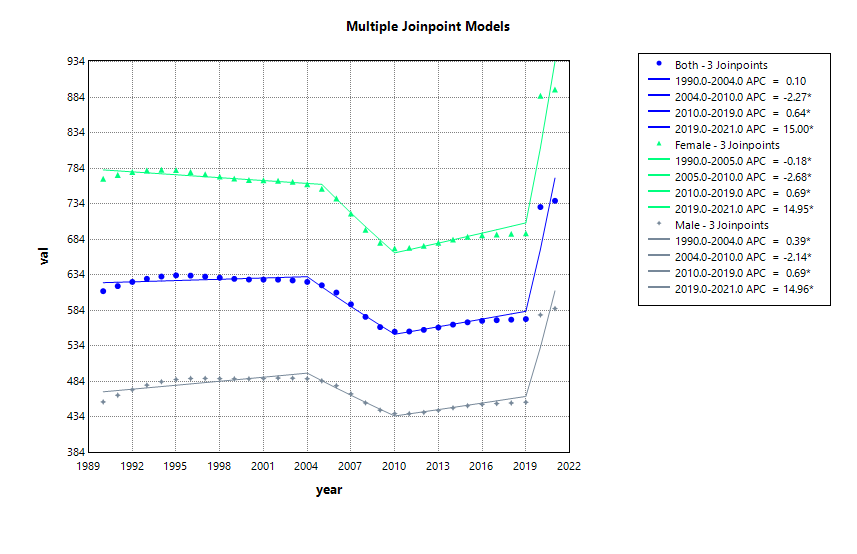 | 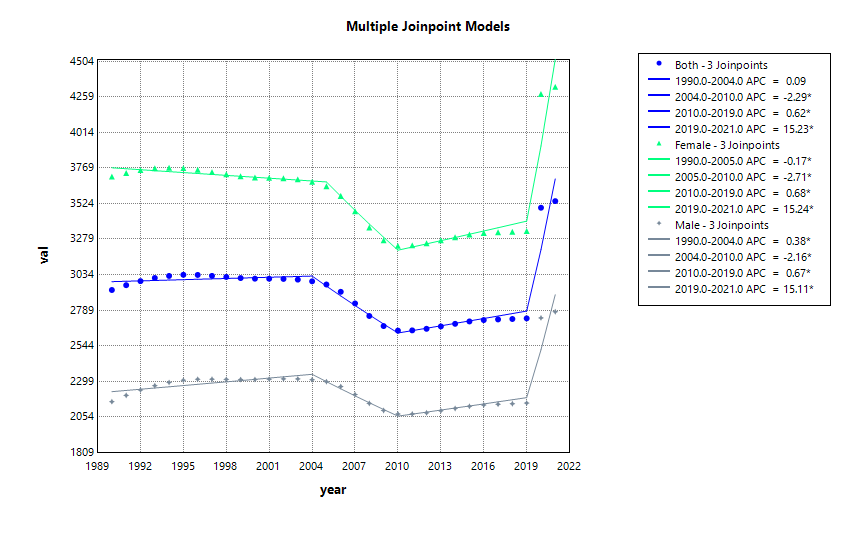 |

**Figure S3** Joinpoint regression analysis results of major depressive disorder: Disability-adjusted life years, prevalence, and years lived with disability by two age groups and sex. APC, annual percentage change; DALYs, disability-adjusted life-years; YLDs, years lived with disability; All prevalence estimates in this study refer to point prevalence; * means significance

|  | **DALYs** | **Prevalence** |
| --- | --- | --- |
| **Age**  **10-19** | **A** | **B** |
|  | 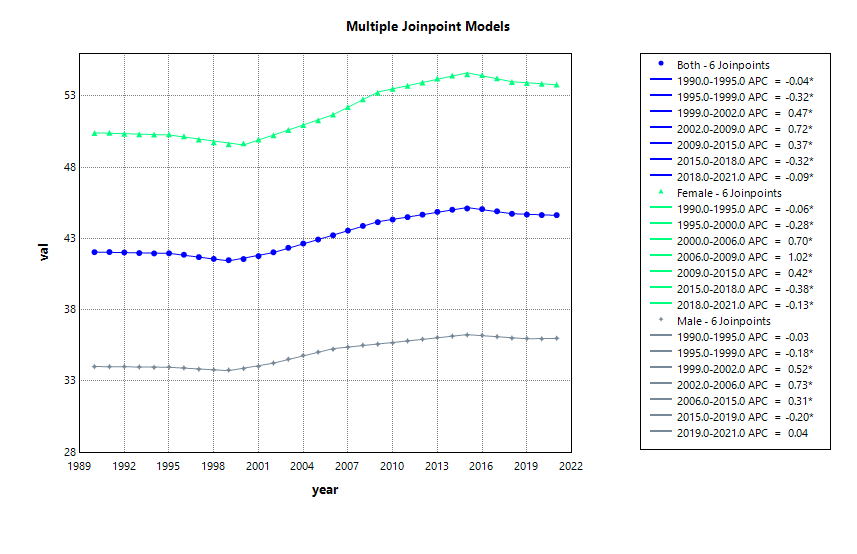 | 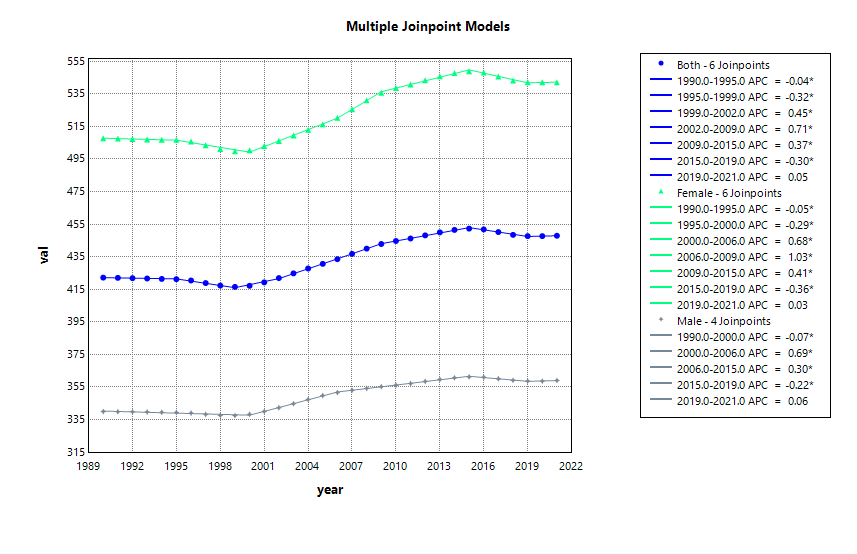 |
| **Age**  **20-24** | **C** | **D** |
|  | 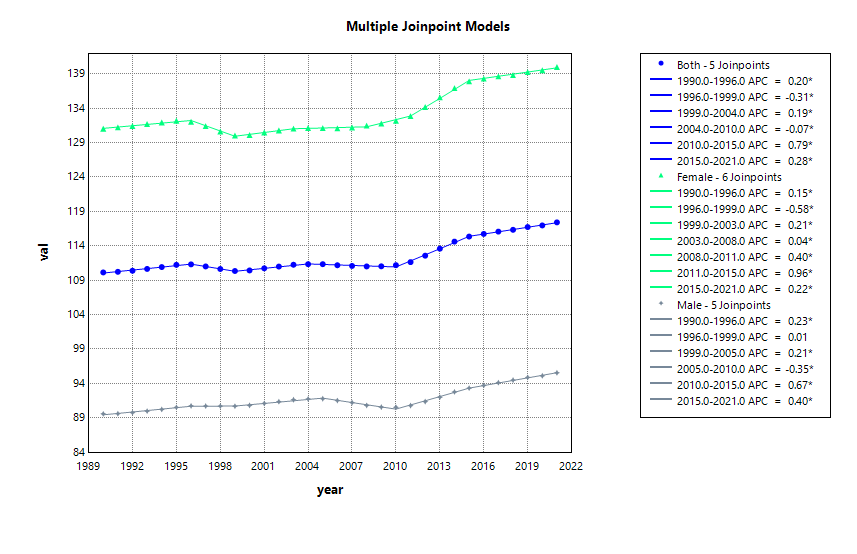 | 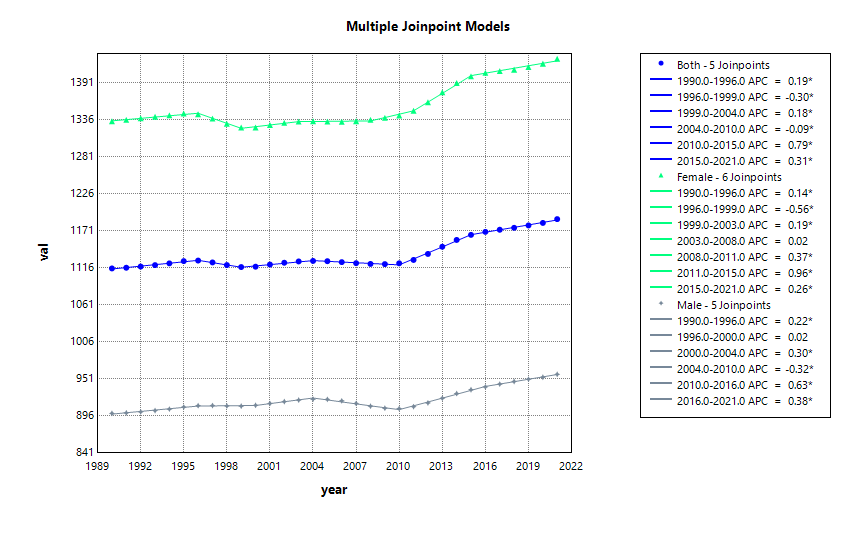 |

**Figure S4** Joinpoint regression analysis results of dysthymia: Disability-adjusted life years, prevalence, and years lived with disability by two age groups and sex. APC, annual percentage change; DALYs, disability-adjusted life-years; YLDs, years lived with disability; All prevalence estimates in this study refer to point prevalence; * means significance

|  | **DALYs** | **Prevalence** |
| --- | --- | --- |
| **Age**  **10-19** | **A** | **B** |
|  | 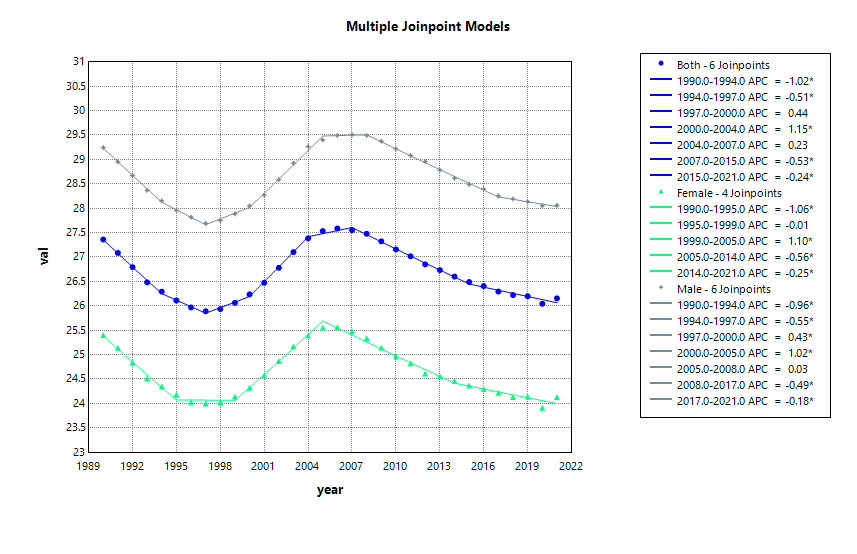 | 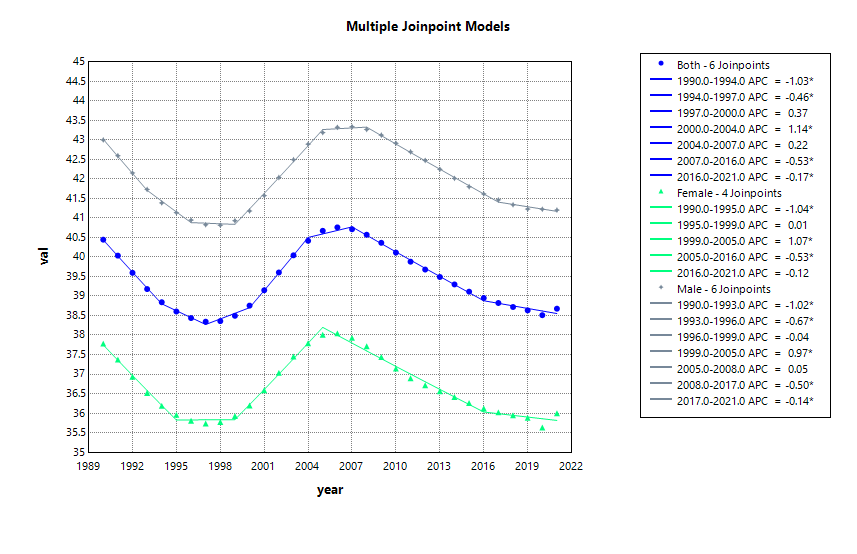 |
| **Age**  **20-24** | **C** | **D** |
|  | 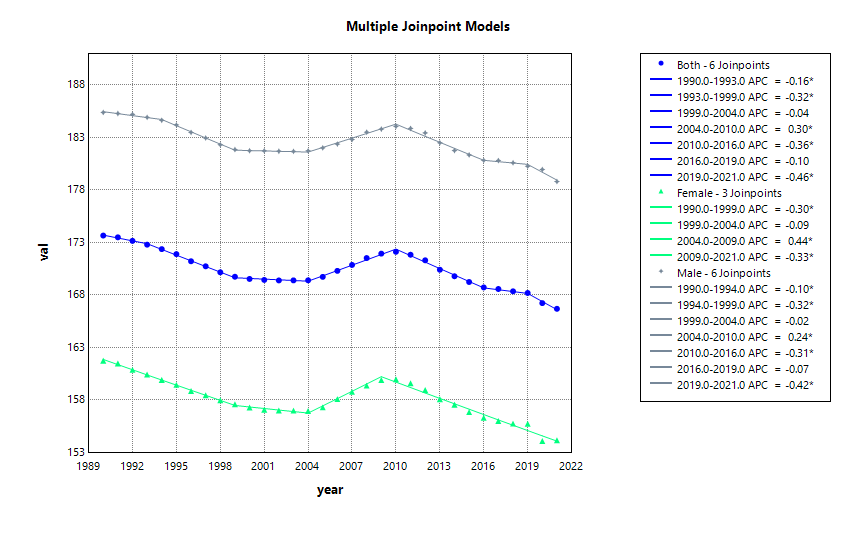 | 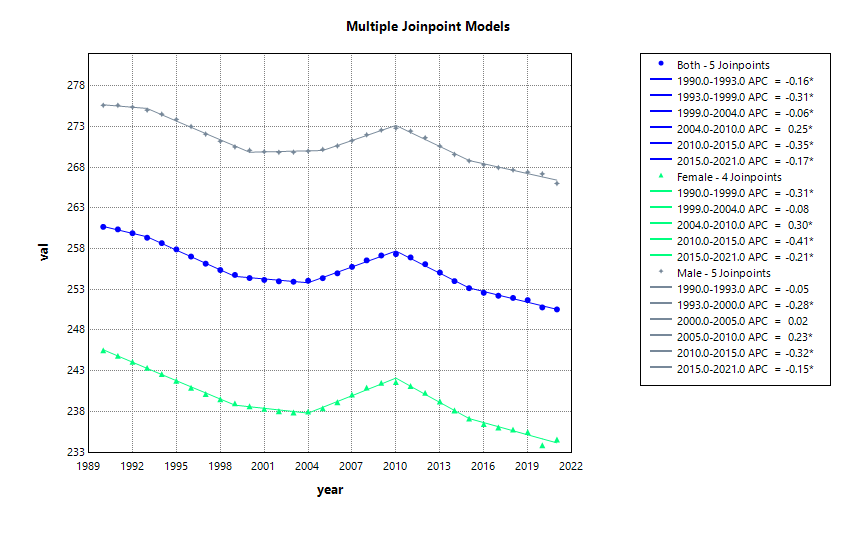 |

**Figure S5** Joinpoint regression analysis results of schizophrenia: Disability-adjusted life years, prevalence, and years lived with disability by two age groups and sex. APC, annual percentage change; DALYs, disability-adjusted life-years; YLDs, years lived with disability; All prevalence estimates in this study refer to point prevalence; * means significance

|  | **DALYs** | **Prevalence** |
| --- | --- | --- |
| **Age**  **10-19** | **A** | **B** |
|  | 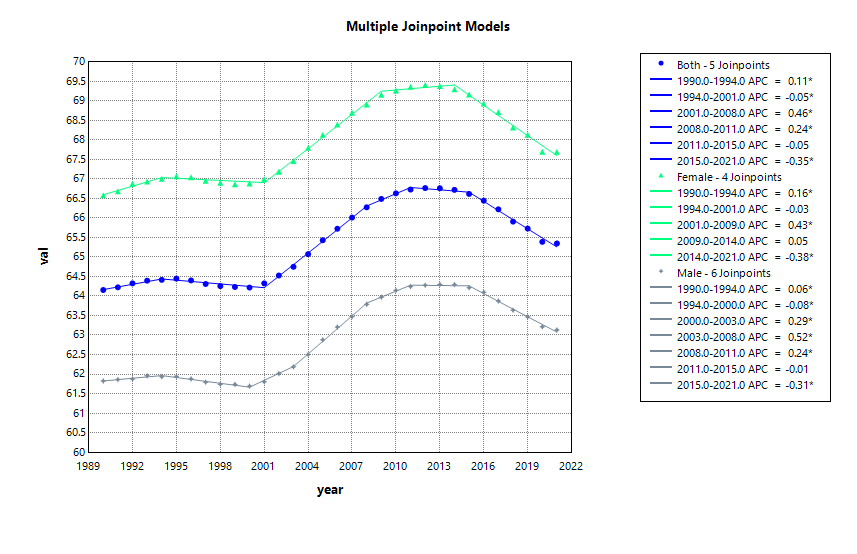 | 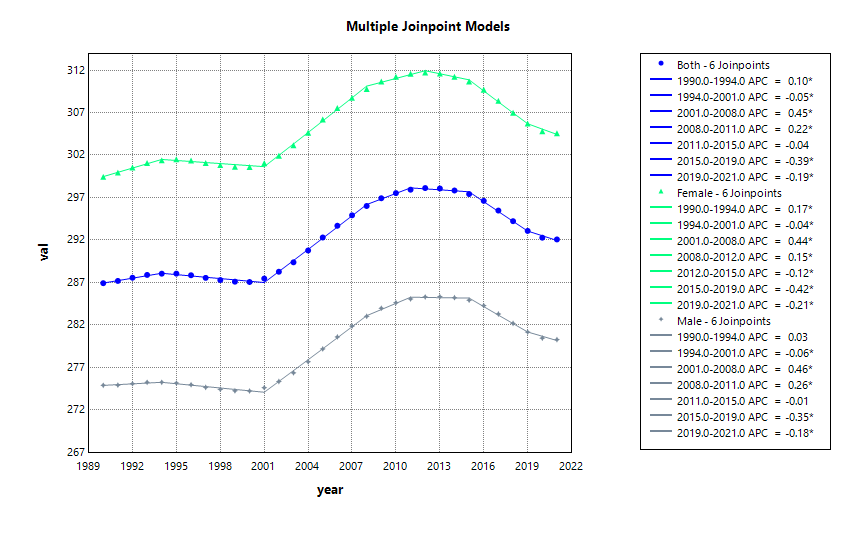 |
| **Age**  **20-24** | **C** | **D** |
|  | 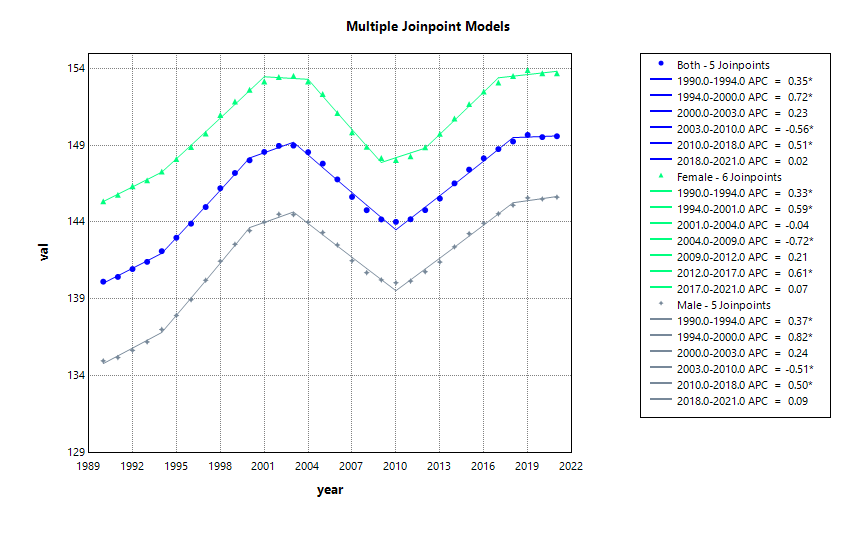 | 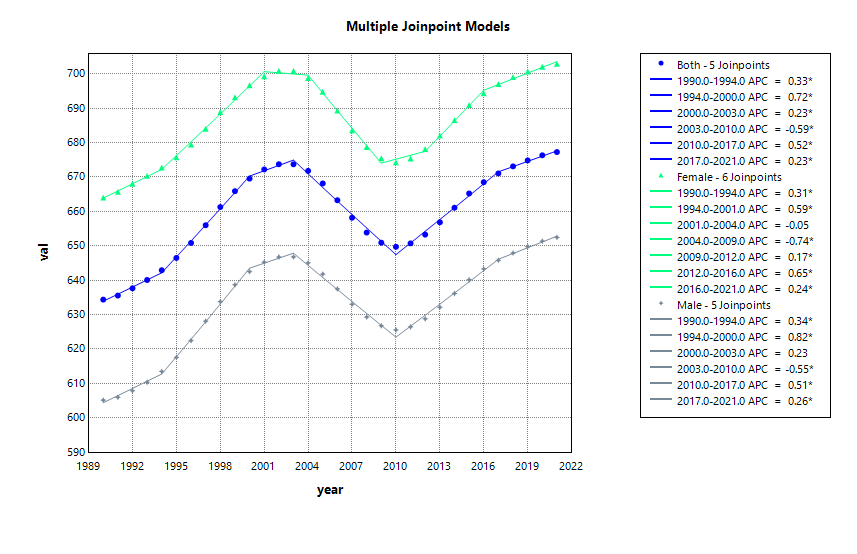 |

**Figure S6** Joinpoint regression analysis results of bipolar disorder: Disability-adjusted life years, prevalence, and years lived with disability by two age groups and sex. APC, annual percentage change; DALYs, disability-adjusted life-years; YLDs, years lived with disability; All prevalence estimates in this study refer to point prevalence; * means significance

|  | **DALYs** | **Prevalence** |
| --- | --- | --- |
| **Age**  **10-19** | **A** | **B** |
|  | 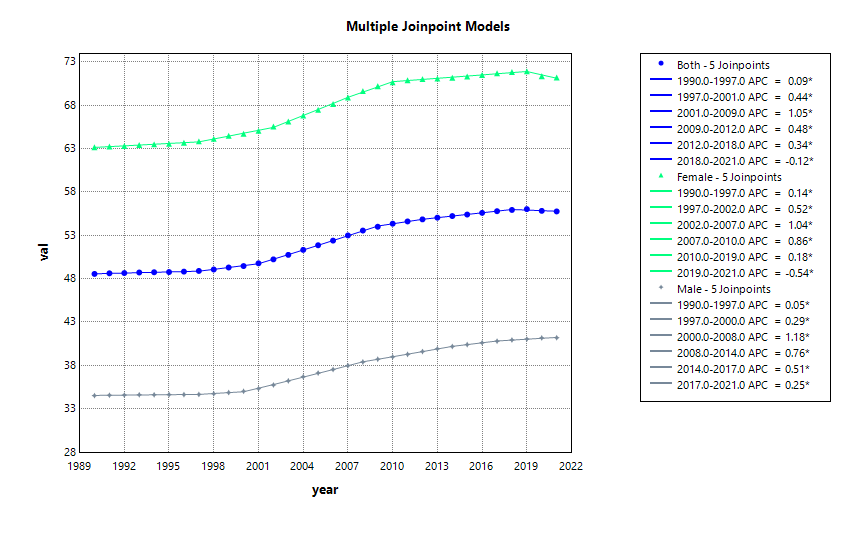 | 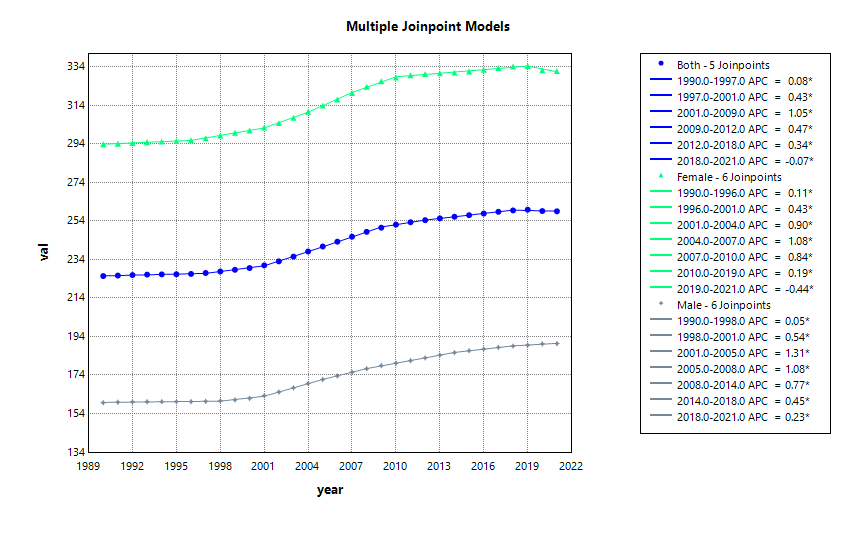 |
| **Age**  **20-24** | **C** | **D** |
|  | 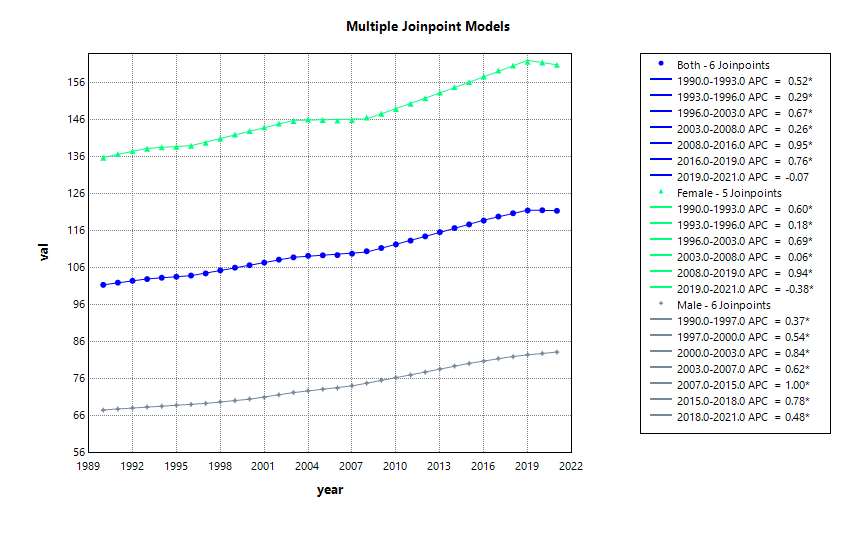 | 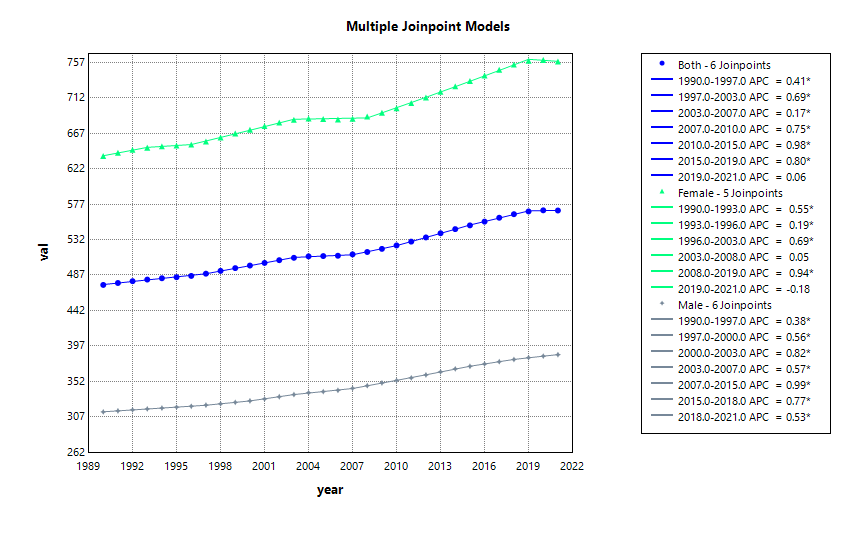 |

**Figure S7** Joinpoint regression analysis results of eating disorders: Disability-adjusted life years, prevalence, and years lived with disability by two age groups and sex. APC, annual percentage change; DALYs, disability-adjusted life-years; YLDs, years lived with disability; All prevalence estimates in this study refer to point prevalence; * means significance

|  | **DALYs** | **Prevalence** |
| --- | --- | --- |
| **Age**  **10-19** | **A** | **B** |
|  | 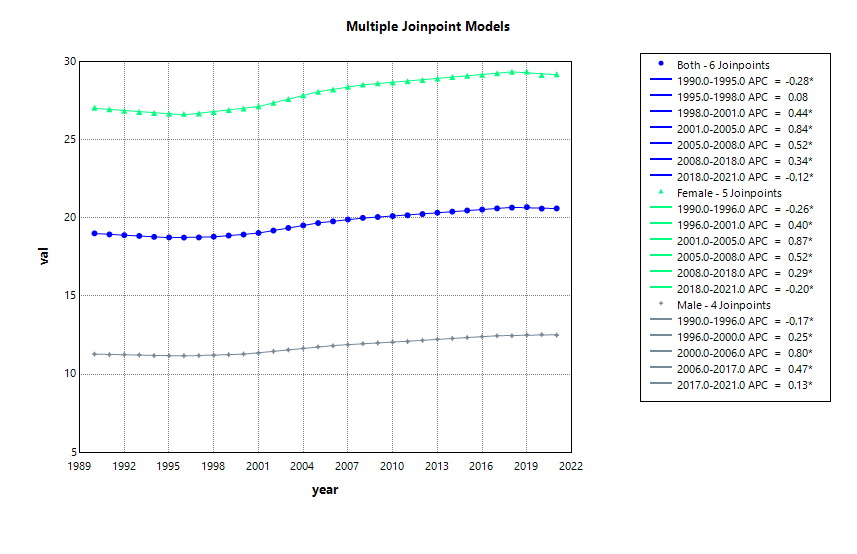 | 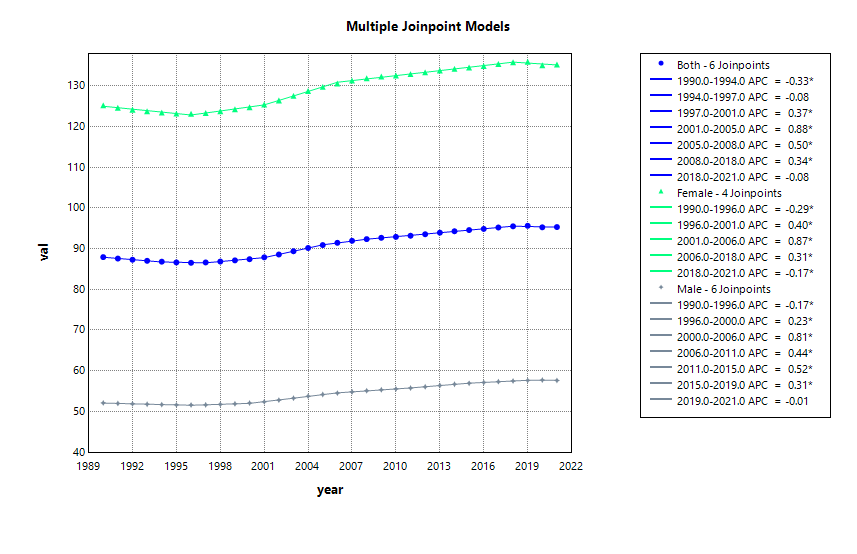 |
| **Age**  **20-24** | **C** | **D** |
|  | 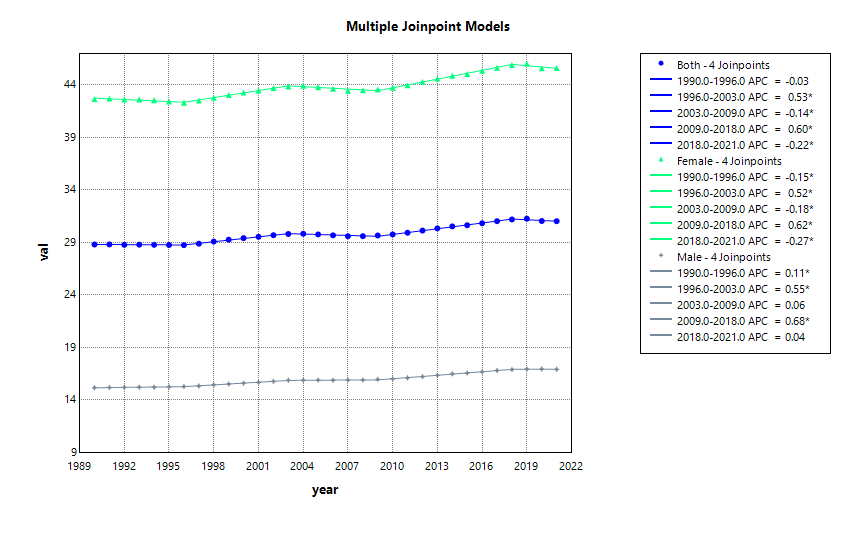 | 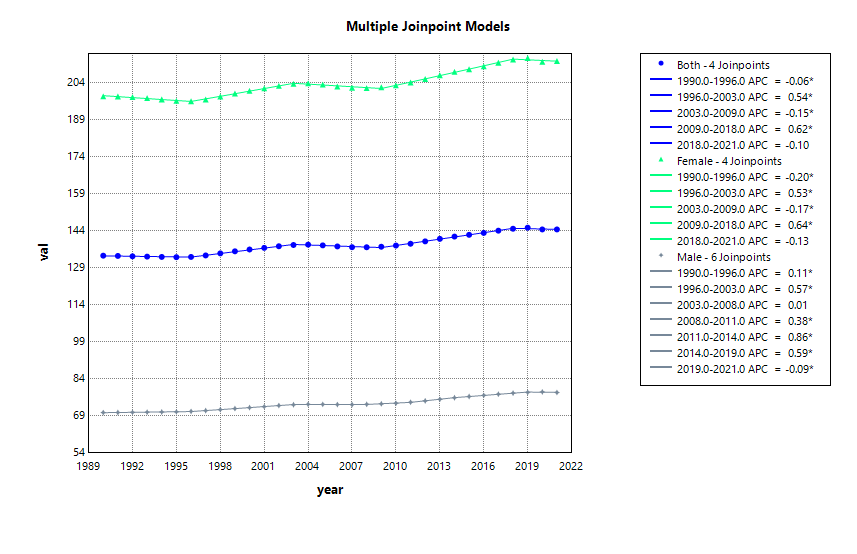 |

**Figure S8** Joinpoint regression analysis results of anorexia nervosa: Disability-adjusted life years, prevalence, and years lived with disability by two age groups and sex. APC, annual percentage change; DALYs, disability-adjusted life-years; YLDs, years lived with disability; All prevalence estimates in this study refer to point prevalence; * means significance

|  | **DALYs** | **Prevalence** |
| --- | --- | --- |
| **Age**  **10-19** | **A** | **B** |
|  | 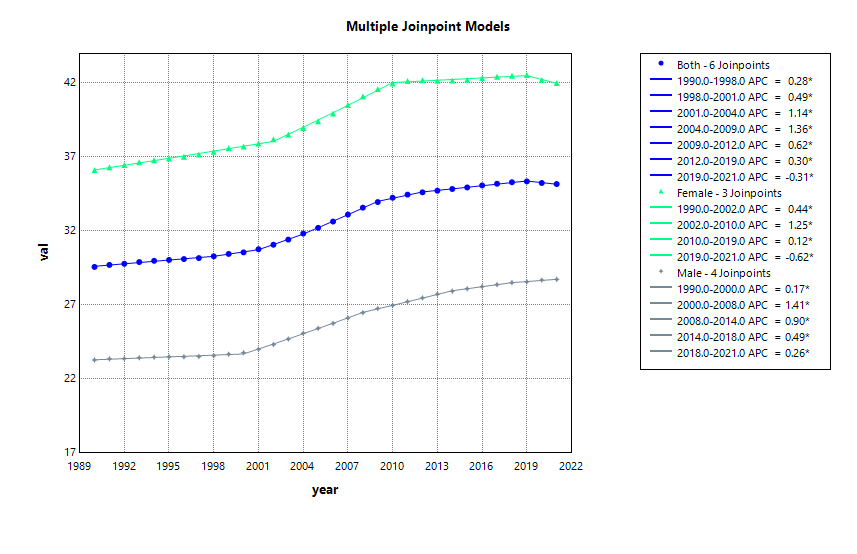 | 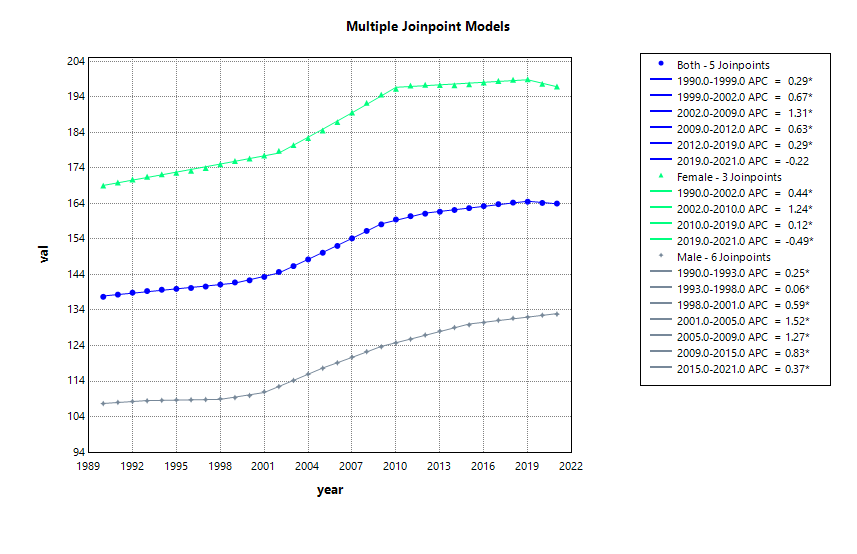 |
| **Age**  **20-24** | **C** | **D** |
|  | 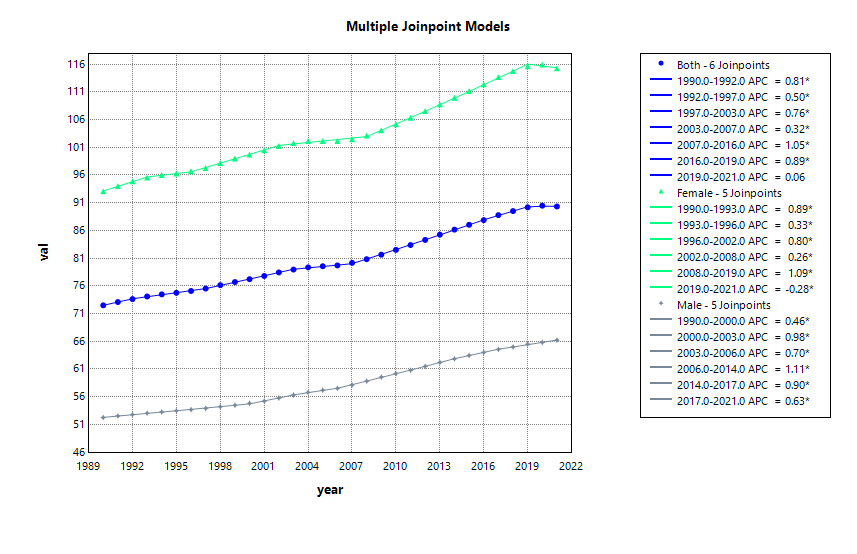 | 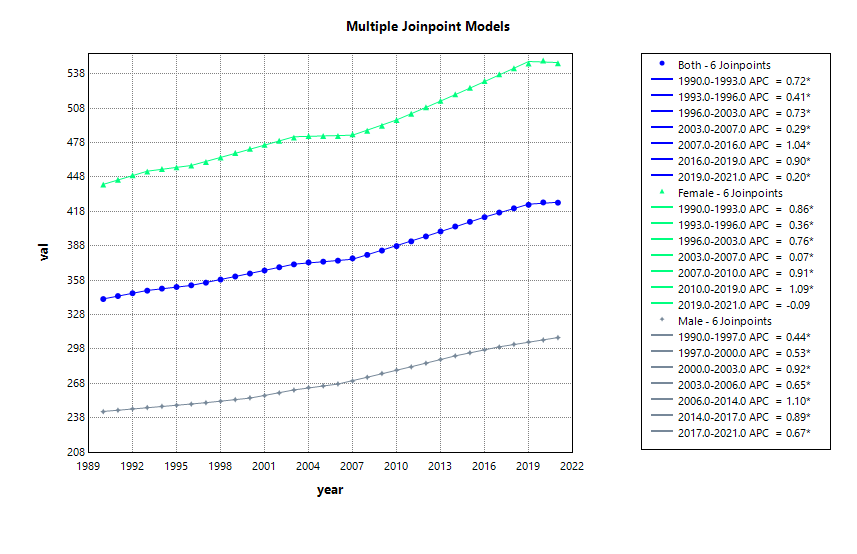 |

**Figure S9** Joinpoint regression analysis results of bulimia nervosa: Disability-adjusted life years, prevalence, and years lived with disability by two age groups and sex. APC, annual percentage change; DALYs, disability-adjusted life-years; YLDs, years lived with disability; All prevalence estimates in this study refer to point prevalence; * means significance

|  | **DALYs** | **Prevalence** |
| --- | --- | --- |
| **Age**  **10-19** | **A** | **B** |
|  | 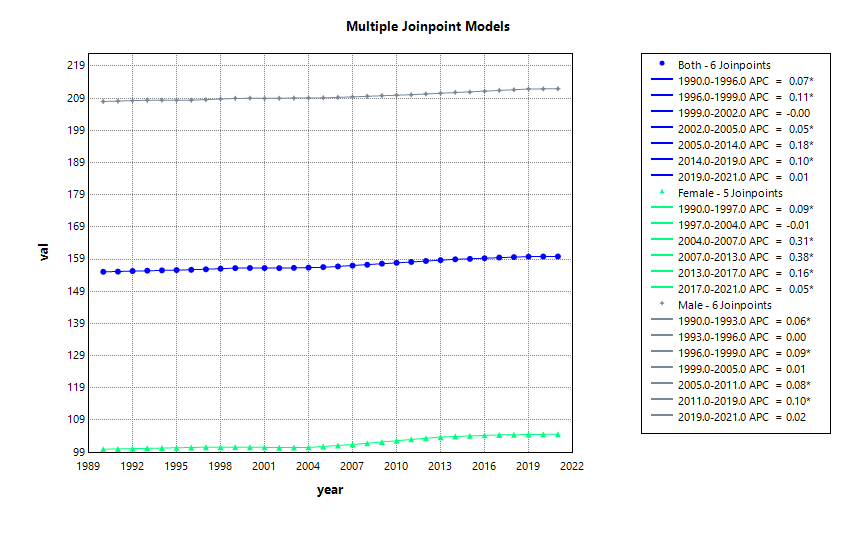 | 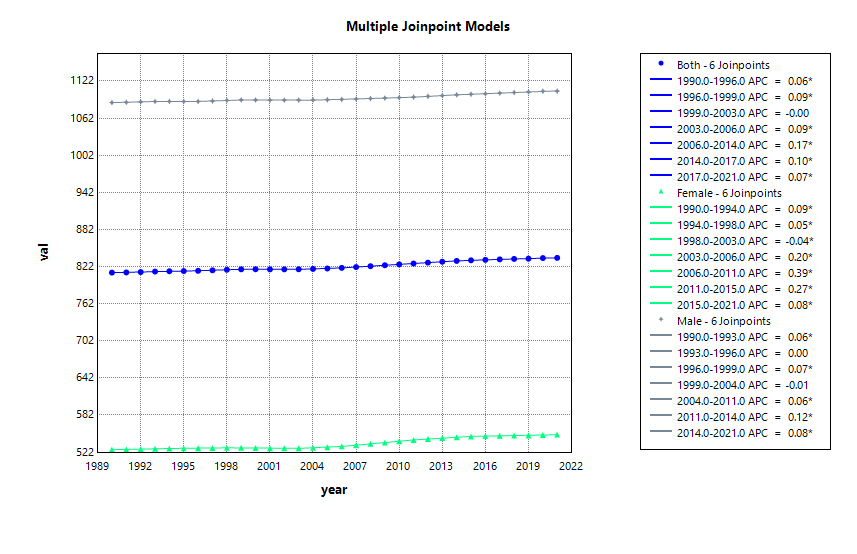 |
| **Age**  **20-24** | **C** | **D** |
|  | 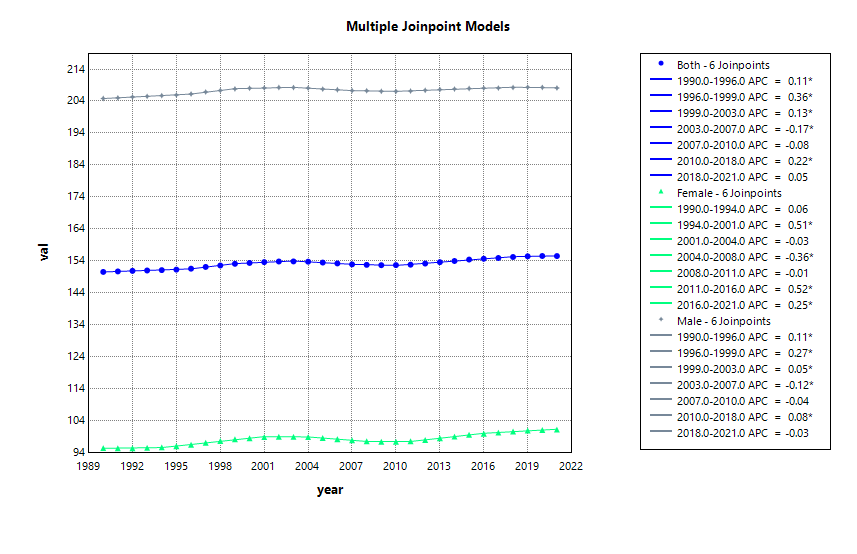 | 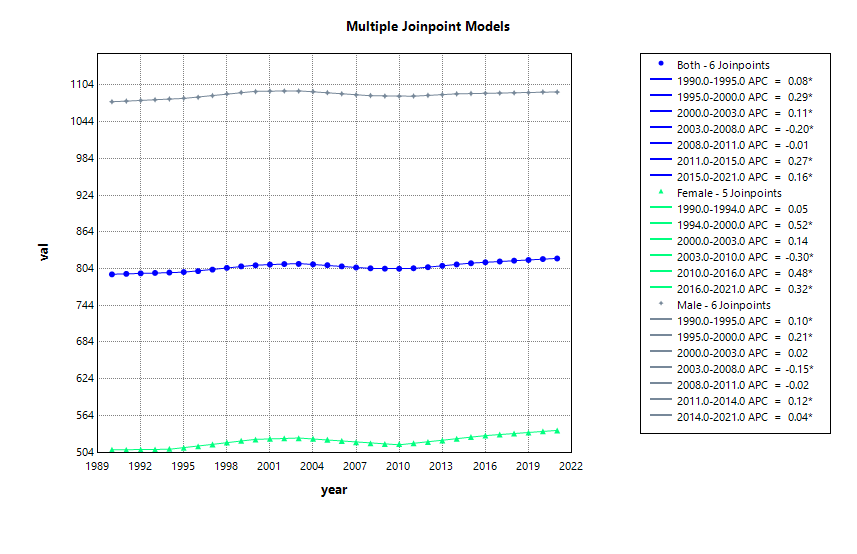 |

**Figure S10** Joinpoint regression analysis results of autism spectrum disorders: Disability-adjusted life years, prevalence, and years lived with disability by two age groups and sex. APC, annual percentage change; DALYs, disability-adjusted life-years; YLDs, years lived with disability; All prevalence estimates in this study refer to point prevalence; * means significance

|  | **DALYs** | **Prevalence** |
| --- | --- | --- |
| **Age**  **10-19** | **A** | **B** |
|  | 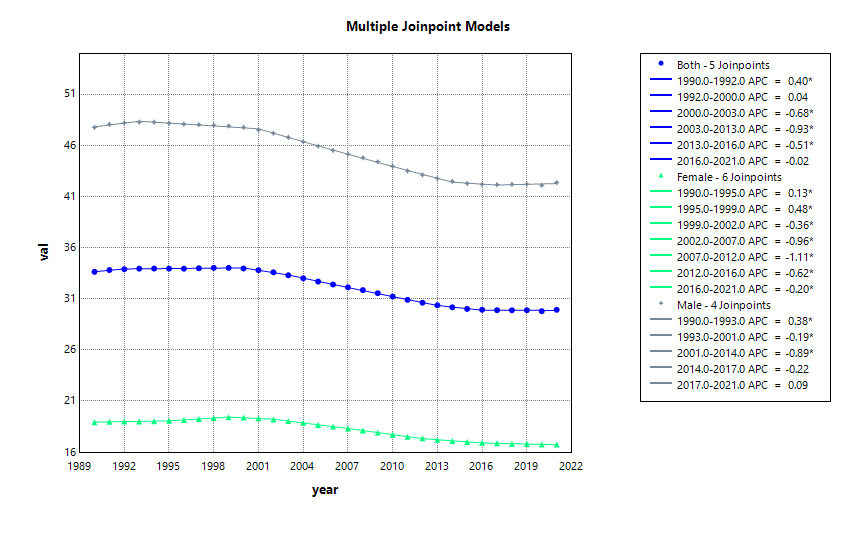 | 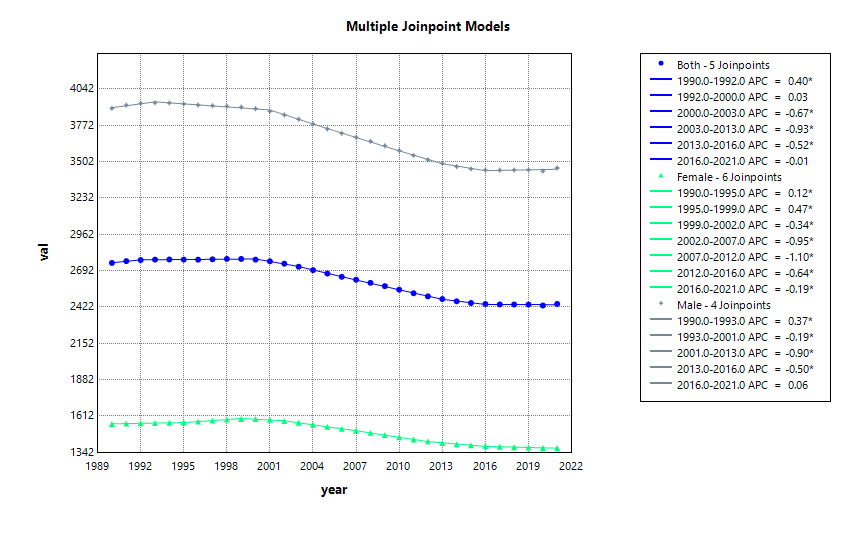 |
| **Age**  **20-24** | **C** | **D** |
|  | 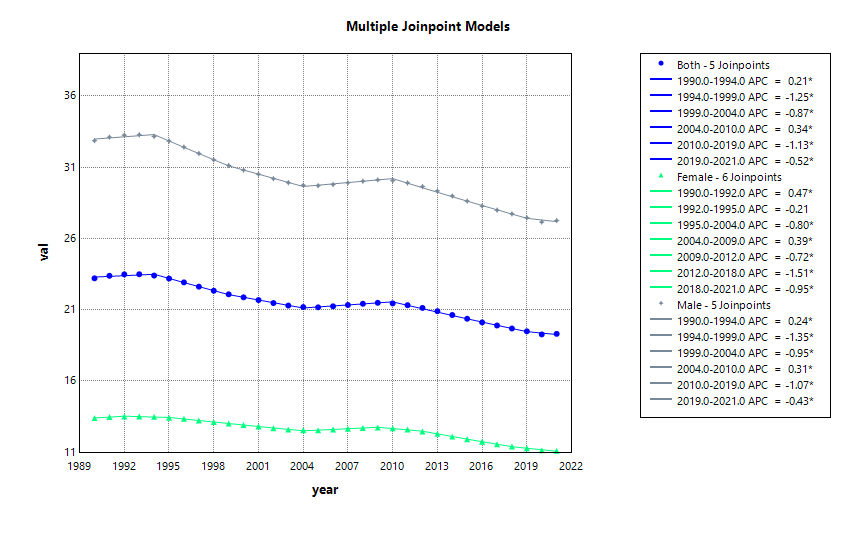 | 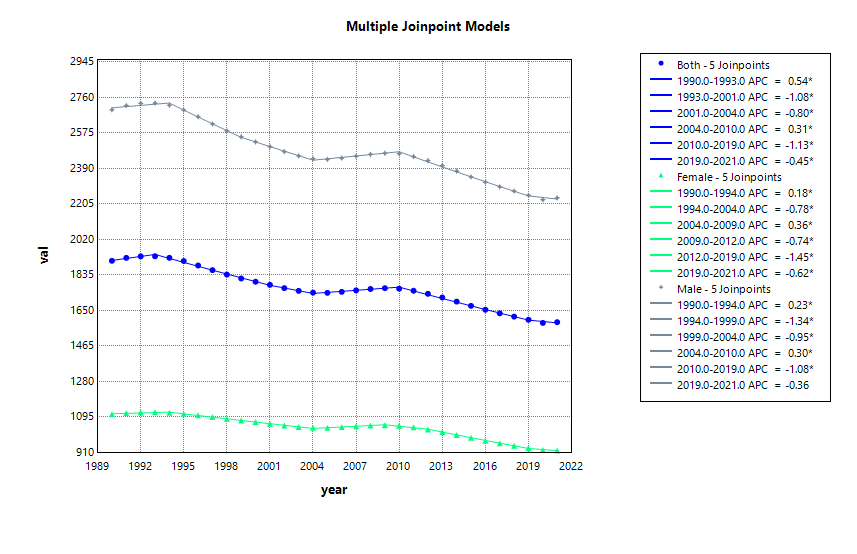 |

**Figure S11** Joinpoint regression analysis results of attention-deficit hyperactivity disorder: Disability-adjusted life years, prevalence, and years lived with disability by two age groups and sex. APC, annual percentage change; DALYs, disability-adjusted life-years; YLDs, years lived with disability; All prevalence estimates in this study refer to point prevalence; * means significance

|  | **DALYs** | **Prevalence** |
| --- | --- | --- |
| **Age**  **10-19** | **A** | **B** |
|  | 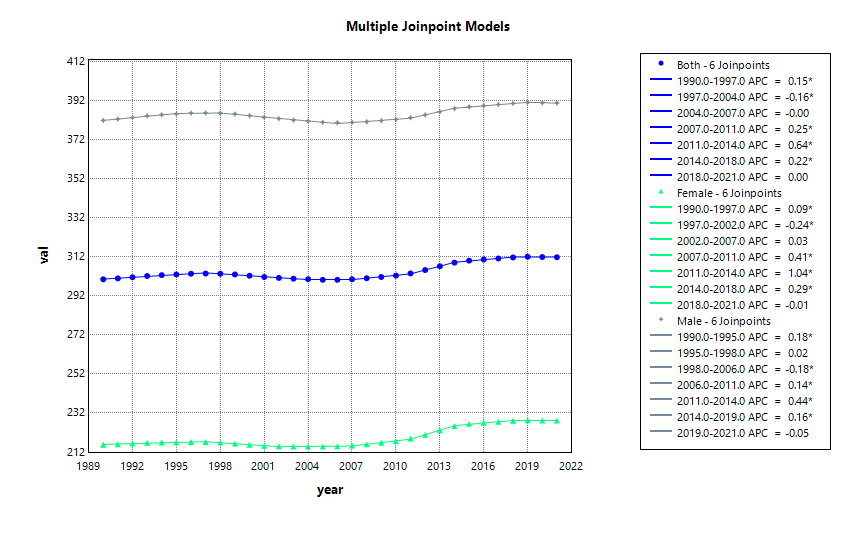 | 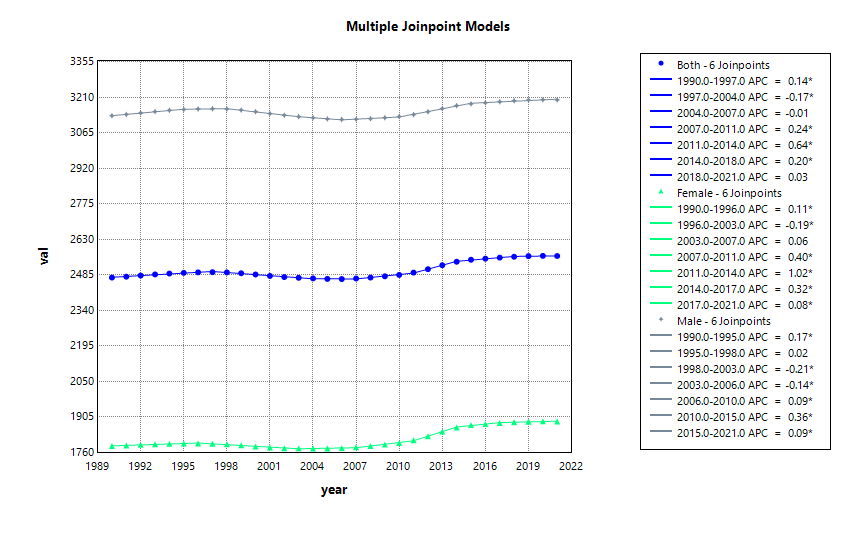 |
| **Age**  **20-24** | **C** | **D** |
|  | 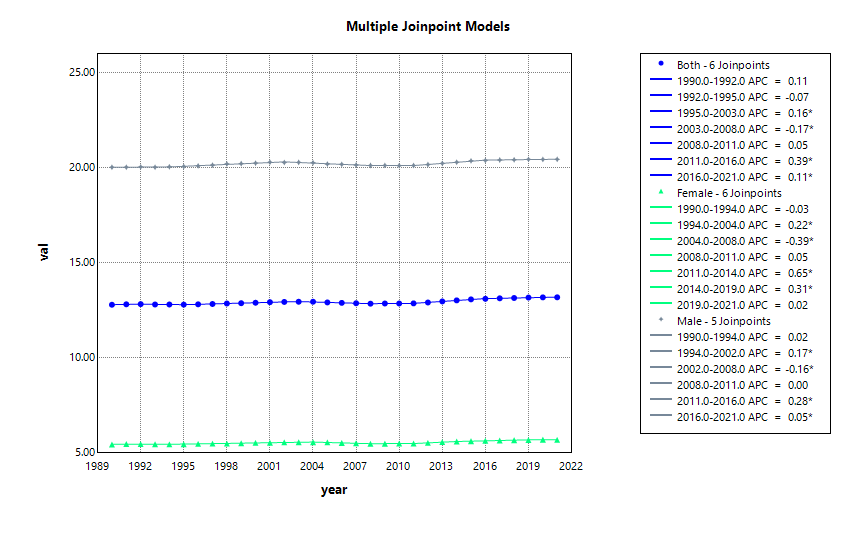 | 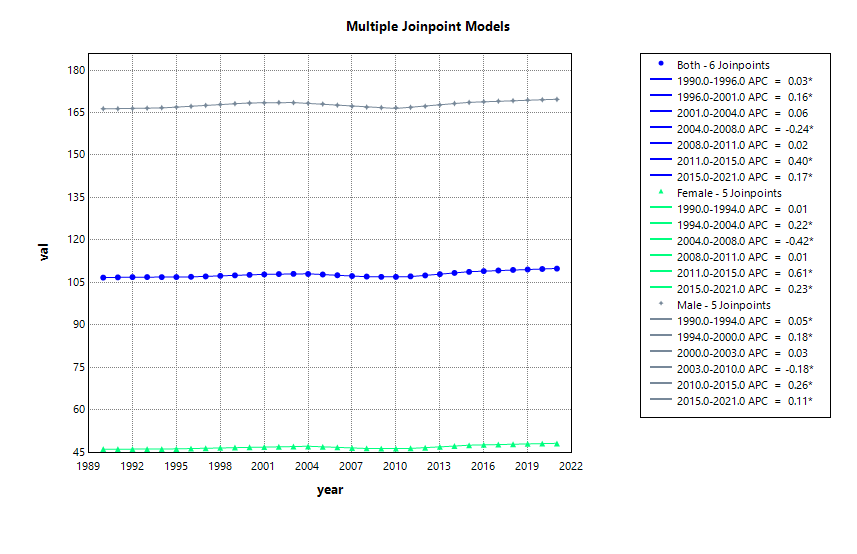 |

**Figure S12** Joinpoint regression analysis results of conduct disorder: Disability-adjusted life years, prevalence, and years lived with disability by two age groups and sex. APC, annual percentage change; DALYs, disability-adjusted life-years; YLDs, years lived with disability; All prevalence estimates in this study refer to point prevalence; * means significance

|  | **DALYs** | **Prevalence** |
| --- | --- | --- |
| **Age**  **10-19** | **A** | **B** |
|  | 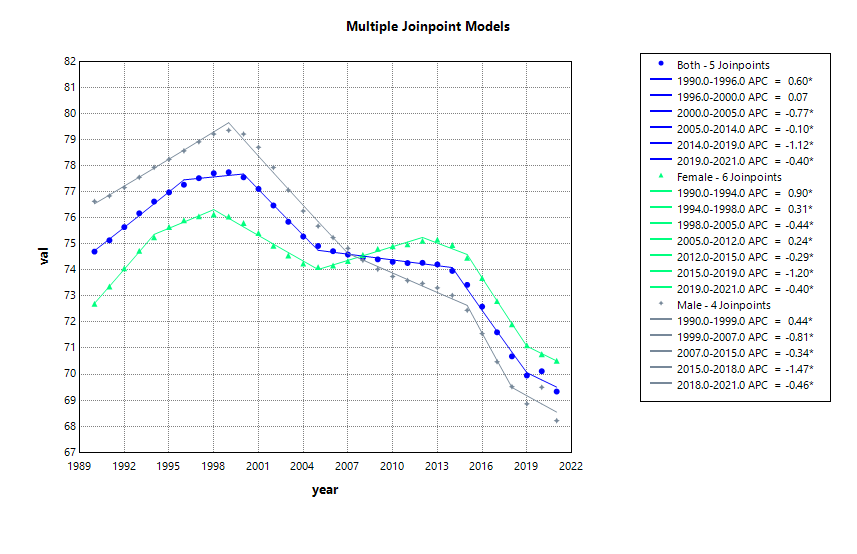 | 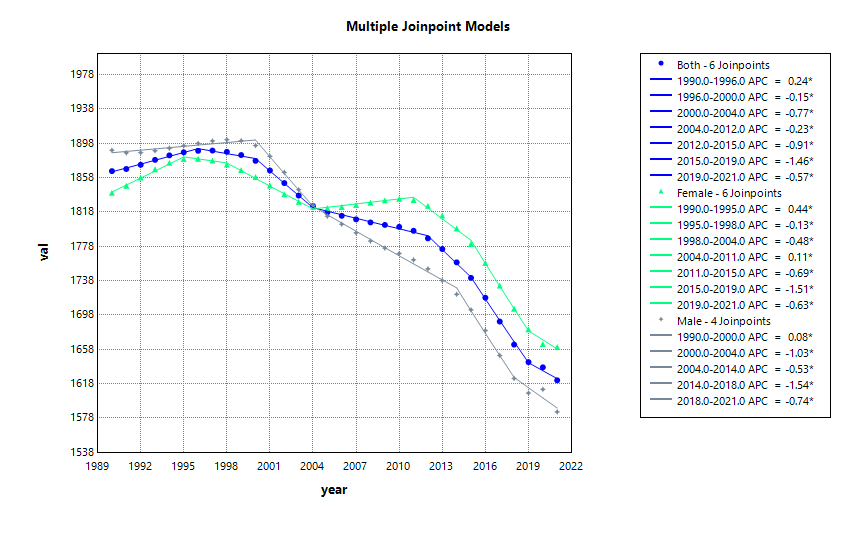 |
| **Age**  **20-24** | **C** | **D** |
|  | 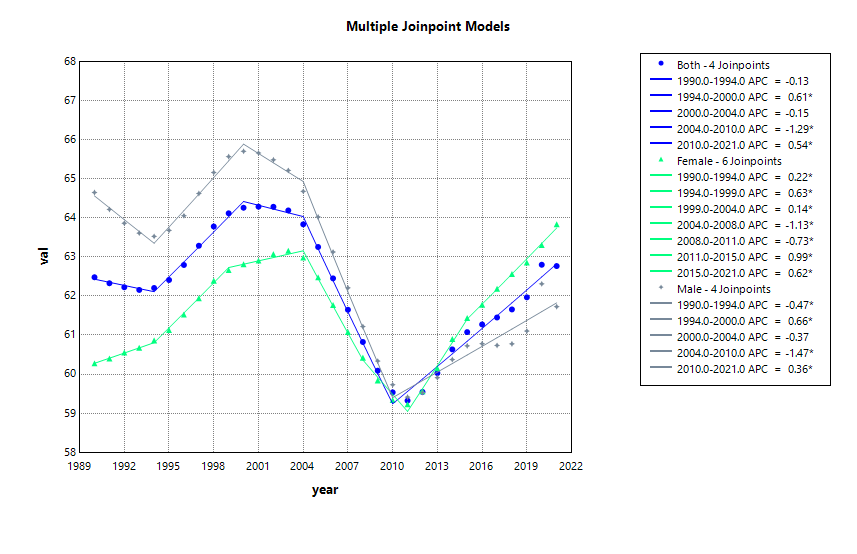 | 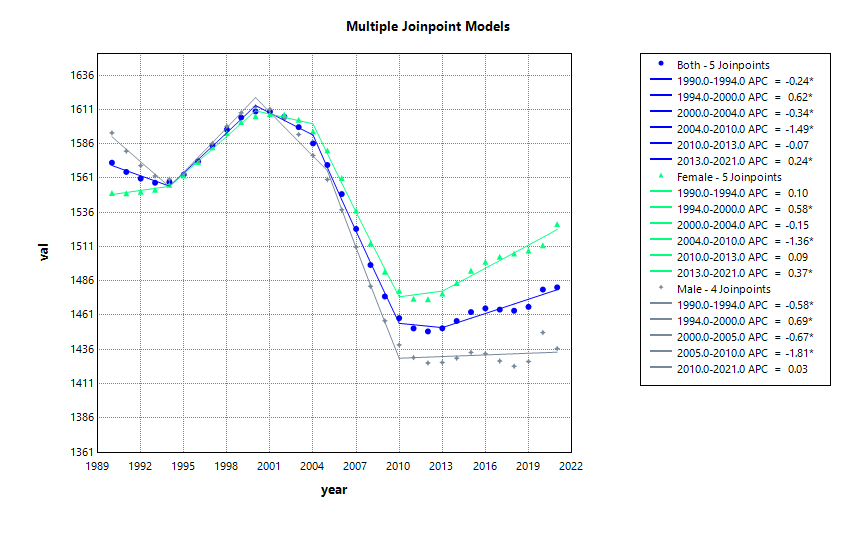 |

**Figure S13** Joinpoint regression analysis results of idiopathic developmental intellectual disability: Disability-adjusted life years, prevalence, and years lived with disability by two age groups and sex. APC, annual percentage change; DALYs, disability-adjusted life-years; YLDs, years lived with disability; All prevalence estimates in this study refer to point prevalence; * means significance

|  | **DALYs** | **Prevalence** |
| --- | --- | --- |
| **Age**  **10-19** | **A** | **B** |
|  | 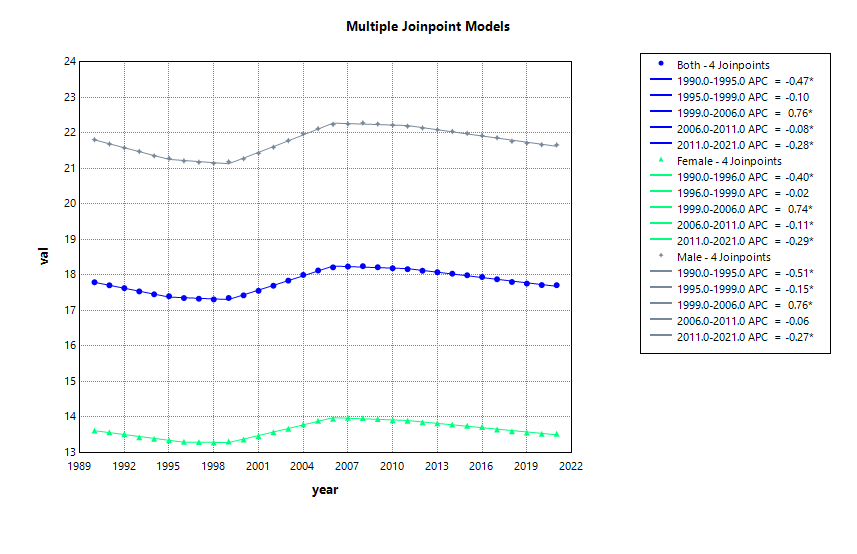 | 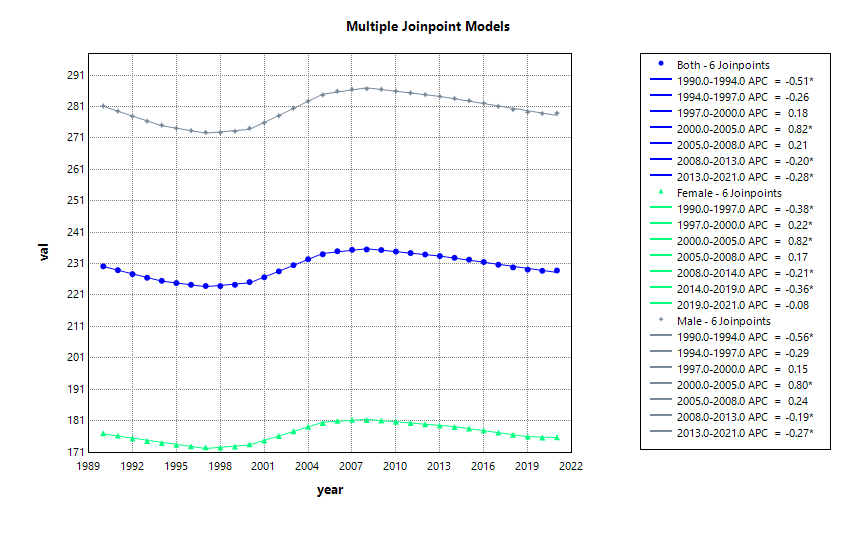 |
| **Age**  **20-24** | **C** | **D** |
|  | 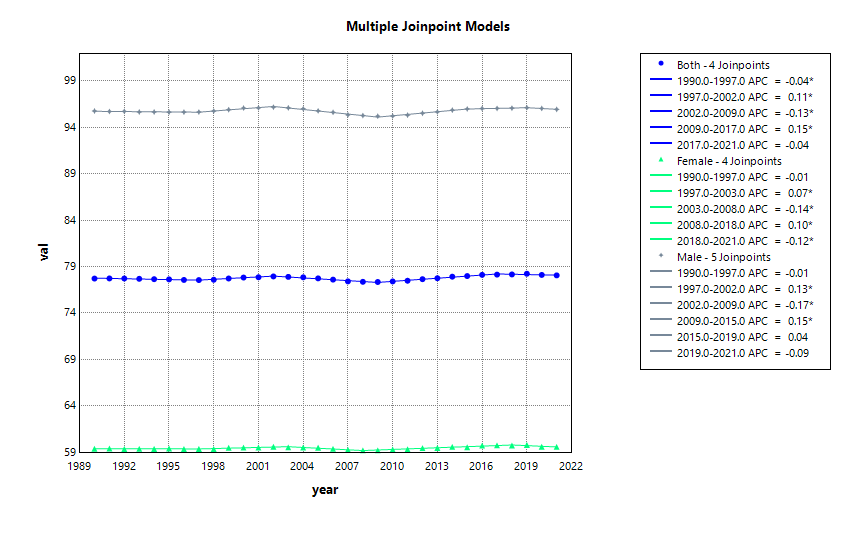 | 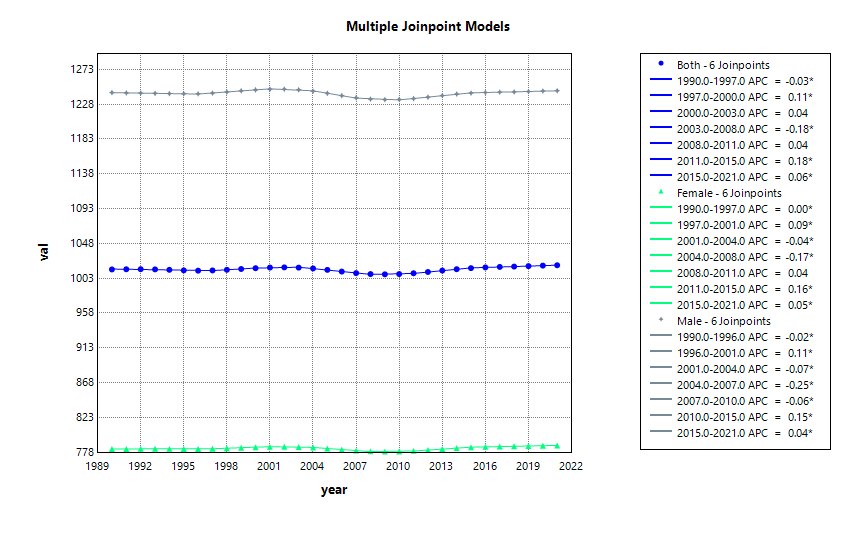 |

**Figure S14** Joinpoint regression analysis results of other mental disorders: Disability-adjusted life years, prevalence, and years lived with disability by two age groups and sex. APC, annual percentage change; DALYs, disability-adjusted life-years; YLDs, years lived with disability; All prevalence estimates in this study refer to point prevalence; * means significance

|  | **DALYs** | **Prevalence** |
| --- | --- | --- |
| **Age**  **10-19** | **A** | **B** |
|  | 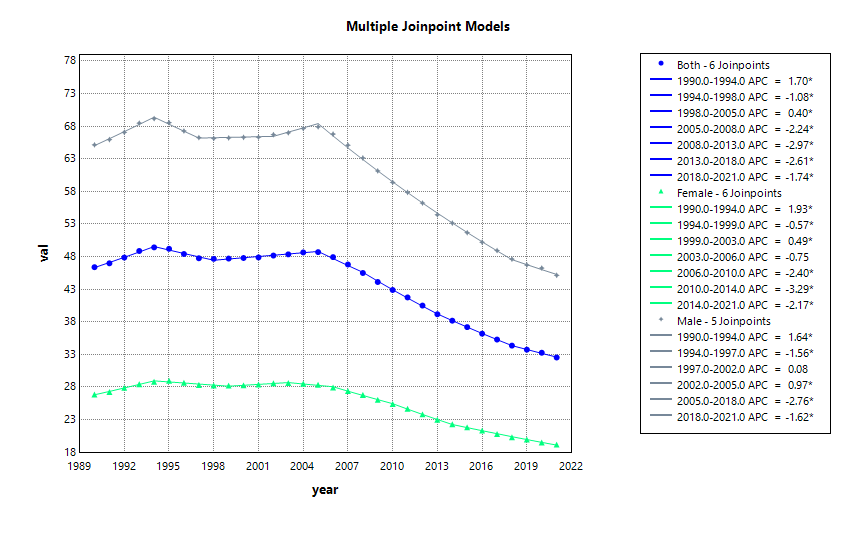 | 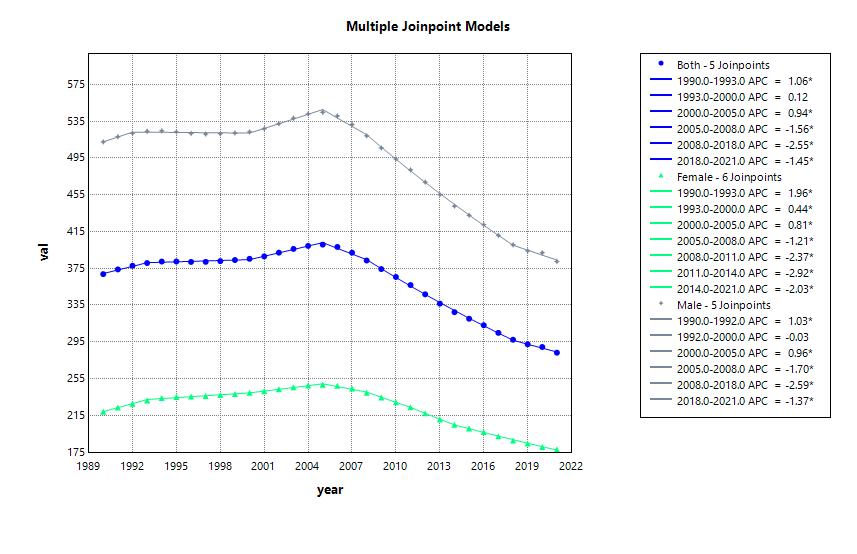 |
| **Age**  **20-24** | **C** | **D** |
|  | 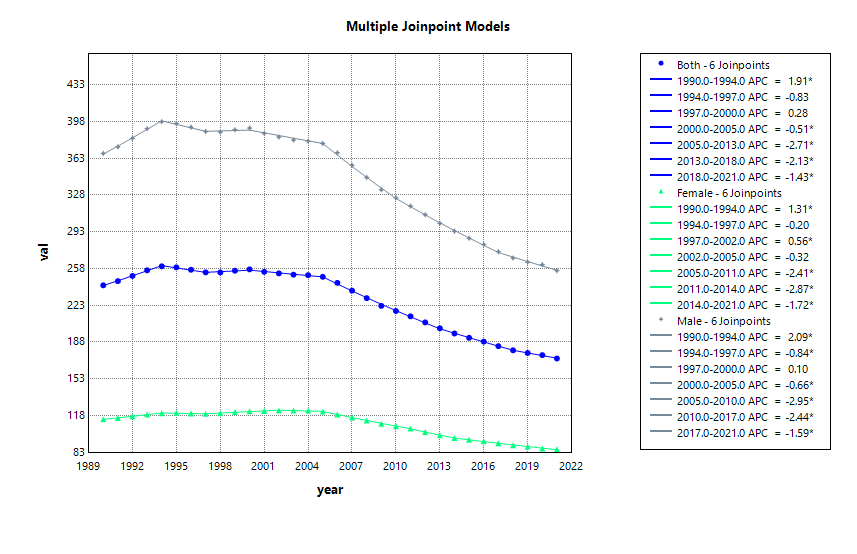 | 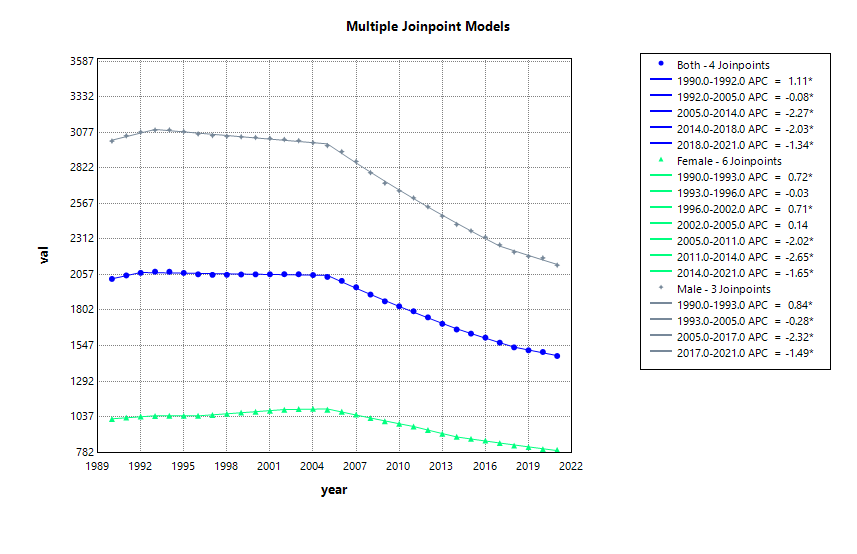 |

**Figure S15** Joinpoint regression analysis results of alcohol use disorder: Disability-adjusted life years, prevalence, and years lived with disability by two age groups and sex. APC, annual percentage change; DALYs, disability-adjusted life-years; YLDs, years lived with disability; All prevalence estimates in this study refer to point prevalence; * means significance

|  | **DALYs** | **Prevalence** |
| --- | --- | --- |
| **Age**  **10-19** | **A** | **B** |
|  | 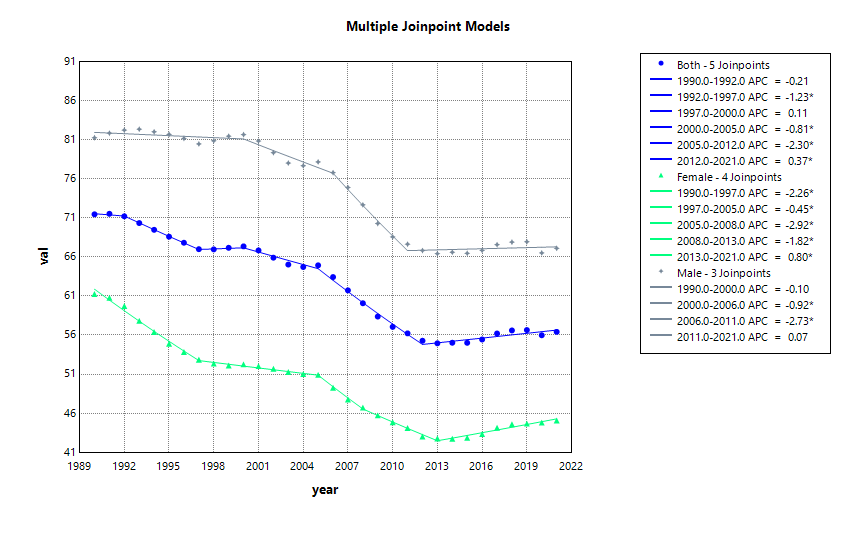 | 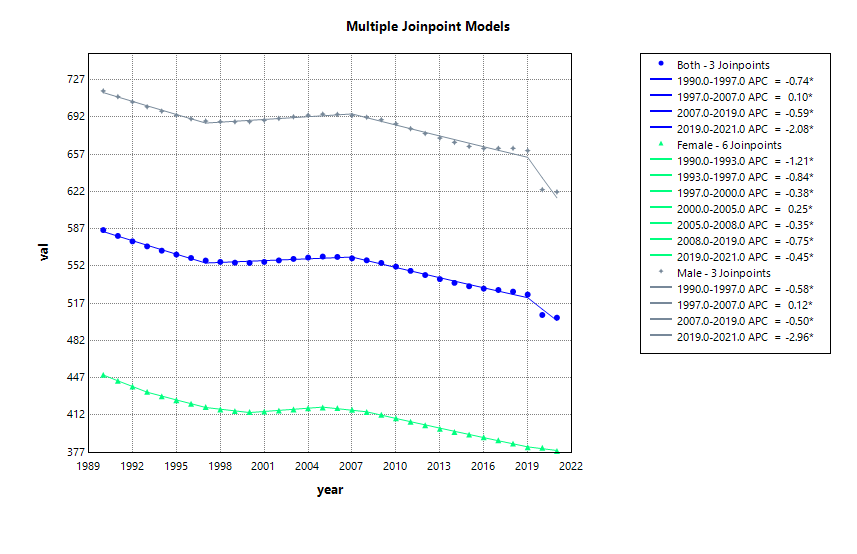 |
| **Age**  **20-24** | **C** | **D** |
|  | 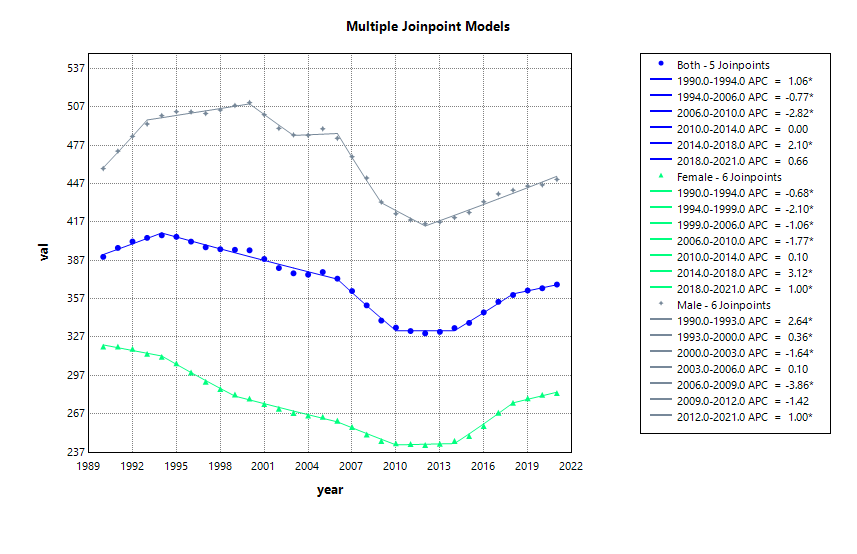 | 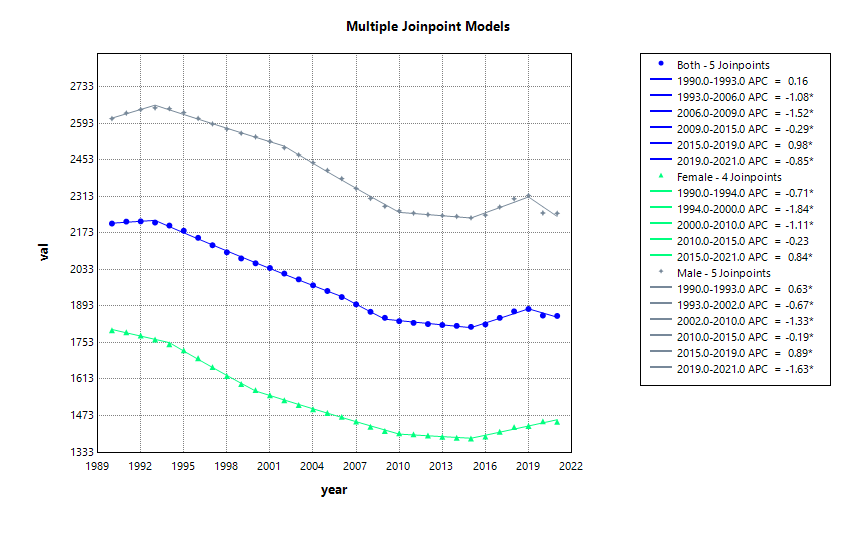 |

**Figure S16** Joinpoint regression analysis results of drug use disorder: Disability-adjusted life years, prevalence, and years lived with disability by two age groups and sex. APC, annual percentage change; DALYs, disability-adjusted life-years; YLDs, years lived with disability; All prevalence estimates in this study refer to point prevalence; * means significance

|  | **DALYs** | **Prevalence** |
| --- | --- | --- |
| **Age**  **10-19** | **A** | **B** |
|  | 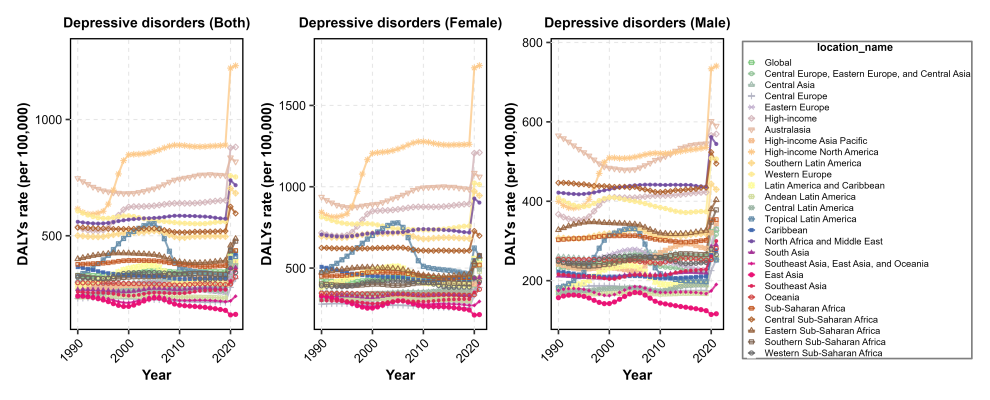 | 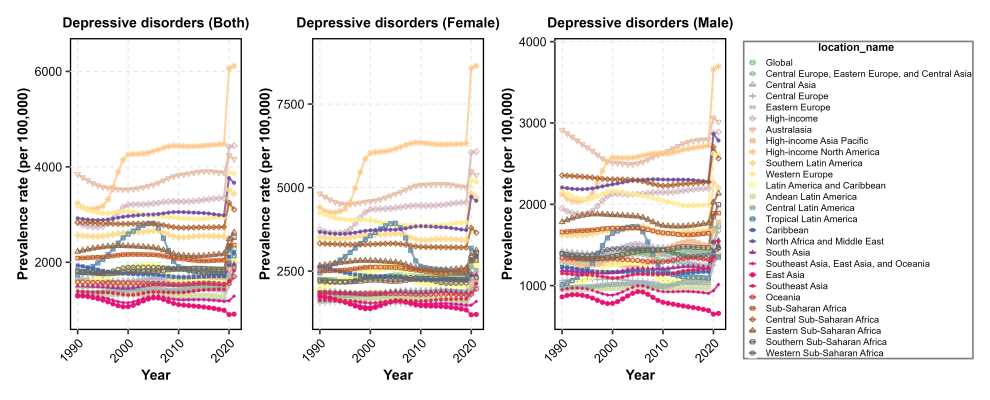 |
| **Age**  **20-24** | **C** | **D** |
|  | 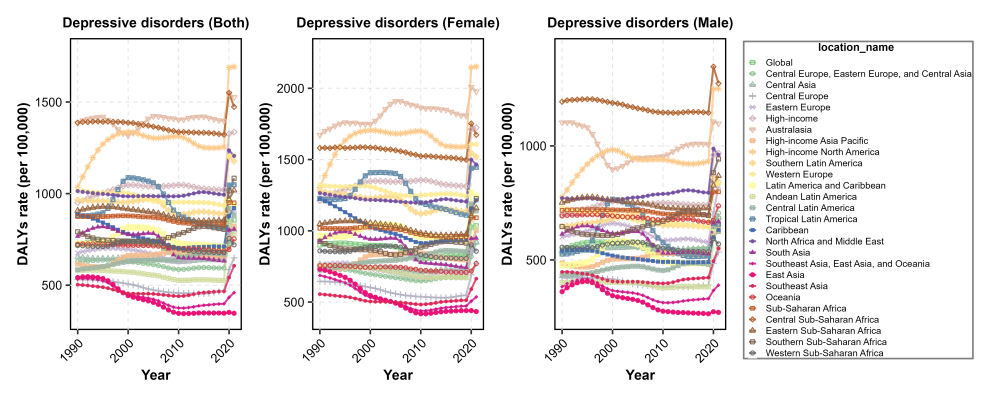 | 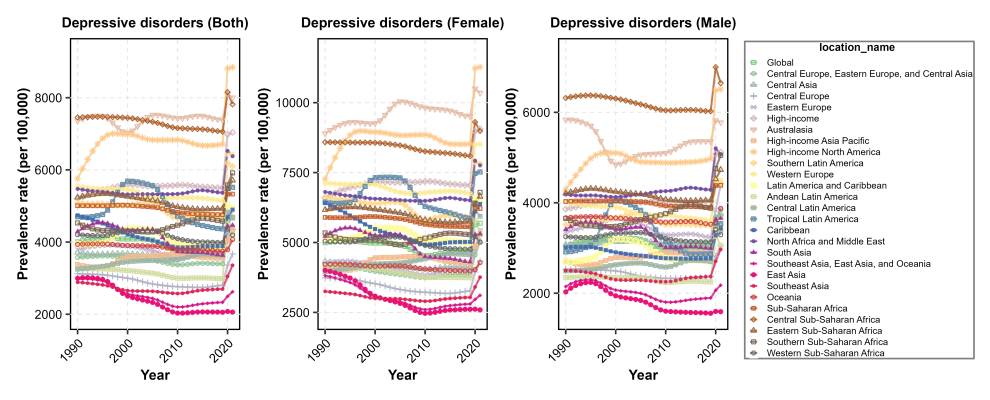 |

**Figure S17** Trends in disability-adjusted life years and prevalence of depressive disorders from 1990 to 2021, by two age groups, sex, and regions. All prevalence estimates in this study refer to point prevalence

|  | **DALYs** | **Prevalence** |
| --- | --- | --- |
| **Age**  **10-19** | **A** | **B** |
|  | 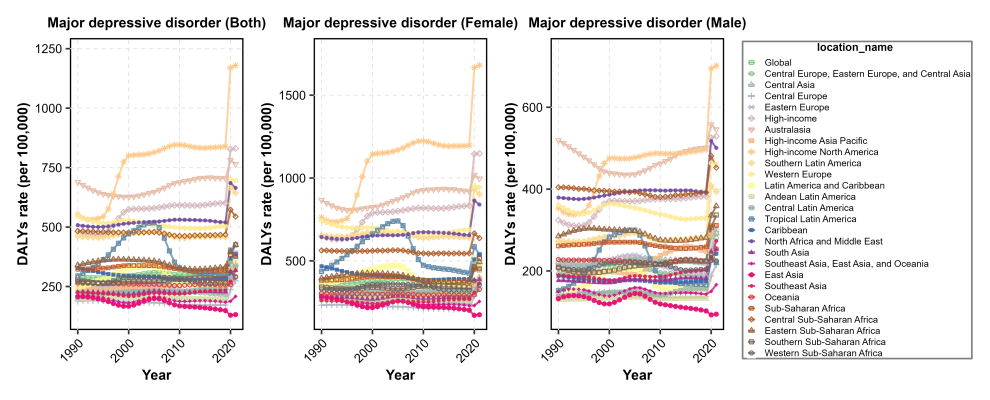 | 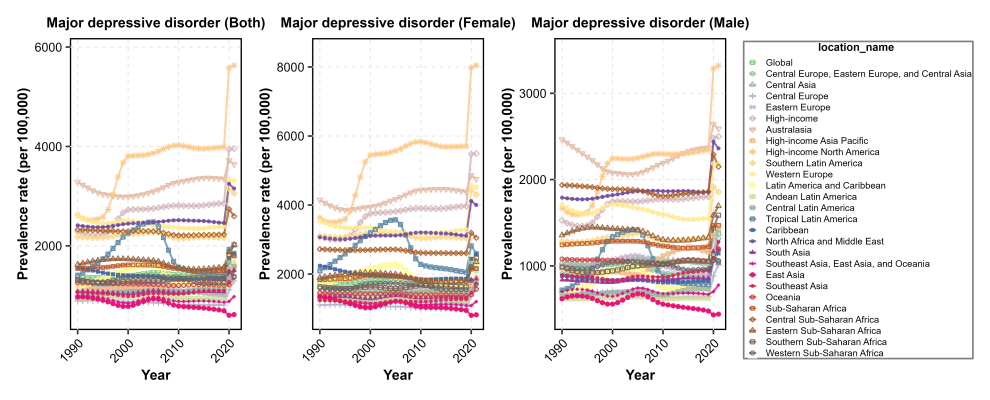 |
| **Age**  **20-24** | **C** | **D** |
|  | 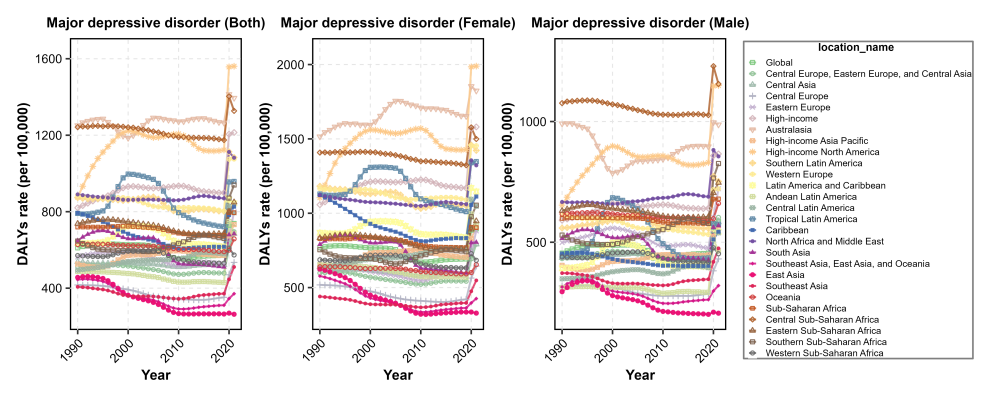 | 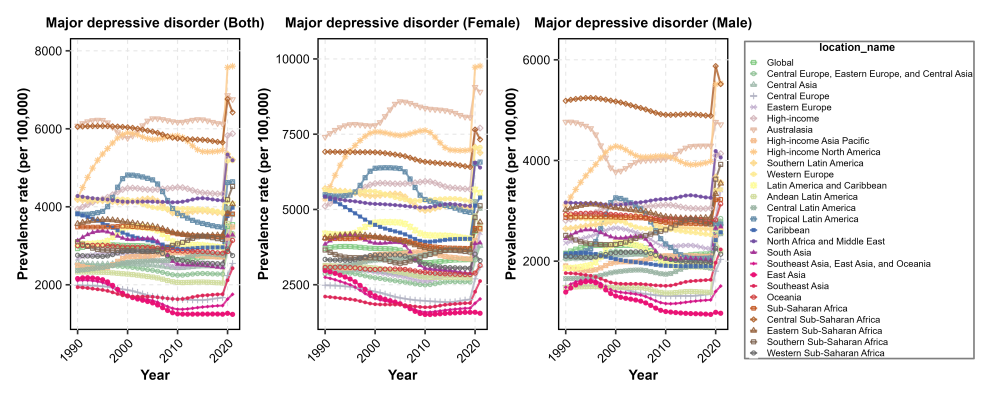 |

**Figure S18** Trends in disability-adjusted life years and prevalence of major depressive disorder from 1990 to 2021, by two age groups, sex, and regions. All prevalence estimates in this study refer to point prevalence

|  | **DALYs** | **Prevalence** |
| --- | --- | --- |
| **Age**  **10-19** | **A** | **B** |
|  | 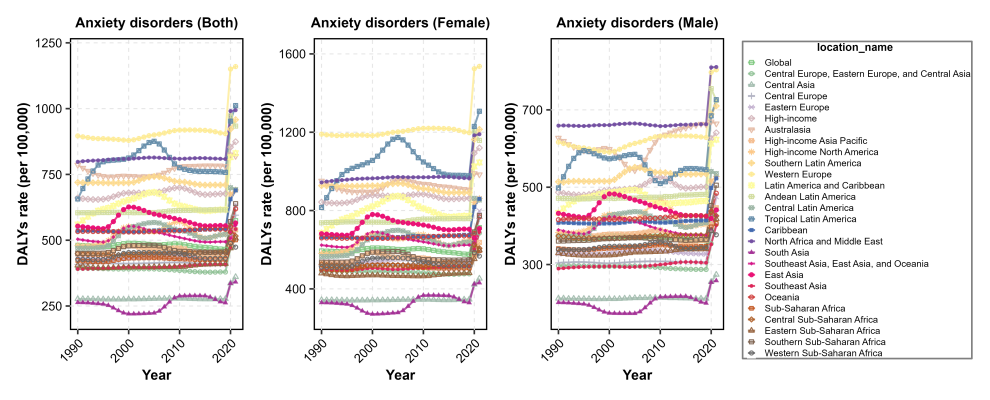 | 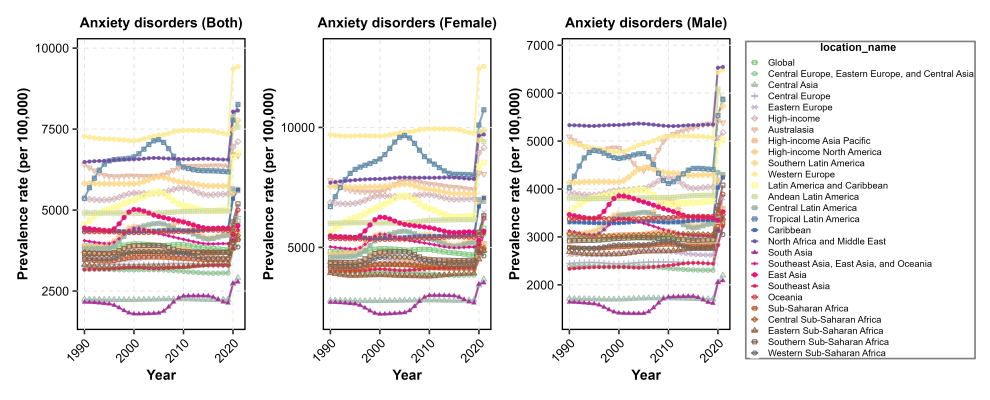 |
| **Age**  **20-24** | **C** | **D** |
|  | 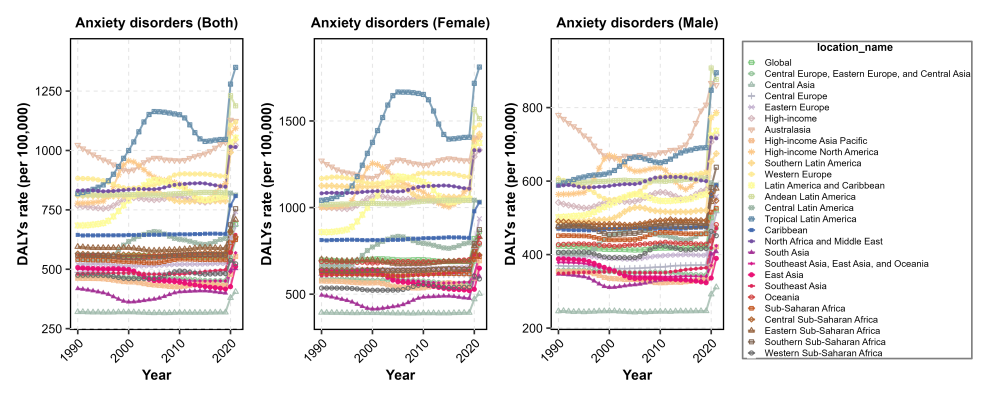 | 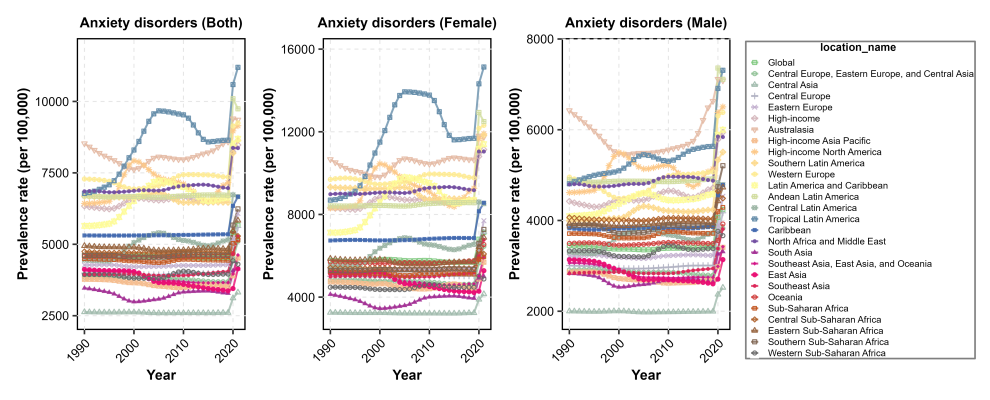 |

**Figure S19** Trends in disability-adjusted life years and prevalence of anxiety disorders from 1990 to 2021, by two age groups, sex, and regions. All prevalence estimates in this study refer to point prevalence

|  | **DALYs** | **Prevalence** |
| --- | --- | --- |
| **Age**  **10-19** | **A** | **B** |
|  | 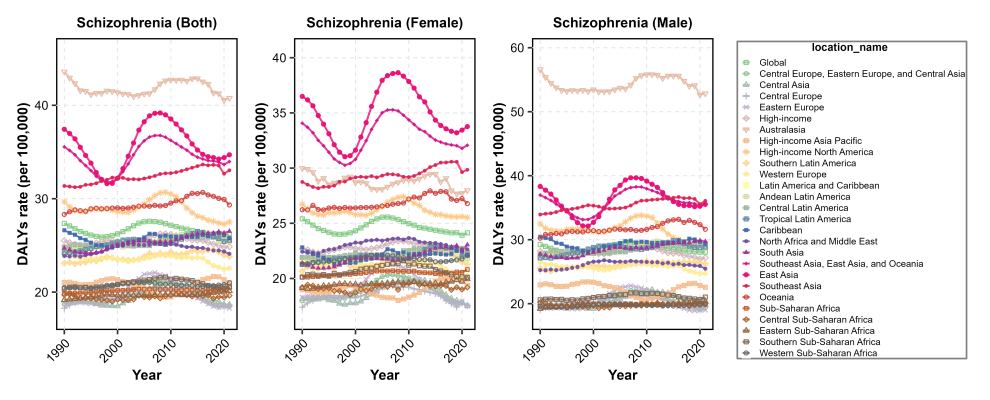 | 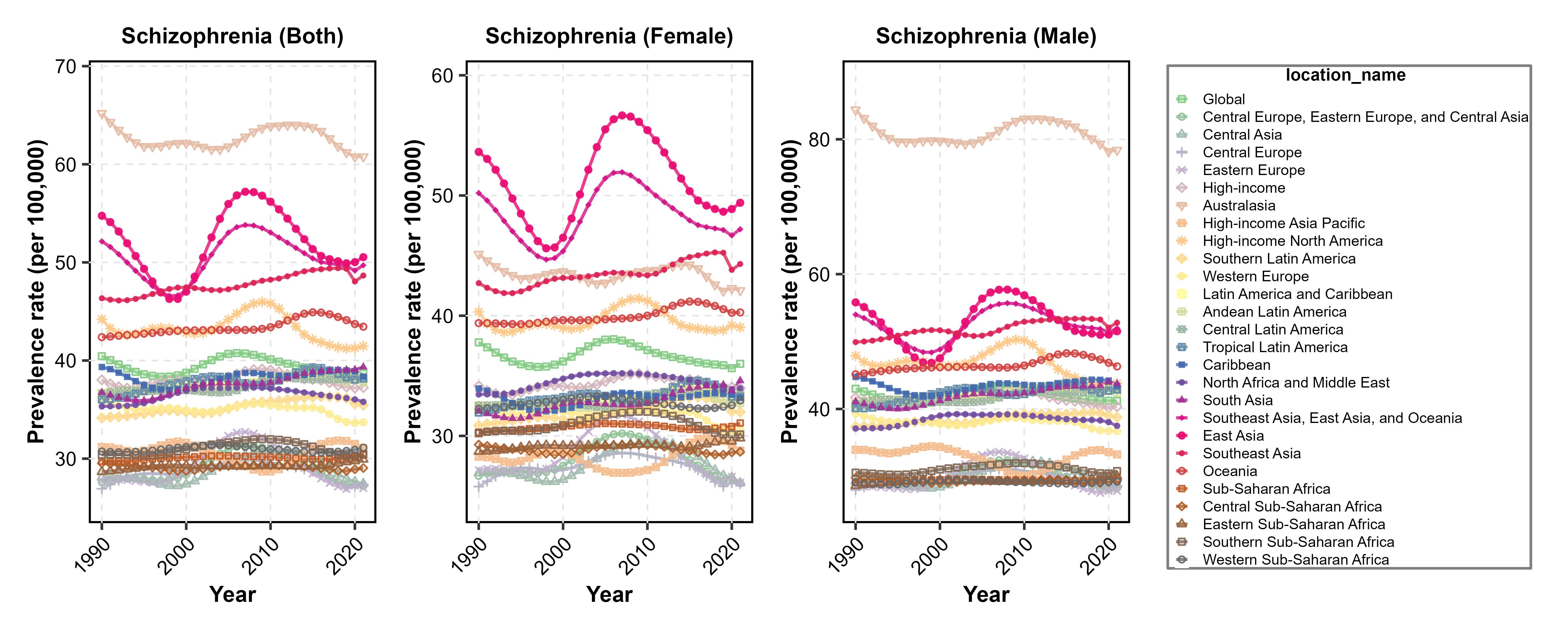 |
| **Age**  **20-24** | **C** | **D** |
|  | 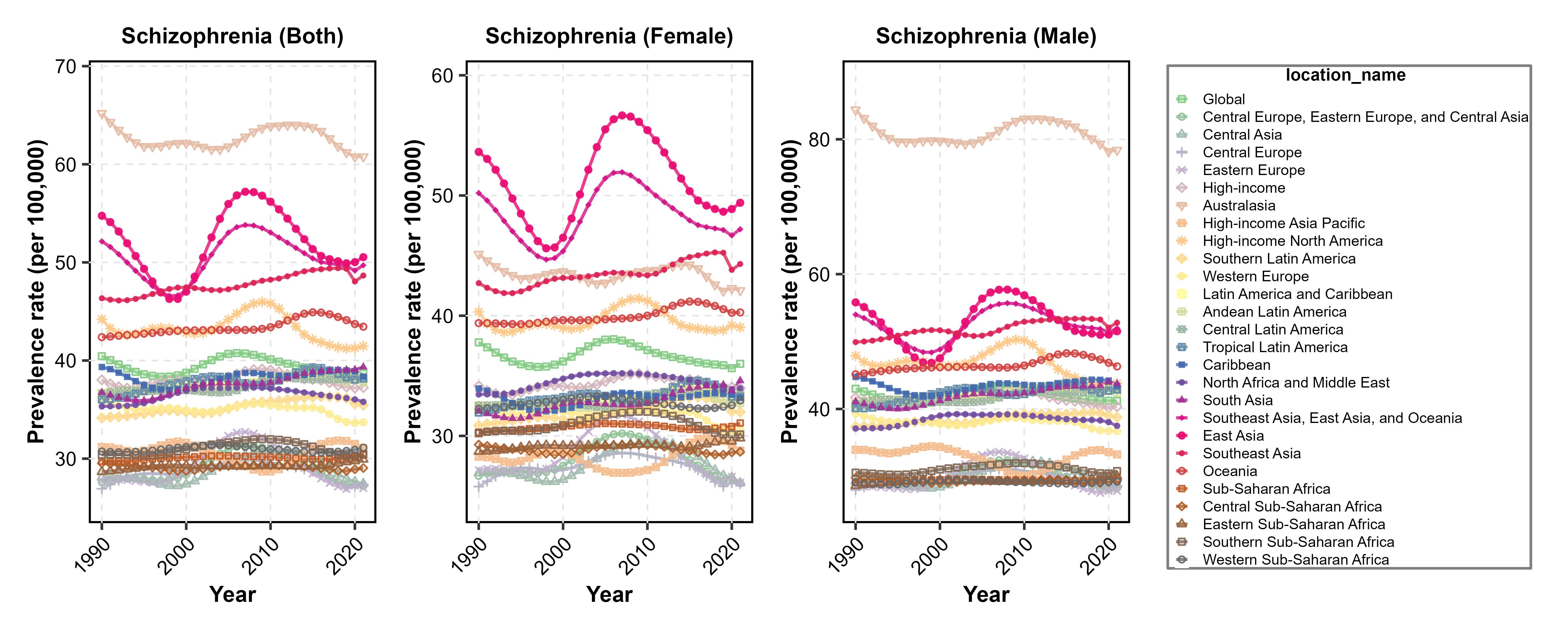 | 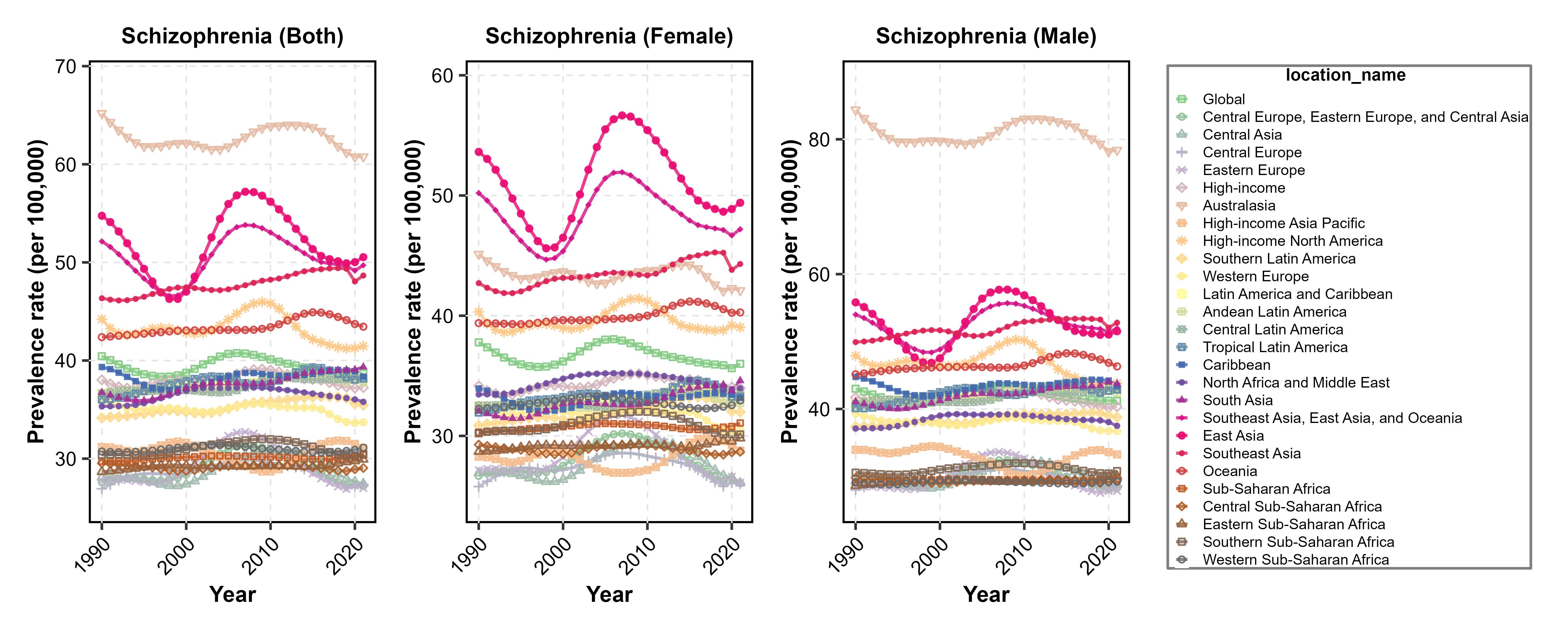 |

**Figure S20** Trends in disability-adjusted life years and prevalence of schizophrenia from 1990 to 2021, by two age groups, sex, and regions. All prevalence estimates in this study refer to point prevalence

|  | **DALYs** | **Prevalence** |
| --- | --- | --- |
| **Age**  **10-19** | **A** | **B** |
|  | 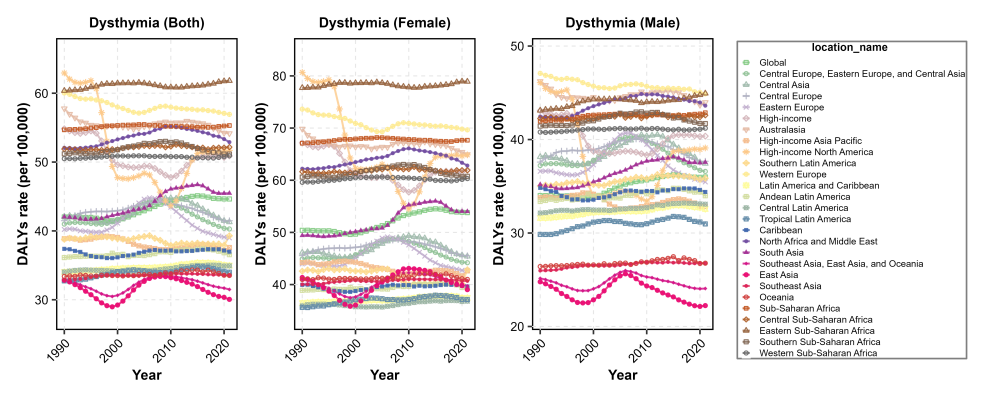 | 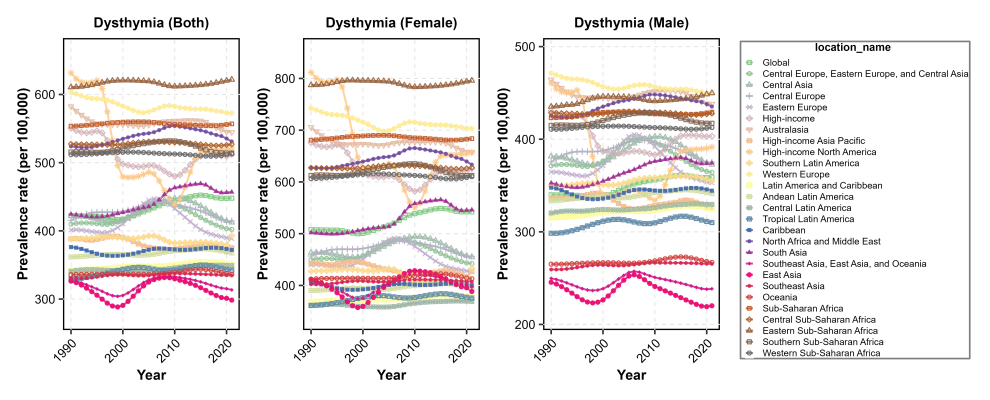 |
| **Age**  **20-24** | **C** | **D** |
|  | 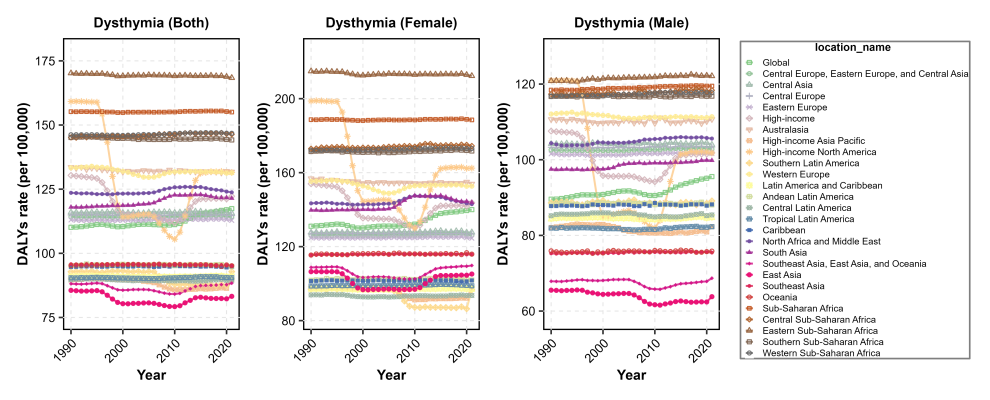 | 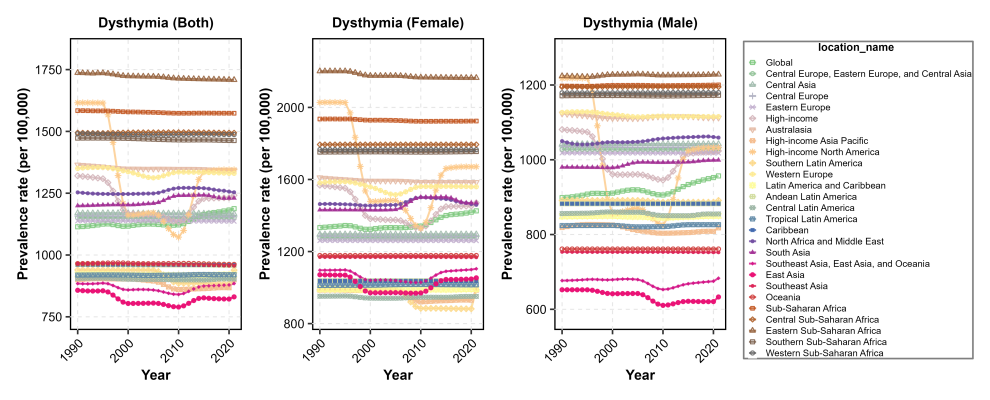 |

**Figure S21** Trends in disability-adjusted life years and prevalence of dysthymia from 1990 to 2021, by two age groups, sex, and regions. All prevalence estimates in this study refer to point prevalence

|  | **DALYs** | **Prevalence** |
| --- | --- | --- |
| **Age**  **10-19** | **A** | **B** |
|  | 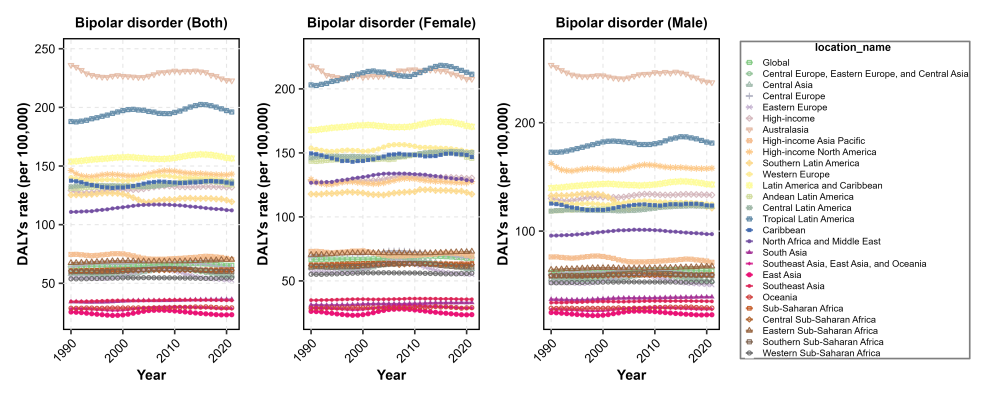 | 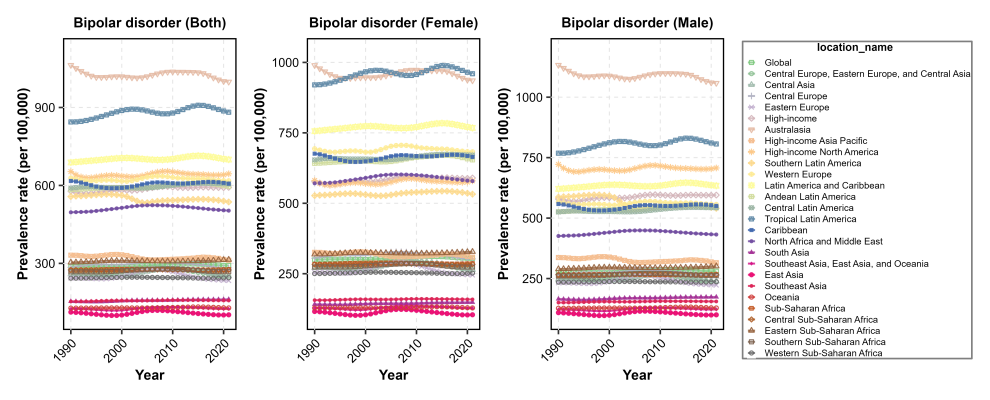 |
| **Age**  **20-24** | **C** | **D** |
|  | 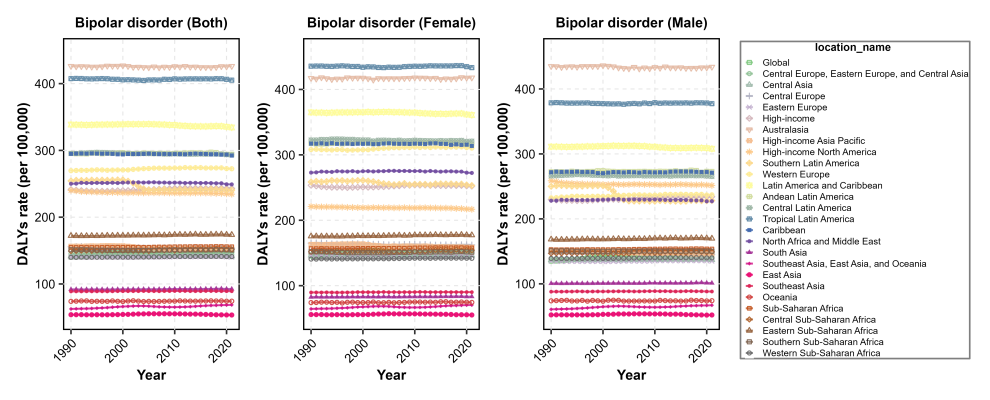 | 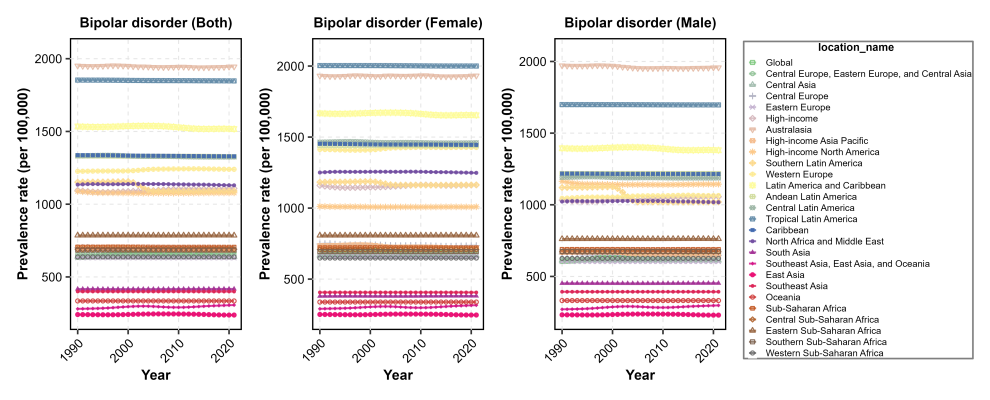 |

**Figure S22** Trends in disability-adjusted life years and prevalence of bipolar disorder from 1990 to 2021, by two age groups, sex, and regions. All prevalence estimates in this study refer to point prevalence

|  | **DALYs** | **Prevalence** |
| --- | --- | --- |
| **Age**  **10-19** | **A** | **B** |
|  | 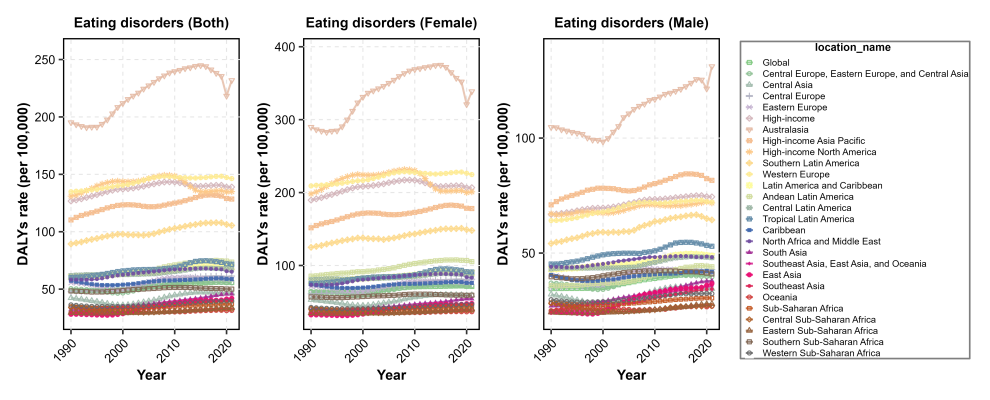 | 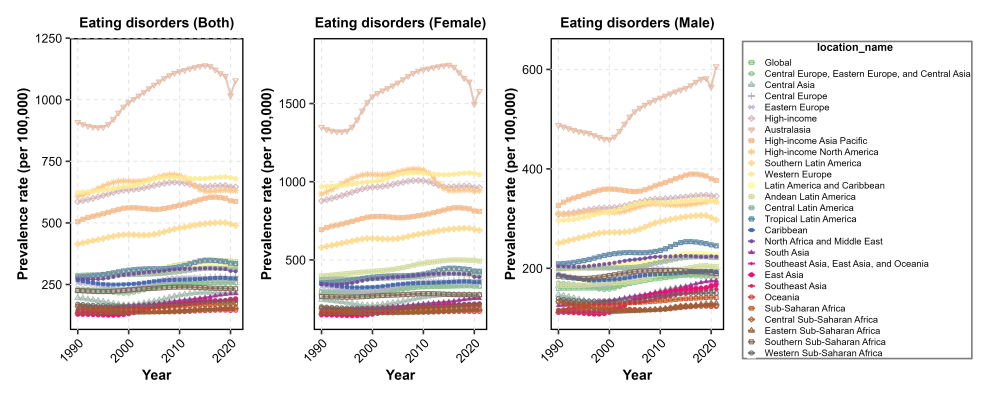 |
| **Age**  **20-24** | **C** | **D** |
|  | 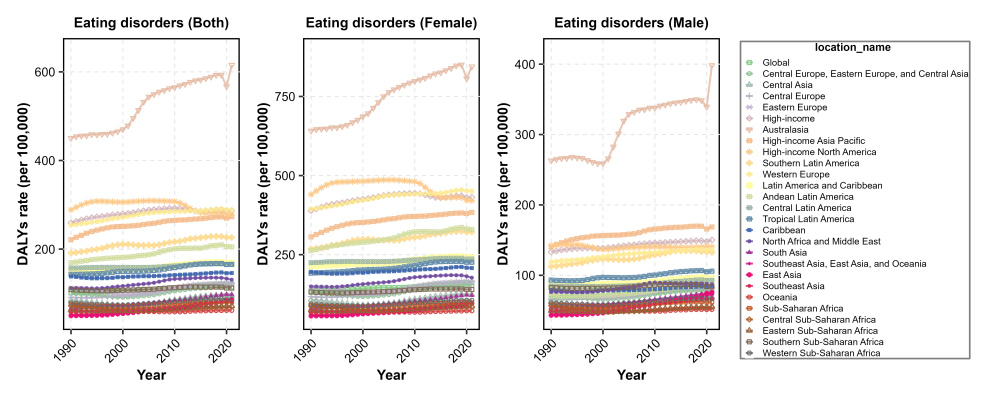 | 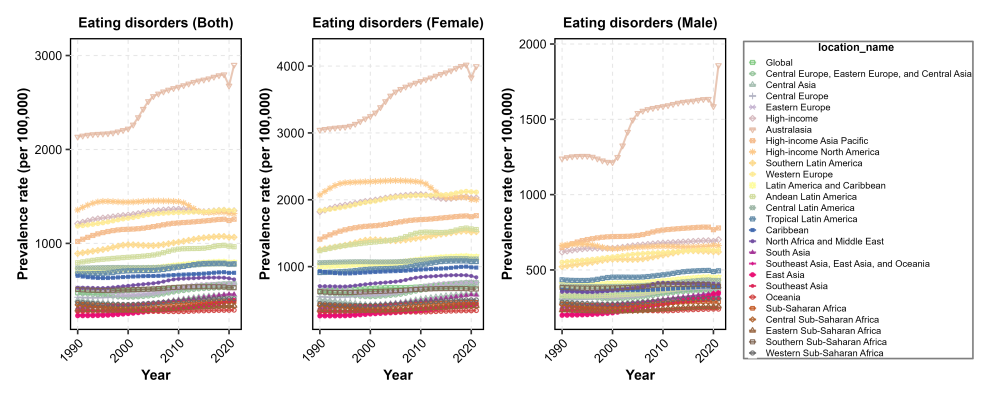 |

**Figure S23** Trends in disability-adjusted life years and prevalence of eating disorders from 1990 to 2021, by two age groups, sex, and regions. All prevalence estimates in this study refer to point prevalence

|  | **DALYs** | **Prevalence** |
| --- | --- | --- |
| **Age**  **10-19** | **A** | **B** |
|  | 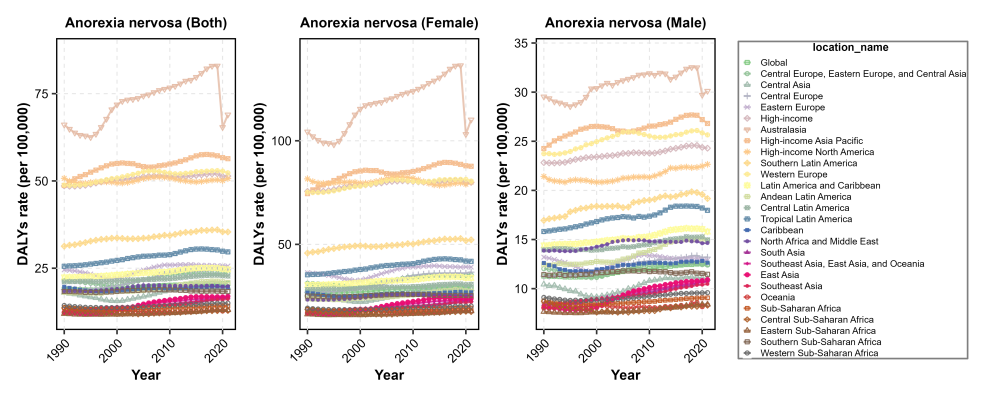 | 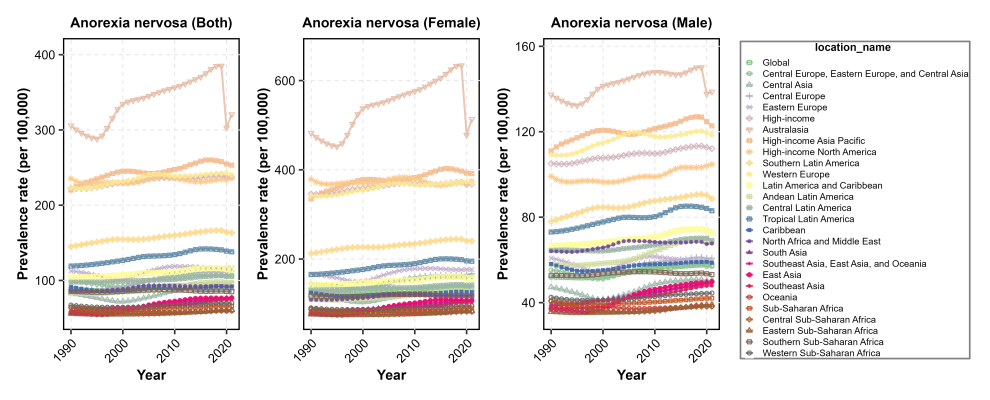 |
| **Age**  **20-24** | **C** | **D** |
|  | 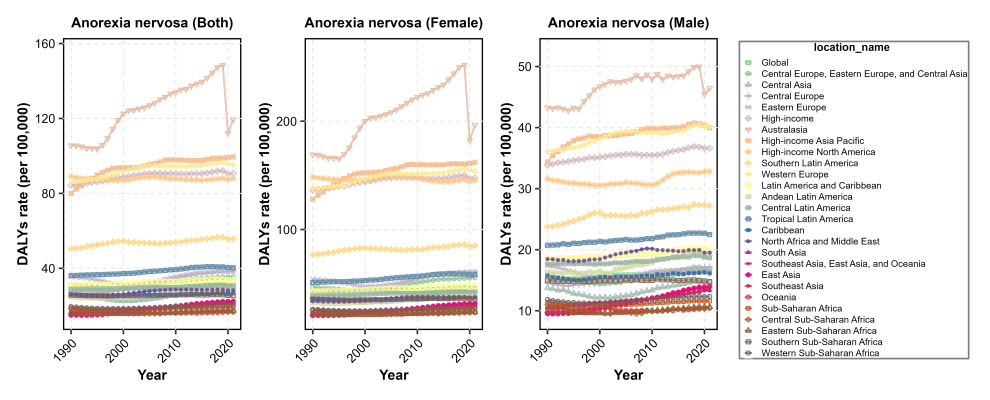 | 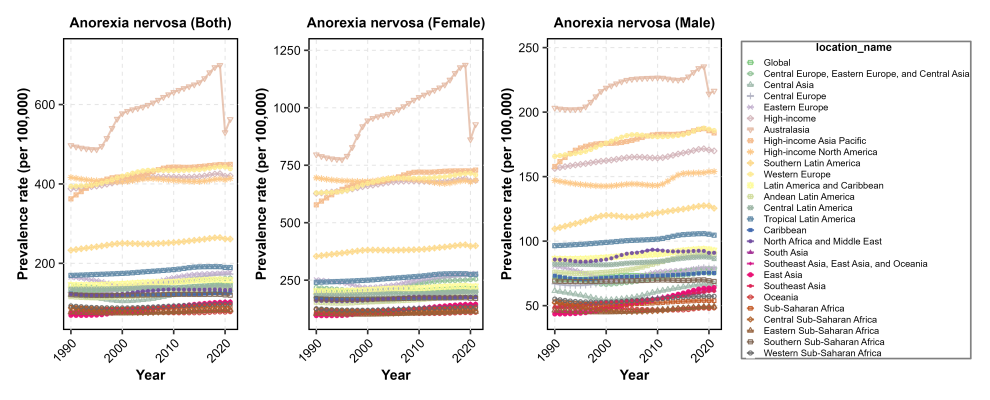 |

**Figure S24** Trends in disability-adjusted life years and prevalence of anorexia nervosa from 1990 to 2021, by two age groups, sex, and regions. All prevalence estimates in this study refer to point prevalence

|  | **DALYs** | **Prevalence** |
| --- | --- | --- |
| **Age**  **10-19** | **A** | **B** |
|  | 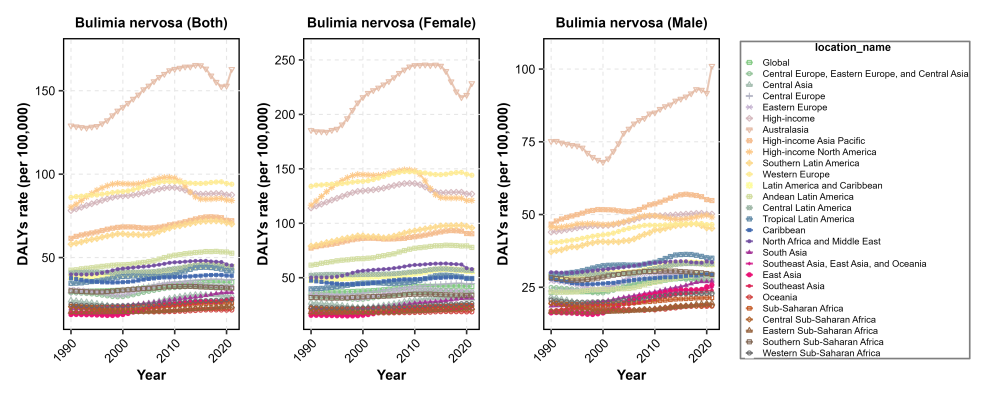 | 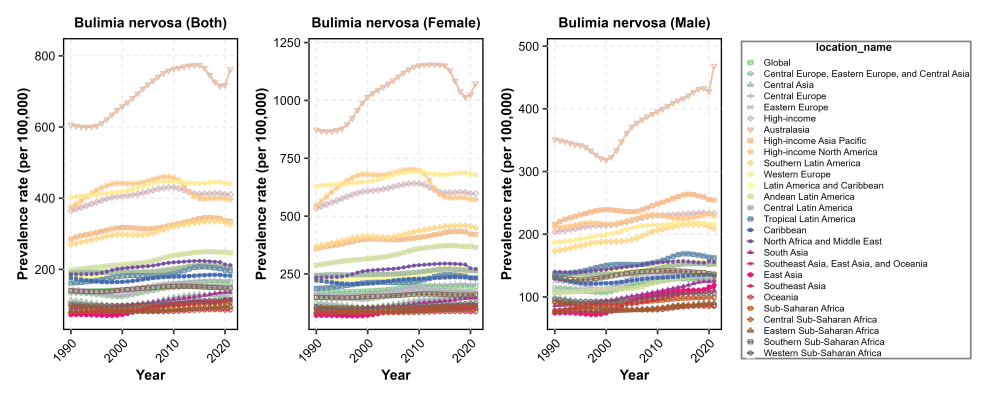 |
| **Age**  **20-24** | **C** | **D** |
|  | 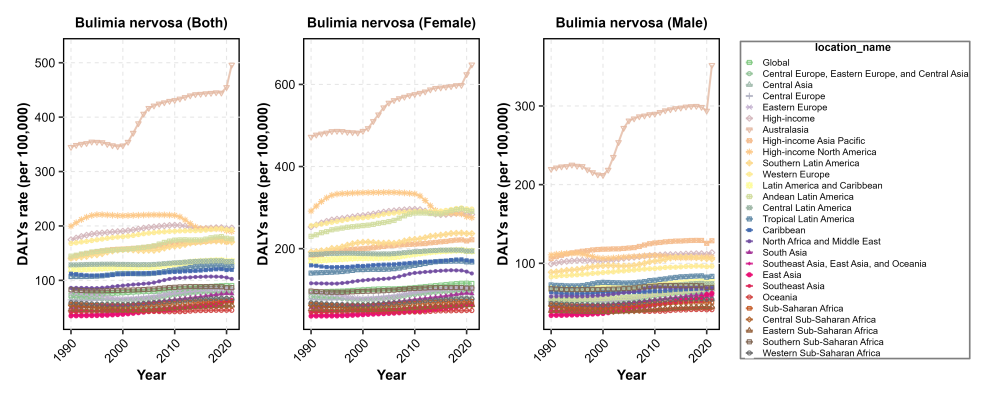 | 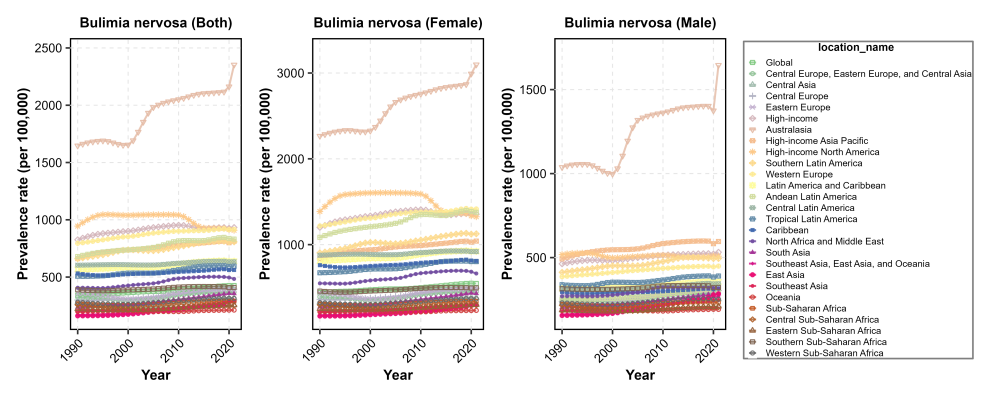 |

**Figure S25** Trends in disability-adjusted life years and prevalence of bulimia nervosa from 1990 to 2021, by two age groups, sex, and regions. All prevalence estimates in this study refer to point prevalence

|  | **DALYs** | **Prevalence** |
| --- | --- | --- |
| **Age**  **10-19** | **A** | **B** |
|  |  |  |
| **Age**  **20-24** | **C** | **D** |
|  |  |  |

**Figure S26** Trends in disability-adjusted life years and prevalence of autism spectrum disorders from 1990 to 2021, by two age groups, sex, and regions. All prevalence estimates in this study refer to point prevalence

|  | **DALYs** | **Prevalence** |
| --- | --- | --- |
| **Age**  **10-19** | **A** | **B** |
|  |  |  |
| **Age**  **20-24** | **C** | **D** |
|  |  |  |

**Figure S27** Trends in disability-adjusted life years and prevalence of attention-deficit/hyperactivity disorder from 1990 to 2021, by two age groups, sex, and regions. All prevalence estimates in this study refer to point prevalence

|  | **DALYs** | **Prevalence** |
| --- | --- | --- |
| **Age**  **10-19** | **A** | **B** |
|  |  |  |
| **Age**  **20-24** | **C** | **D** |
|  |  |  |

**Figure S28** Trends in disability-adjusted life years and prevalence of conduct disorder from 1990 to 2021, by two age groups, sex, and regions. All prevalence estimates in this study refer to point prevalence

|  | **DALYs** | **Prevalence** |
| --- | --- | --- |
| **Age**  **10-19** | **A** | **B** |
|  |  |  |
| **Age**  **20-24** | **C** | **D** |
|  |  |  |

**Figure S29** Trends in disability-adjusted life years and prevalence of idiopathic developmental intellectual disability from 1990 to 2021, by two age groups, sex, and regions. All prevalence estimates in this study refer to point prevalence

|  | **DALYs** | **Prevalence** |
| --- | --- | --- |
| **Age**  **10-19** | **A** | **B** |
|  |  |  |
| **Age**  **20-24** | **C** | **D** |
|  |  |  |

**Figure S30** Trends in disability-adjusted life years and prevalence of other mental disorders from 1990 to 2021, by two age groups, sex, and regions. All prevalence estimates in this study refer to point prevalence

|  | **DALYs** | **Prevalence** |
| --- | --- | --- |
| **Age**  **10-19** | **A** | **B** |
|  |  |  |
| **Age**  **20-24** | **C** | **D** |
|  |  |  |

**Figure S31** Trends in disability-adjusted life years and prevalence of drug use disorders from 1990 to 2021, by two age groups, sex, and regions. All prevalence estimates in this study refer to point prevalence

|  | **DALYs** | **Prevalence** |
| --- | --- | --- |
| **Age**  **10-19** | **A** | **B** |
|  |  |  |
| **Age**  **20-24** | **C** | **D** |
|  |  |  |

**Figure S32** Trends in disability-adjusted life years and prevalence of alcohol use disorders from 1990 to 2021, by two age groups, sex, and regions. All prevalence estimates in this study refer to point prevalence
